# Glycerol-3-phosphate O-acyltransferase is required for PBAN-induced sex pheromone biosynthesis in *Bombyx mori*

**DOI:** 10.1038/srep08110

**Published:** 2015-01-29

**Authors:** Mengfang Du, Xiaoguang Liu, Xiaoming Liu, Xinming Yin, Shuangyin Han, Qisheng Song, Shiheng An

**Affiliations:** 1 State key Laboratory of Wheat and Maize Crop Science/College of Plant Protection, Henan Agricultural University, Zhengzhou 450002 P.R. China; 2 Translational Research Center, Zhengzhou University People's Hospital, Zhengzhou 450003 P.R. China; 3 Division of Plant Sciences, University of Missouri, Columbia, Missouri, United States of America

## Abstract

Female moths employ their own pheromone blends as a communicational medium in mating behavior. The biosynthesis and release of sex pheromone in female moths are regulated by pheromone biosynthesis activating neuropeptide (PBAN) and the corresponding action of PBAN has been well elucidated in *Bombyx mori.* However, very little is known about the molecular mechanism regarding the biosynthesis of sex pheromone precursor. In this study, quantitative proteomics was utilized to comprehensively elucidate the expression dynamics of pheromone glands (PGs) during development. Proteomic analysis revealed a serial of differentially expressed sex pheromone biosynthesis-associated proteins at the different time points of *B. mori* development. Most interestingly *B. mori* glycerol-3-phosphate O-acyltransferase (BmGPAT) was found to be expressed during the key periods of sex pheromone biosynthesis. RNAi knockdown of *BmGPAT* confirmed the important function of this protein in the biosynthesis of sex pheromone precursor, triacylglcerol (TAG), and subsequently PBAN-induced production of sex pheromone, bombykol. Behavioral analysis showed that RNAi knockdown of *GPAT* significantly impaired the ability of females to attract males. Our findings indicate that GPAT acts to regulate the biosynthesis of sex pheromone precursor, TAG, thus influencing PBAN-induced sex pheromone production and subsequent mating behavior.

In general, female lepidopteran moths use species-specific sex pheromones to lure the conspecific males for successful mating and subsequent reproduction. Lepidopteran sex pheromones are *de novo* synthesized in the pheromone glands (PGs) from acetyl-CoA through fatty acid biosynthesis followed by desaturation, chain-shortening, fatty acyl reduction and carbonyl carbon modification^1^. The biosynthesis and release of sex pheromones in moths were first proposed to be controlled by a neuroendocrine factor^2^. This was late confirmed in *Helicoverpa zea* in which a neuropeptide, named pheromone biosynthesis activating neuropeptide (PBAN), was found to regulate the biosynthesis and release of sex pheromone^3^. PBAN regulates either fatty acyl reduction step or prior to fatty acid biosynthesis (likely acetyl coenzyme A carboxylase) dependent on the moth species^4^,^5^.

Bombykol is the first sex pheromone discovered and identified from the PGs of the silkworm moth *Bombyx mori*, located between the eighth and ninth segments of the ovipositor tip of females, which produce and release bombykol, (E, Z)-10, 12-hexadecadien-1-ol, as a major component of sex pheromones upon adult emergence^6^. Similar to other moths, *B. mori* synthesizes and releases bombykol under the regulation of PBAN. The actions of PBAN in *B. mori* have been well investigated and a series of associated genes involved in this process have been identified and characterized in detail^5^. Bombykol is synthesized in PG cells from acetyl-CoA via the fatty acid biosynthesis pathway. The biosynthetic fatty acid is converted into bombykol via the action of PG-specific desaturase 1 (pgdesat1) and PG-specific fatty-acyl reductase. Knockdown of the genes encoding pgdesat1 and PG-specific fatty-acyl reductase reduces bombykol production. This finding indicates that these two genes serve important functions in bombykol biosynthesis^7^,^8^. Before adult emergence, PG cells rapidly produce numerous bombykol precursors, lipid droplets (LDs) in the form of triacylglycerols (TAGs) stored in cytoplasm. A diacylglycerol acyltransferase 2 gene encoding for a speed-limiting enzyme of TAG biosynthesis was previously identified in *B. mori* PGs. RNAi knockdown of this gene significantly decreases bombykol production, suggesting that diacylglycerol acyltransferase 2 plays an important role in storage of TAGs, bombykol precursors^9^. After adult emergence, PBAN stimulates the lipolysis and subsequent reduction of LD TAGs to generate final bombykol^10^. Our previous study confirmed the high expression of seven lipase genes in PG cells through digital gene expression profiling. RNAi knockdown of four of the seven lipase genes individually leads to reduced bombykol production, suggesting that these four lipase genes are involved in the lipolysis process of TAGs^11^,^12^. A similar function of PBAN induced movement of the stored pheromone precursor fatty acids in *B. mori* is also found in *Manduca sexta*^13^.

The lipolysis of TAGs was also studied in impressive detail in *B. mori*. PBAN activates the calcium influx upon binding to its receptor (i.e. a G-protein coupled receptor)^14^. Ca^2+^/calmodulin-dependent protein kinase II (BmCaMKII), activated by PBAN-mediated calcium influx, phosphorylates lipid storage droplet protein-1 (Lsd-1), a homologue of perilipin in mammalians. The phosphorylation of Lsd-1 facilitates and promotes the release of the lipase activator complex to activate TAG lipase for the generation of bombykol precursors^10^. In addition, several key proteins involved in *B. mori* sex pheromone biosynthesis were identified, such as fatty acid transport protein, which facilitates the uptake of extracellular long-chain fatty acids across the plasma membrane^15^, Acyl-CoA binding proteins (ACBPs), which protect fatty acyl-CoA esters from hydrolysis and ensure enough fatty acid precursors for bombykol biosynthesis, stromal interaction molecule I (STIMl) and store-operated channel protein (Orail: including OrailA and OrailB), which are essential components of the signal transduction cascade regulating bombykol production^5^,^16^. Despite previous efforts, the essential components involved in biosynthesis of bombykol precursor and activation of lipases for TAG lipolysis have yet to be identified. In the present study, quantitative proteomics, molecular biology and behavior analysis were combined to investigate the molecular mechanism regarding the synthesis of sex pheromone precursor. The results showed that glycerol-3-phosphate O-acyltransferase (GPAT) is required for TAG biosynthesis and subsequent bombykol production in *B. mori*, thus, providing insights into the PBAN-regulated pheromone biosynthesis in *B. mori.*

## Results

### Identification of differentially expressed proteins

In *B. mori*, PG is formed at 72 h before emergence (−72 h), begins to produce sex pheromone at emergence (0 h) and maintains to release sex pheromone until 72 h after emergence (72 h). Thus these 3 time points (−72 h, 0 h and 72 h) in *B. mori* development were chosen for proteomic analysis. iTRAQ-based quantitative proteomic analysis was performed to determine the differentially expressed proteins at the different time points of PG development in *B. mori*. A total of 10117 peptides matched to 1523 proteins with scores higher than 1.3 (confidence level of 95%) and FDRs lower than 0.01.

**Figure 1:**
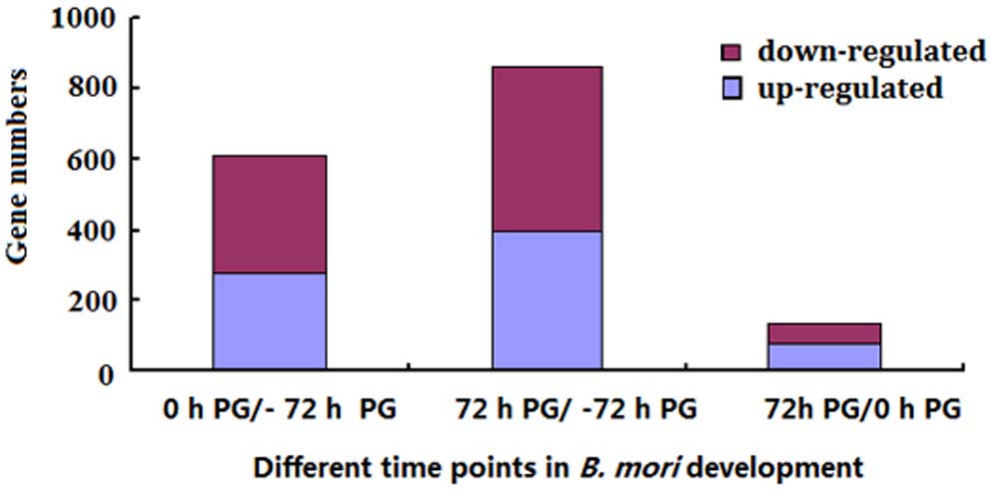
Differentially expressed proteins in PGs of different time points of *B. mori* development.

**Figure 2:**
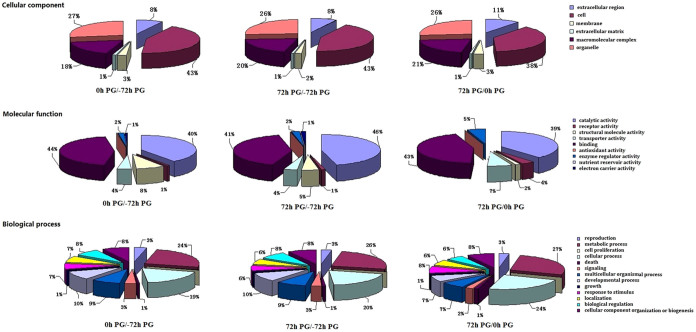
Gene ontology (GO) term distributions (the second level) for three categories. GO analysis was performed using the Cytoscape software ver. 2.6.2 according the differentially expressed proteins.

Proteins were considered differentially expressed when they showed a 1.2-fold change at the *p* < 0.05 level in the different samples (0 h PG vs. −72 h PG, 72 h PG vs. 0 h PG and 72 h PG vs. −72 h PG). A total of 612 proteins were differentially expressed between the 0 h PG and −72 h PG samples (Fig. 1, Table S1), with 276 up-regulated and 336 down-regulated proteins. Further GO classification analysis of these proteins was performed. For biological processes, 24%, 19% and 10% of the differentially expressed proteins were correlated with metabolic processes, cellular processes and developmental processes respectively. For molecular function, 44% and 40% of the differentially expressed proteins were associated with binding and catalytic activity respectively. For cellular components, 43% and 27% were related to cells and organelles, respectively (Fig. 2).

Comparative analysis of the 72 h and −72 h PG samples revealed that 863 proteins were significantly expressed with 396 up-regulated proteins and 467 down-regulated proteins (Fig. 1, and Table S1). Further GO classification analysis of these proteins was performed. For biological processes, 26%, 20% and 10% of the differentially expressed proteins were correlated with metabolic processes, cellular processes and developmental processes, respectively. For molecular functions, 46% and 41% of the differentially expressed proteins were associated with catalytic and binding activities, respectively. For cellular components, 43% and 26% of the differentially expressed proteins were related to cells and organelles, respectively (Fig. 2).

A total of 137 proteins showed significant changes between the 72 and 0 h PG samples, with 80 up-regulated and 57 down-regulated proteins (Fig.1, and Table S1). Further GO classification analysis of these proteins was performed. For molecular functions, 43% and 39% of the differentially expressed proteins were associated with binding and catalytic activities, respectively. For cellular components, 38% and 21% of the differentially expressed proteins were related to cells and macromolecular matrix, respectively (Fig. 2).

### Identification and quantitative real-time PCR (qPCR) verification of sex pheromone biosynthesis-associated proteins

**Figure 3:**
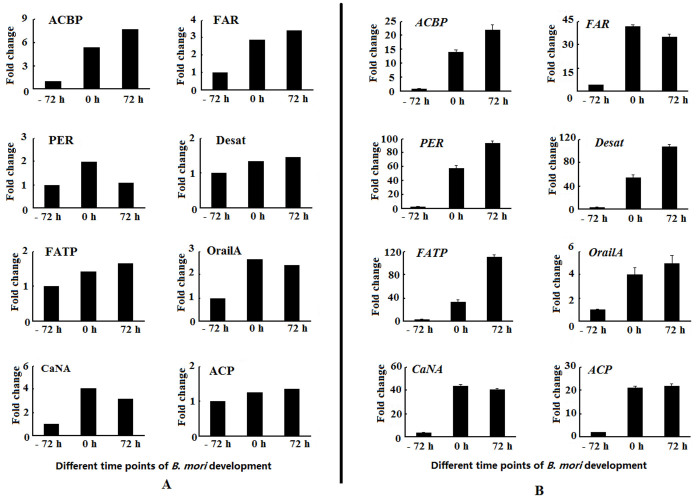
The relative expression levels of sex pheromone biosynthesis-associated proteins based on proteomic data (A) and genes (B) in PGs of different time points in *B. mori* development. PGs at different time points in *B. mori* development were dissected and total RNA was extracted for qPCR analysis. The Rp49 gene was used as the housekeeping gene for normalization. The data represent the mean values ± SD of three biological replicates. FATP: fatty acid transport protein, Desat:acyl CoA desaturase, ACBP: acyl-CoA binding protein, FAR: fatty-acyl reductase, PER: perilipin, OrailA: Orai1 alternative splice form A, CaNA: calcineurin A, ACP: acyl carrier protein.

A series of sex pheromone biosynthesis-associated proteins were highly expressed in 0 h and 72 h PG samples, which are crucial for the biosynthesis and release of sex pheromones (Table 1). These proteins include fatty acid transport protein, acyl CoA desaturase, acyl-CoA binding protein, fatty-acyl reductase, perilipin, Orai1 alternative splice form A, calcineurin A and acyl carrier protein. These proteins indicated the biosynthesis and release of sex pheromones in PGs and further confirmed the reliability of quantitative proteomic analysis. qPCR results manifested that the expression levels of these proteins (Fig. 3A) were consistent with their transcript levels (Fig. 3B).

### Expression analysis of glycerol-3-phosphate O-acyltransferase (GPAT) gene

**Figure 4:**
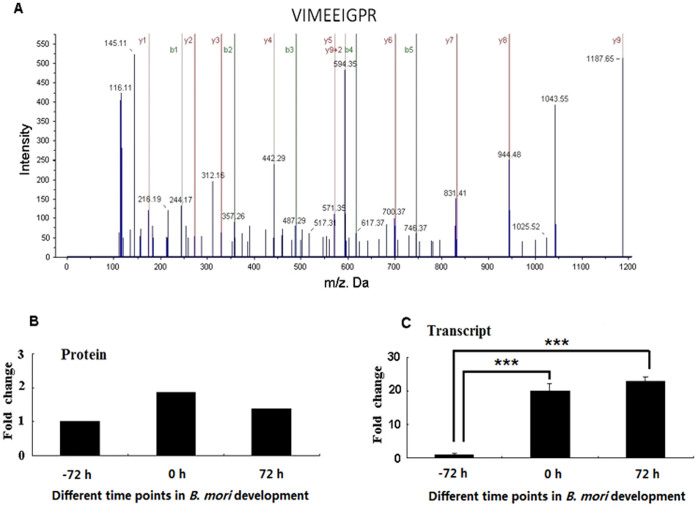
Characterization of glycerol-3-phosphate O-acyltransferase (GPAT). (A) The MS/MS spectrum for a peptide fragment sequence (VIMEEIGPR) of GPAT. (B) GPAT protein level based on proteomics results and (C) GPAT transcript level in different time points in *B. mori* development. PGs at different time points in *B. mori* development were dissected and total RNA was extracted for qPCR analysis. The Rp49 gene was used as the housekeeping gene for normalization. The data represent the mean values ± SD of three biological replicates. Significance of comparisons are marked with *** (*p* < 0.01) as determined by the Student's *t*-test.

Proteomic analysis identified a polypeptide sequence, VIMEEIGPR, encoded by *GPAT* gene (Fig. 4A). Since GPAT catalyzes the first step of TAG biosynthesis pathway by KEGG analysis, thus GPAT was chosen to be the target gene for further study. The protein expression profile based on proteomics revealed that GPAT was highly expressed in the 0 h and 72 h PG samples, compared with the expressed level of GPAT at −72 h PG samples (Fig. 4B). Further qPCR results manifested that *GPAT* was highly expressed in the 0 h and 72 h PG samples, compared with the expressed level of *GPAT* at −72 h PG samples (Fig. 4C).

### RNAi-mediated knockdown of *GPAT*: effect on cytoplasmic LD dynamics and TAG content

**Figure 5:**
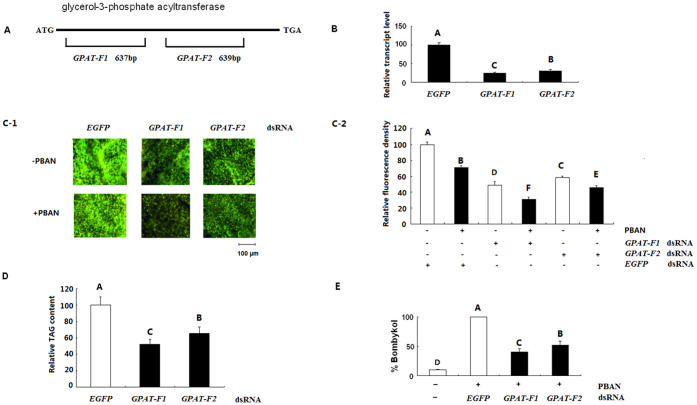
Functional analysis of BmGPAT. (A) Schematic diagram for depicting the location of dsRNA matched to GPAT opening reading frame. (B) qPCR analysis of effects of different dsRNA injection on GPAT transcript level. The rp49 gene was used as the housekeeping gene for normalization in all qPCR analysis. The data represent the mean ± SD of three biological replicates. Significance of comparisons are marked by the different capital letter (ANOVA and Tukey's test, *p* < 0.01). (C) Effects of RNAi-mediated knockdown of GPAT on LD dynamics. dsRNA (15 μg for each insect) corresponding to different regions of GPAT were injected into the decapitated females 48 h before elcosion, and then injected with 10 pmol PBAN or PBS buffer only for 90 min after a 48 h maintenance period. The EGFP dsRNA was used as negative control. The PGs were dissected for Nile red staining and subsequent photograph. The relative fluorescence density was measured using Quantity One soft. Statistically significant differences are denoted by the different capital letter (ANOVA and Tukey's test, *p* < 0.01). (D) Effects of RNAi-mediated knockdown of *GPAT* on PG TAG production. The PGs were dissected 48 h after dsRNA injection as described above and extracted for TAG quantification. Bars indicate the mean ± SD of three biological replicates for independent experimental animals (n > = 12). Statistically significant differences from the EGFP dsRNA–injected females are denoted by the different capital letter determined by ANOVA and Tukey's test (*p* < 0.01). (E) Effects of RNAi-mediated knockdown of GPAT on bombykol production. The moths 48 h after dsRNA injection as described above were injected individually with PBAN (10 pmol) and maintained for 90 min. The PGs were then dissected and extracted in hexane for bombykol quantification with GC/MS. Bars indicate the mean ± SD of three biological replicates for independent experimental animals (n > = 12). Statistically significant differences from the PBAN alone are denoted by the different capital letter (ANOVA and Tukey's test, *p* < 0.01).

The dsRNA corresponding to two regions of *GPAT* (*GPAT-F1* and *GPAT-F2*) was synthesized to elucidate the function of GPAT (Fig. 5A). When 15 μg dsRNA of GPAT was injected into female pupa 48 h before eclosion, the mRNA level of GPAT in PGs significant decreased 48 h after injection, compared with that in pupa injected with control EGFP dsRNA (Fig. 5B). GPAT-F1 dsRNA had higher interference efficiency than GPAT-F2 dsRNA.

Bombykol precursors are stored in the form of TAGs within cytoplasmic LDs. The LDs release the bombykol precursor fatty acid in response to the external PBAN signal. The PGs were stained with Nile Red to monitor the LD dynamics after the successful suppression of GPAT transcript by RNAi. Nile Red staining revealed that PBAN treatment significantly reduced the fluorescence brightness, indicating reduced LD production. The female PGs injected with GPAT dsRNA substantially accumulated fewer LDs compared with the control females injected with EGFP dsRNA (Fig. 5C). Further TAG content analysis manifested that the female PGs injected with GPAT dsRNA synthesized fewer TAGs compared with the control females injected with EGFP dsRNA (Fig. 5D). These results suggested that GPAT significantly affected the biosynthesis of TAGs and LDs. In addition, GPAT-F1 dsRNA had stronger inhibitory effect than GPAT-F2 dsRNA. These results were in agreement with the findings of RNAi efficiency.

### RNAi-mediated knockdown of *GPAT*: effect on bombykol production

After the successful reduction of GPAT mRNAs and TAG content by RNAi, the effect of GPAT dsRNA on subsequent PBAN-induced bombykol production was determined using GC/MS. RNAi significantly reduced bombykol production by approximate 50% (Fig. 5E). GPAT-F1 dsRNA had stronger suppression effect than GPAT-F2 dsRNA. This result was consistent with the results of RNAi efficiency and cytoplasmic LD dynamics.

### RNAi-mediated knockdown of *GPAT*: effect on female ability to attract males

**Figure 6:**
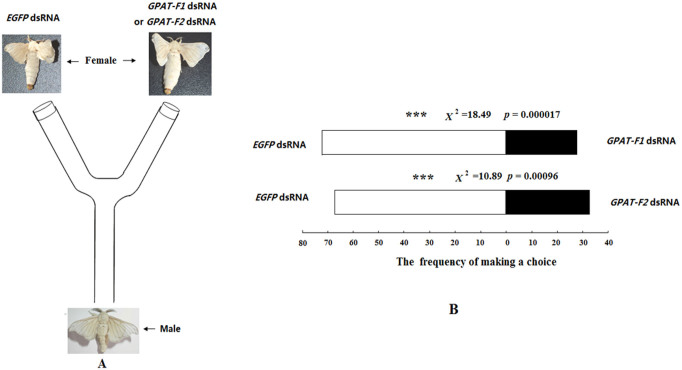
Schematic drawing of the modified Y-tube olfactometer (A) and the choice frequency of male moths to the GPAT- knockdown females and EGFP control (B). dsRNA (15 μg for each insect) corresponding to different regions (*GPAT-F1* and *GPAT-F2*) of *GPAT* were injected into the decapitated females 48 h before elcosion (EGFP was used as control). After 48 h maintenance, newly-emerged female were placed at Y-tube arm (EGFP as control) for luring the males and newly-emerged males were used to test the choice response to different female moth pre-injected with *GPAT-F1* dsRNA or *GPAT-F2* dsRNA 48 h before elcosion as treatment and female moth pre-injected with EGFP dsRNA 48 h before elcosion as control. The asterisks indicate significant difference between GPAT- knockdown females and EGFP control in the choice frequency of males (Chi-square statistics). *** *p* < 0.001.

The effect of RNAi knockdown of GPAT mRNAs on female ability to attract males was analyzed by using Y-tube olfactometer (Fig. 6A). The results showed that the GPAT knockdown significantly reduced female's ability to attract males when tested against EGFP control (GPAT-F1:EGFP = 28:72; GPAT-F2:EGFP = 33:67) (Fig. 6B), similar results were also found in the reciprocal experiments of Y-tube left and right portions (data not shown).

## Discussion

In most moths, the biosynthesis and release of sex pheromone are precisely regulated by PBAN. The PBAN signal transduction in *B. mori* has been elucidated in impressive detail for complete genomics information of *B. mori*. A series of genes responsible for sex pheromone biosynthesis and release have been well documented. Nonetheless, as a model species, the detail molecular mechanisms of *B. mori* sex pheromone biosynthesis are not fully elucidated. In this study, iTRAQ-based proteomics was employed to study the developmental expression profile of PGs, the targeted tissues of PBAN. The results revealed a series of differentially expressed proteins during PG development. These differentially expressed proteins, including fatty acid transport protein, acyl CoA desaturase, acyl-CoA binding protein, fatty-acyl reductase, perilipin, Orai1 alternative splice form A, calcineurin A and acyl carrier protein, were involved in the developmental regulation of sex pheromone biosynthesis and release. They served as marker proteins for the biosynthesis and release of sex pheromones, as being confirmed in *B. mori*. The proteomics results were consistent with the previous results, in which the transcripts of these proteins were richly expressed at key stages of sex pheromone biosynthesis^5^,^7^,^8^,^10^,^15^,^16^,^17^.

In *B. mori* PGs, LDs rapidly accumulated before adult emergence. These LDs contain various TAGs which provide the precursor fatty acids and are released for sex pheromone production in response to PBAN. The LDs fluctuate in both size and number in accordance with the fluctuation of PBAN release and bombykol amount^18^. These fluctuations in LD and bombykol amount could be prevented by decapitation and preceded by PBAN injection. The amount of TAGs also significantly decreased after PBAN application. These results clearly demonstrated that PBAN stimulated the lipolysis by activating lipases which hydrolyze TAGs for the release of bombykol precursor. LSD-1 was found to play an important role in TAG lipolysis associated with bombykol production in *B. mori*. PBAN-mediated calcium influx led to the activation of a series of components, including CaMKII, which activates LSD-1 phosophorylation. The activated LSD-1 then resulted in activation of the associated lipases. RNA Seq studies found that seven lipases were highly expressed in *B. mori* PGs at key stages of sex pheromone biosynthesis. RNAi-mediated knockdown confirmed that four of seven lipases are involved in TAG lipolysis^11^.

Since TAGs are the bombykol precursors, they play import roles in sex pheromone biosynthesis and release. However TAG biosynthesis and storage are rarely investigated. Our previous study revealed that diacylglycerol acyltransferase 2 (DGAT) catalyses the final step in TAG biosynthesis, RNAi knockdown of DGAT causes a substantial decrease in sex pheromone production^9^. However TAG biosynthesis consists of two pathways, namely the monoacylglycerol (MAG) pathway and glycerol phosphate pathway (GAP). The MAG and GAP pathways share a common final step which is controlled by DGAT. The results of proteomics show an increase in GPAT protein level at key stage of sex pheromone biosynthesis. GPAT catalyses the first step in TAG biosynthesis via GAP, namely the conversion of glycerol 3-phosphate and acyl-CoA into 1-acylglycerol-3- phosphate. GPAT has the lowest enzyme specific activity in this pathway, thus it has been considered to be a speed-limiting enzyme for TAG biosynthesis via GAP^19^. Four GPAT isoforms have been identified in mammalian animals. GPAT1 and GPAT2 are mitochondrial isoforms, and GPAT3 and GPAT4 are ER membrane isoforms^20^. The present iTRAQ-based proteomics identified a GPAT homologue in *B. mori* (BmGPAT) (Table 1). The homologue was highly expressed in the 0 and 72 h PG samples at the translational levels (Fig. 4B), this finding was consistent with the transcript profile of *BmGPAT* (Fig. 4C). Sequence analysis indicated that the amino sequence encoded by *BmGPAT* showed 31% identity with that of human GPAT1, suggesting that the *BmGPAT* identified in PGs was a GPAT1 isoform (data not shown).

In mammalian animals, the expression level of GPAT1 is the highest in liver and adipose tissues. GPAT activity has closely associated with the events which require *de nove* TAG biosynthesis. Thus the GPAT1 transcript increases by >20-fold in mouse liver once refed with a high-carbohydrate diet after starvation. By contrast, starvation for 48 h significantly reduces GPAT1 activity in rat liver and adipose tissues. This result indicates that GPAT1 is involved in TAG biosynthesis^19^. Mice with GPAT1 deficiency have reduced hepatic TAG, plasma TAG and VLDL–TAG secretion^21^. Even after feeding with high-fat diet, mice with GPAT1 deficiency have lower plasma and liver TAG and DAG contents than wild-type mice^22^. Correspondingly, the liver-directed overexpression of GPAT in mouse significantly increases hepatic TAG and DAG contents by 12- and 7-fold, respectively^23^. These results completely elucidated the function of GPAT in TAG biosynthesis. In the present study, expression analysis of BmGPAT in PGs revealed that BmGPAT was predominantly expressed during adult stages. The RNAi-mediated knockdown of the *BmGPAT* gene significantly reduced PG TAG contents (Fig. 5C; Fig. 5D), bombykol production (Fig. 5E) and female ability to attract males (Fig. 6B). This result suggested that BmGPAT play an import role in TAG biosynthesis, bombykol production and subsequent mating behavior in *B. mori*.

**Figure 7:**
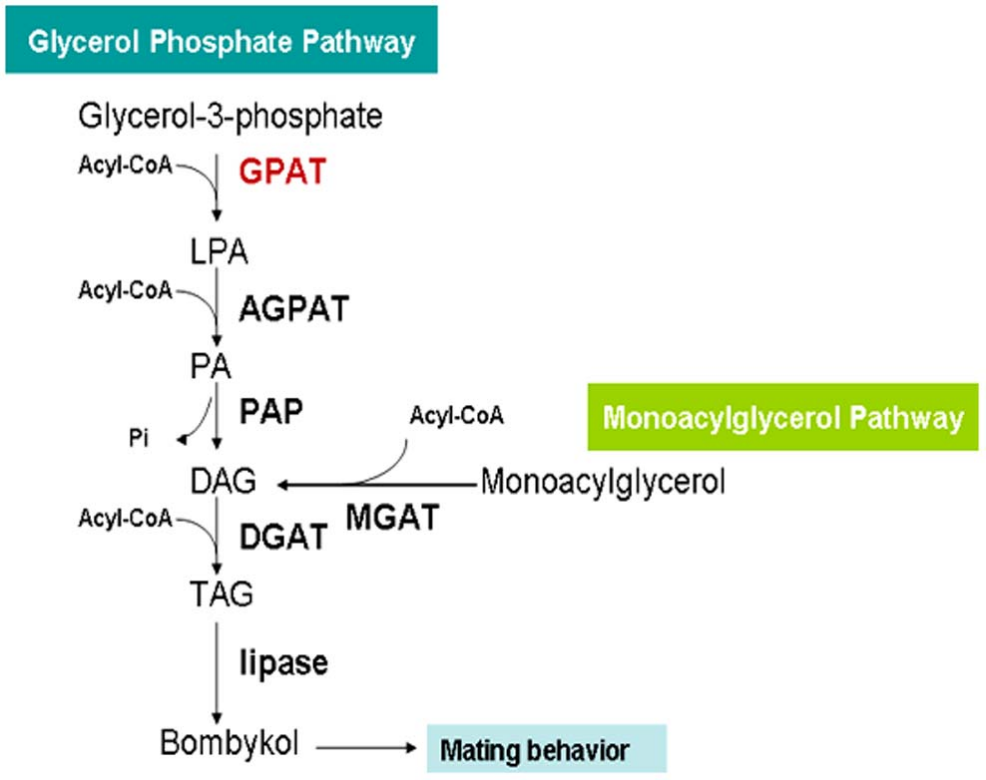
A proposed model showing the role of BmGPAT in modulating the biosynthesis of sex pheromone precursor and female sex pheromone production. AGPAT: 1-acylglycerol-3-phosphate acyltransferase, LPA: lysophosphatidic acid, PA: phosphatidic acid, PAP: phosphatidic acid phosphohydrolase, DAG: diacylglycerol, DGAT: diacylglycerol acyltransferase, TAG: triacylglycerol, MGAT: monoacylglycerol O-acyltransferase.

Our previous study identified a DGAT gene which regulates the biosynthesis of bombykol precursor, TAG^9^. The present study went further to demonstrate the role of GPAT, an upstream component of DGAT, in bombykol biosynthesis. Combined both findings, we conclude that bombykol precursor are synthesized and stored in the cytoplasm in the form of TAGs via the GAP pathway. Once stimulated by PBAN, the corresponding lipases promote TAG lipolysis and release the bombykol precursors for final sex pheromone biosynthesis (Fig. 7). In animals GPAT1 is regulated by sterol regulatory element-binding protein-l, insulin signal, etc. BmGPAT has been proved to be an important component for sex pheromone precursor biosynthesis. However the corresponding regulatory mechanism of BmGPAT in PGs remains elusive and requires further studies.

## Methods

### Insects

Larvae of *B. mori* (Zhenzhu × Chunlei) were reared on mulberry leaves at 26°C under a 16 h light/8 h dark cycle. Pupae were separated by gender, and adult females were stored in separate cages until emergence.

### Chemicals

The PBAN of *B. mori* was synthesized by Sangon Biotech (Shanghai) Co., Ltd. The sex pheromone component, Bombykol, was obtained from Shogo Matsumoto (RIKEN, Advanced Science Institute, Japan) and used as the internal standard for gas chromatography/mass spectrometry (GC/MS).

### Sample Preparation

PGs were dissected at −72 h, 0 h and 72 h. Each sample includes at least 300 PGs. The collected samples were completely homogenized with a STD buffer(4% SDS, 1 mM DTT, 150 mM Tris-HCl pH 8.0), heated at 100°C for 5 min and then cooled to room temperature. The supernatants were transferred to new tubes and the corresponding protein concentration was determined using the Bicinchoninic acid (BCA) assay protocol (Bio-Rad, Berkeley, CA).

### Digestion and iTRAQ labelling

Protein digestion was carried out following the protocol described by Wisniewski et al^24^ and the resulting peptide mixture was labeled using the 8-plex iTRAQ reagent according to the manufacturer's instructions (Applied Biosystems). Briefly, a total 200 μg protein/sample prepared in 30 μL STD buffer was diluted in 200 μL of UA buffer (800 mM urea, 150 mM Tris-HCI pH 8.0). Detergent, DTT and other low-molecular-weight components were removed using UA buffer by repeated ultrafiltration using Microcon 30 (30 kD MW cutoff). Then 100 μL of UA buffer (contain 50 mM iodoacetamide) was added to each filtrate, followed by 20 min incubation in the dark. After 10 min of centrifugation under the above conditions, the filters were washed three times with 100 μL UA buffer. Then, 100 μL of DS buffer (50 mM triethylammonium bicarbonate, pH 8.5) was added to the filters and centrifuged twice at 14000 g, each for 10 min. Finally 2 μg trypsin (Promega) in 40 μL of DS buffer was added to each filtrate and incubated overnight at 37°C. The resulting peptides were harvested by centrifugation. The peptide content was measured by UV light spectral density at 280 nm using an extinction coefficient of l.1 in 0.l% (g/L) solution which was calculated based on the frequency of tryptophan and tyrosine.

iTRAQ labelling was performed following the manufacturer's instructions (Applied Biosystems). One hundred microgram peptide for each sample was labelled with iTRAQ regent. The −72 h PG sample (100 μg) were labelled with reagents 113 and 114. The 0 h PG samples were labelled with reagents 115, 116 and 117. The 72 h PG samples were labelled with reagents 118, 119 and 121. The labeling reaction was performed at room temperature for 1 h, then pooled and dried by vacuum centrifugation.

### Strong Cationic-exchange (SCX) Chromatography Separation

The iTRAQ-labeled peptides were fractionated by SCX chromatography using the AKTA Purifier system (GE Healthcare). In brief, the peptide mixture was reconstituted with 2 mL buffer A (10 mM KH_2_P0_4_ in 25% of acetonitrile, pH 3.0), loaded into a polysulphoethyl 4.6 mm × 100 mm column (5 μm, 200 Å, PolyLC Inc, Maryland, USA.) and then eluted at a flow rate of 1 mL/min with a gradient of 0%–10% buffer B (10 mM KH_2_P0_4_ pH 3.0, 500 mM KCl and 25% acetonitrile) for 2 min, 10% to 20% buffer B for 25 min followed by 20% to 45% buffer B for 5 min and 50% to 100% buffer B for 5 min. The UV absorbance at 214 nm was monitored when the fractions were collected. The fractions were collected every minute (about 30 fractions), combined in 10 pools and then desalted on C18 Cartridges [Empore SPE Cartridges C18 (standard density), 7 mm inner diameter, 3 mL volumes, Sigma]. Each fraction was concentrated by vacuum centrifugation and reconstituted in 40 μL of 0.1% formic acid for liquid chromatography-tandem mass spectrometry (LC-MS/MS).

### LC-Electrospray Ionization (ESI)-MS/MS Analysis by Q Exactive

Three technical replicates with respect to a Q Exactive mass spectrometer coupled to Easy nLC (Proxeon Biosystems, now Thermo Fisher Scientific) were set for better coverage of the target proteome with reliable statistical consistency. A 10 μL aliquot of each fraction was injected for nanoLC-MS/MS analysis. 5 μg of the peptide mixture was loaded into a the C18-reversed phase column (Zorbax 300SB-C18 peptide traps, Agilent Technologies, Wilmington, DE, 150 mm × 75 μm) packed with RP-C18 5 μm resin equilibrated for 20 min in buffer A (0.1% formic acid in water). The separation of peptide mixtures was performed with a linear gradient of buffer B (80% acetonitrile and 0.l% formic acid) at 250 nL/min rate controlled by IntelliFlow technology over 140 min. the gradient included 4% to 28% (v/v) for 110 min, 28% to 40% (v/v) for 20 min, 40% to 90% (v/v) for 5 min and 90% (v/v) for 5 min.

MS data acquisition was performed using the 10 most abundant precursor ions from the survey scan within the 300 m/z to 1800 m/z mass range for high-energy collisional dissociation (HCD) fragmentation. The target value was determined on the basis of predictive Automatic Gain Control (pAGC). Dynamic exclusion for selected precursor ions was set at 60 s. The resolution was set as follows: 70,000 at m/z 200 for MS scan and 17,500 at m/z 200 for HCD spectra. The normalized collision energy was 30 eV and the under fill ratio was defined as 0.1%. This ratio specifies the minimum percentage of the target value likely to be reached at the maximum fill time. The instrument was operated in peptide recognition mode enabled.

The protein identification and iTRAQ quantification were carried out as previously described using a MASCOT engine (Matrix Science, London, UK; version 2.2) embedded into Proteome Discoverer 1.3 (Thermo Electron, San Jose, CA.)^25^. Briefly, the database search was performed against the lepidopteran (Uniprot)database. The corresponding parameters were set as follows: Peptide mass tolerance = 20 ppm, MS/MS tolerance = 0.1 Da, Enzyme = Trypsin, Missed cleavage = 2, Fixed modification: Carbamidomethyl (C), iTRAQ 8plex(K), iTRAQ8plex(N-term), Variable modification:Oxidation(M), thorough search effort and detected protein threshold(False discovery rate, FDR) < 0.01. The ProGroup algorithm in the software was used to eliminate redundancy from the grouping of identified proteins.

The metabolic pathways of the identified proteins were analyzed according to the KEGG pathway database (http://www.genome.jp/kegg/pathway.html). Gene Ontology (GO) analyses (http://www.geneontology.org) were conducted according to a previously reported method^26^. For the differential protein expression, GO functional enrichment and KEGG pathway analyses were performed using the Cytoscape software version 2.6.2 (http://www.cytoscape.org/).

### qPCR

Total RNA was extracted from the harvested PG samples (n = 40) at different time points in *B. mori* development using Trizol reagent (Invitrogen, Carlsbad, CA) following the manufacturer's instructions. RNA concentration was further determined by spectrophotometer. Prior to first-strand cDNA synthesis, the purified RNA was treated with DNase for excluding genomic DNA contamination. First-strand cDNA synthesized from the total RNA (1 μg) of each PG sample using the PrimeScript RT reagent kit with gDNA Eraser (TaKaRa) was used as the template for qPCR. The primers for qPCR analysis are listed in Table S2. Since the ribosomal protein 49 (rp49) gene is the most stable in *B. mori* and has been widely used as a reference gene^27^, thus rp49 was chosen as a reference gene for normalization in our experiments. The efficiencies (E) of corresponding primers used in qPCR were calculated according to the equation: E = (10^[−1/slope]^−1) × 100^28^. qPCR was carried out using SYBR Green Supermix (TaKaRa) on an Applied Biosystems 7500 Fast Real-time PCR system (ABI, Carlsbad, CA, USA) according to the manufacturer's instructions. The thermal cycle conditions for qPCR were 95°C for 4 min, followed by 40 cycles of 95°C for 15 s and 60°C for 20 s. The specificity of the SYBR green PCR signal was further confirmed using agarose gel electrophoresis and melting curve analysis. The mRNA expression was quantified using the comparative Cross Threshold method (CT, the PCR cycle number that crosses the signal threshold)^29^. The CT of the rp49 gene was subtracted from the CT of the target gene to obtain ΔCT. The normalized fold changes of the target gene mRNA expression were expressed as 2^−ΔΔCT^, where ΔΔCT is equal to ΔCT_ treated sample_ −ΔCT_ control_.

### DsRNA synthesis

dsRNA was synthesized using the MEGAscript RNAi kit (Ambion) according to the manufacturer's instructions. The templates for dsRNA were prepared as previously described using gene-specific primers containing T7 polymerase sites^9^,^11^. All the primer sets are listed in Table S2. PCR was carried out as follows at 94°C for 3 min, 35 cycles of 94°C for 1 min, 59°C for 1 min, 72°C for 1 min, and a final elongation at 72°C for 10 min. The purified PCR product was used as template for *in vitro* dsRNA synthesis. The template DNA and single-stranded RNA were removed by DNase and RNase treatments followed by dsRNA purification using MEGAclearTM columns (Ambion). The synthesized dsRNA was then eluted in diethyl pyrocarbonate-treated nuclease-free water and the corresponding concentrations were measured using a biophotometer (Eppendorf). The dsRNA of enhanced green fluorescent protein (EGFP) was used as the negative control.

The effects of RNAi on the transcript expression were investigated by using qPCR. The corresponding primers are shown in Table S2.

### Injection of dsRNA and *in vivo* bombykol analysis

Fifteen micrograms of GPAT dsRNA was injected into the abdominal intersegment membrane of female pupae at 48 h before adult elcosion. The female pupae were maintained for 48 h until emergence under normal conditions. The newly emerged females were decapitated, maintained for 24 h and then injected with 10 pmol PBAN. The PGs were collected 90 min after the injection and then dissolved in hexane. Control females were injected with dsRNA of EGFP.

Bombykol accumulation was measured by GC/MS (Trace GC Ultra Trace DSQ; MS-Thermo Scientific DSQ II) equipped with a 30 m capillary column (RTX-5SILMS, Restek, 0.25 mm diameter). Each sample containing a pooled hexane extract from 12 or more *B. mori* PGs was subjected to GC/MS analysis.

### Staining of PG LDs

The PG samples were collected after dsRNA and PBAN treatments above mention, fixed with 4% formalin (dissolve in phosphate buffered saline), and then stained with Nile Red (Molecular probes, Eugene, OR) as previously described^30^. Fluorescence microscopy was performed with an Olympus BX-60 system. Nile Red images were captured and processed using Photoshop CS. Relative fluorescence brightness was determined by Quantity One soft (Bio-Rad). Corresponding results were compared using ANOVA and Tukey's test.

### Determination of TAG content

Fifteen micrograms of GPAT dsRNA was injected into the female pupae at 48 h before adult elcosion. The female pupae were maintained for 48 h until emergence under normal conditions. PGs were collected and rinsed in PBS. The lipid were extracted by using chloroform: methanol (v:v, 2:1) and then were used for TAG determination using a TAG assay kit (Rong Sheng Biotech Co., Ltd, Shanghai, China) following a previously described method^31^. Control females were injected with dsRNA of EGFP. Corresponding results were compared using ANOVA and Tukey's test.

### Effect of RNAi knockdown of *GPAT* on female ability to attract males

Female ability to attract males after RNAi knockdown of *GPAT* was analyzed by using a Y-tube olfactometer with 5 cm internal diameter and 15 cm long arms, set at a 45^0^ angles and 15 cm basal stem. 15 ug of dsRNA (*GPAT* or *EGFP*) was injected into the abdominal intersegment membrane of female pupae at 48 h before adult elcosion. Two treatments of newly emerged females were placed at two arms of Y-tube respectively. Newly emerged males were tested in the following olfactometer bioassays: 1) the female injected with GPAT-F1 dsRNA *vs* the female injected with EGFP dsRNA, 2) the female injected with GPAT-F2 dsRNA *vs* the female injected with EGFP dsRNA. For each of bioassays, 100 newly-emerged males were tested. The frequency of male making choice was recorded. A Yates corrected Chi-square test was used to determine differences between the frequencies of male choosing different female treatments (RNAi knockdown of *GPAT* or *EGFP* control).

### Statistical analysis

qPCR and GC/MS experiments were performed in triplicates. Results are expressed as means ± standard deviation (M ± SD). qPCR and GC/MS results were compared using student's *t-*tests.

## Supplementary Material

Supplementary Information Supplementary information

## Figures and Tables

**Table 1:** Differential expression proteins associated with sex pheromone biosynthesis and release

Accession #	Protein name	0 h PG/−72 h PG	*p* value	72 h PG/−72 h PG	*p* value	72 h PG/0 h PG	*p* value
tr|Q9GU82|	Acyl CoA desaturase	1.34	0.00083663	1.4634	0.004199	1.0916	0.06426
tr|Q9NBL4|	Acyl-CoA binding protein	3.25	0.00300	3.596	0.000153	1.107847	0.1737
tr|Q9NBL5|	Acyl-CoA binding protein	5.42297	0.0046	7.73124	0.00277	1.42565	0.03336
tr|Q7YTA8|	Fatty-acyl reductase	2.0297	0.00007945	2.01070	0.001115	0.99064	0.7944
tr|D2KMR4|	Lipase	1.278	0.0096	1.2660	0.07	0.9908	0.87647
tr|B3Y9F5|	Fatty acid transport protein	1.4399	0.0321	1.65972	0.000059	1.15267	0.00599
tr|B5BRC5|	Orai1 alternative splice form A	2.6702	0.0000200	2.4189	0.00171	0.9059	0.09913
tr|G6DEH3|	Calcineurin A	4.059	0.000074	3.15933	0.00017	0.77835	0.001
tr|H9JJZ4|	Acyl carrier protein	1.254	0.00412	1.3545	0.00767	1.08053	0.10502
tr|Q2F665|	Perilipin	1.99067	0.002	1.09633	0.22132	0.5747	0.00048
tr|G6D9L1|	Glycerol-3-phosphate acyltransferase	1.8806	0.0000087	1.352	0.0025	0.71892	0.000068
