# Supplementary material for: Glycerol-3-phosphate O-acyltransferase is required for PBAN-induced sex pheromone biosynthesis in Bombyx mori
Source: Sci Rep. 2015 Jan 29;5:8110. doi: 10.1038/srep08110 (PMC5389035; doi:10.1038/srep08110)
Supplement: Supplementary Information [file srep08110-s1.doc]

**Supplementary information**

**Glycerol-3-phosphate O-acyltransferase expression is required for PBAN-induced sex pheromone synthesis in *Bombyx mori***

Mengfang Du^1#^, Xiaoguang Liu^1#^, Xiaoming Liu^1^, Xinming Yin^1^, Shuangyin Han^2^, Qisheng Song^3^, and Shiheng An ^1^*

^1^ State key Laboratory of Wheat and Maize Crop Science/College of Plant Protection, Henan Agricultural University, Zhengzhou 450002 P.R. China

^2^ Translational Research Center, Zhengzhou University People's Hospital, Zhengzhou 450003 P.R. China

^3^ Division of Plant Sciences, University of Missouri, Columbia, Missouri, United States of America.

**Table S1** **Lists of the differentially expressed proteins**

| Accession | Description | Coverage | # Unique Peptides | Ratio( 0h PG/-72h PG) | t test P Value (0h PG/-72h PG) | ratio(72 h PG/-72h PG) | t test P Value (72 h PG/-72h PG) | ratio (72h PG/0 h PG) | t test P Value(72h PG/0 h PG) |
| --- | --- | --- | --- | --- | --- | --- | --- | --- | --- |
| Q9NBL5 | Acyl-CoA binding protein | 75.82 | 1 | 5.42297 | 0.0046 | 7.7312 | 0.0028 | 1.4256 | 0.033 |
| G6DEH3 | Calcineurin A | 16.16 | 8 | 4.059 | 7.36E-05 | 3.1593 | 0.0002 | 0.7784 | 1E-03 |
| S4P8L1 | Myosin heavy chain | 32.48 | 1 | 3.89875 | 0.004453 | 3.9277 | 0.006 | 1.0074 | 0.948 |
| Q9NBL4 | Acyl-CoA binding protein | 63.53 | 7 | 3.24645 | 0.003 | 3.5966 | 0.0002 | 1.1078 | 0.174 |
| S5SW83 | Isocitrate dehydrogenase | 2.93 | 1 | 3.1361 | 0.000874 | 4.9419 | 5E-05 | 1.5758 | 2E-04 |
| B8XE27 | Arginine kinase | 41.92 | 1 | 3.08057 | 0.002506 | 3.6415 | 0.0013 | 1.1821 | 0.034 |
| Q1HPU3 | Troponin C 25D | 56.29 | 6 | 3.0792 | 0.008775 | 4.1704 | 0.0002 | 1.3544 | 0.019 |
| U5KGX9 | Fatty acid synthase | 7.95 | 4 | 3.02685 | 0.001424 | 3.1711 | 8E-05 | 1.0477 | 0.383 |
| Q7YTA9 | Pheromone gland-specific fatty-acyl reductase | 44.78 | 2 | 2.88509 | 0.00101 | 3.3937 | 3E-06 | 1.1763 | 0.011 |
| B5BRC5 | Orai1 alternative splice form A | 17.1 | 2 | 2.6702 | 2.04E-05 | 2.4189 | 0.0017 | 0.9059 | 0.099 |
| Q3LBA0 | Chemosensory protein 1 | 60.98 | 11 | 2.65542 | 0.005369 | 3.2463 | 0.0003 | 1.2225 | 0.04 |
| Q17180 | Apolipophorin III | 61.29 | 18 | 2.59885 | 0.007674 | 3.4287 | 0.0003 | 1.3193 | 0.019 |
| E3UBU0 | Myosin light chain 2 | 51.23 | 1 | 2.58787 | 0.023989 | 3.4402 | 0.0003 | 1.3294 | 0.048 |
| I3VR84 | Aminopeptidase N-13 | 7.6 | 5 | 2.55604 | 0.015781 | 2.8144 | 0.0002 | 1.1011 | 0.359 |
| G6DFL5 | Serpin-27 | 0.98 | 1 | 2.46373 | 2.46E-05 | 2.8633 | 0.0014 | 1.1622 | 0.033 |
| Q1HPV6 | Hydroxysteroid dehydrogenase | 44.58 | 1 | 2.44719 | 0.011128 | 2.611 | 0.0019 | 1.0669 | 0.52 |
| G6CIY4 | Abc transporter | 12.4 | 2 | 2.38654 | 0.00364 | 2.7272 | 0.0003 | 1.1427 | 0.077 |
| C0LZ17 | Troponin I transcript variant C | 51.17 | 3 | 2.36586 | 0.010803 | 2.7216 | 0.0025 | 1.1504 | 0.2 |
| D4AH61 | Tan | 34.18 | 13 | 2.32642 | 0.003006 | 1.8619 | 8E-05 | 0.8003 | 0.017 |
| S4PFB9 | Interferon-related developmental regulator 1 | 5.14 | 1 | 2.27567 | 0.00108 | 1.7492 | 0.0088 | 0.7686 | 0.009 |
| I4DIK1 | Z band alternatively spliced PDZ-motif protein 66 | 25.99 | 1 | 2.25294 | 0.00046 | 2.9651 | 0.0001 | 1.3161 | 8E-04 |
| B6SDB6 | FAR-like protein VI (Fragment) | 4.71 | 1 | 2.21086 | 0.179041 | 1.8498 | 0.004 | 0.8367 | 0.542 |
| G6DCG7 | Putative four and a half lim domains-containing protein | 33.16 | 1 | 2.18902 | 0.00783 | 2.3425 | 0.0024 | 1.0701 | 0.443 |
| B2DBI1 | Muscle myosin heavy chain | 51.3 | 2 | 2.18165 | 0.001033 | 2.0943 | 0.0021 | 0.96 | 0.475 |
| Q2F5V0 | Troponin T | 44.79 | 28 | 2.16773 | 0.008383 | 2.6584 | 0.0002 | 1.2264 | 0.035 |
| Q5R1P5 | Heat shock protein hsp21.4 | 60.96 | 1 | 2.1589 | 0.042816 | 2.4634 | 0.0625 | 1.141 | 0.552 |
| S4PTF3 | Short-chain dehydrogenase/reductase family 16C member 6 | 14.13 | 2 | 2.15827 | 0.011888 | 2.5912 | 0.0177 | 1.2006 | 0.231 |
| G6CLJ9 | H+ transporting ATP synthase O subunit isoform 1 | 21.78 | 1 | 2.15613 | 0.028295 | 3.314 | 0.0309 | 1.537 | 0.088 |
| S4NRY1 | Tropomyosin 1 | 27.5 | 2 | 2.15332 | 0.000337 | 2.6599 | 0.0016 | 1.2352 | 0.016 |
| K4PWX1 | Arginine kinase isoform 2 | 66.23 | 15 | 2.14388 | 0.006507 | 2.5427 | 0.0011 | 1.186 | 0.068 |
| I3PN84 | Fatty acid reductase | 3.97 | 1 | 2.12282 | 0.005404 | 1.7778 | 0.0013 | 0.8375 | 0.054 |
| E5L8C3 | Cytochrome P450 | 5.09 | 1 | 2.10863 | 0.016541 | 1.5871 | 0.1274 | 0.7527 | 0.13 |
| C4P7H0 | Myosin heavy chain variant B | 62.2 | 2 | 2.08526 | 0.011255 | 1.5029 | 0.0003 | 0.7207 | 0.018 |
| Q5VCG3 | Arginine kinase | 38.82 | 1 | 2.06676 | 0.052315 | 2.3146 | 0.0093 | 1.1199 | 0.469 |
| S5M6F6 | Actin-4 | 67.82 | 1 | 2.06613 | 0.080181 | 3.06 | 0.0015 | 1.481 | 0.046 |
| H9ITU2 | Citrate synthase | 54.62 | 7 | 2.06486 | 0.004915 | 2.1085 | 0.0015 | 1.0211 | 0.761 |
| Q7YTA8 | Pheromone gland-specific fatty-acyl reductase | 43.7 | 2 | 2.02968 | 7.95E-05 | 2.0107 | 0.0011 | 0.9906 | 0.794 |
| Q2F665 | Perilipin | 29.22 | 8 | 1.99067 | 0.002002 | 1.0963 | 0.2213 | 0.5747 | 5E-04 |
| D4P239 | Elongation factor 1 alpha | 55.86 | 1 | 1.97801 | 0.194117 | 2.4223 | 0.0017 | 1.2246 | 0.375 |
| Q1HPK1 | Troponin C | 56.6 | 9 | 1.96833 | 0.000132 | 2.1838 | 0.0002 | 1.1095 | 0.012 |
| Q1HPQ0 | Tropomyosin-2 | 64.56 | 18 | 1.95372 | 0.000113 | 2.283 | 0.0008 | 1.1686 | 0.013 |
| Q1HPU0 | Tropomyosin-1 | 66.55 | 4 | 1.93989 | 0.000793 | 2.2486 | 0.0006 | 1.1592 | 0.02 |
| C0H6G6 | Putative cuticle protein | 32.85 | 1 | 1.92918 | 0.00674 | 2.6802 | 0.0008 | 1.3893 | 0.006 |
| S4PK30 | LIM domain-binding protein 3 | 35.71 | 2 | 1.92872 | 0.129299 | 2.2717 | 0.0088 | 1.1778 | 0.419 |
| S4P8C6 | Cytochrome c oxidase polypeptide IV | 14.37 | 1 | 1.90374 | 0.006192 | 1.7197 | 0.0077 | 0.9033 | 0.235 |
| Q1HPM4 | Muscular protein 20 | 77.17 | 9 | 1.89806 | 0.002609 | 2.2047 | 0.0002 | 1.1616 | 0.023 |
| I4DMJ0 | Limpet protein | 27.46 | 6 | 1.89728 | 4.41E-05 | 2.3177 | 0.0007 | 1.2216 | 0.005 |
| B8XE46 | Arginine kinase | 41.25 | 1 | 1.89653 | 0.027242 | 2.0581 | 0.0019 | 1.0852 | 0.423 |
| U5KFM5 | Acetyl-coA carboxylase | 3.13 | 2 | 1.89545 | 0.009561 | 1.8292 | 0.0039 | 0.965 | 0.663 |
| G6CWB9 | Putative acyl-coa dehydrogenase | 3.37 | 2 | 1.88529 | 0.119842 | 1.6301 | 0.0226 | 0.8646 | 0.491 |
| G6D9L1 | 1-acylglycerol-3-phosphate acyltransferase | 35.8 | 1 | 1.8806 | 8.72E-06 | 1.352 | 0.0025 | 0.7189 | 7E-05 |
| G6CLI1 | Putative ubiquitin conjugating enzyme 7 interacting protein | 5.39 | 1 | 1.8768 | 0.001268 | 2.6929 | 0.0181 | 1.4348 | 0.041 |
| G4XH88 | Myosin light chain | 48 | 2 | 1.8714 | 0.004256 | 1.941 | 0.0033 | 1.0372 | 0.594 |
| G1CJE3 | Protein Wnt | 5.26 | 1 | 1.85061 | 0.000816 | 2.0953 | 0.0002 | 1.1322 | 0.013 |
| H9J853 | Acid lipase-1 | 9.22 | 3 | 1.84133 | 0.002277 | 2.3627 | 0.0003 | 1.2832 | 0.003 |
| S5MNK0 | Actin-4 | 67.82 | 1 | 1.84061 | 0.006138 | 1.9739 | 1E-04 | 1.0724 | 0.239 |
| S4P9T5 | Heat Shock Protein 1 | 30.37 | 2 | 1.83143 | 0.023658 | 1.8127 | 0.0053 | 0.9898 | 0.913 |
| G6CSS4 | Short-chain dehydrogenease/reductase-like protein | 8.9 | 1 | 1.82904 | 0.284683 | 1.8896 | 4E-05 | 1.0331 | 0.908 |
| B2DBG2 | Sarcalumenin | 10.09 | 1 | 1.82321 | 0.0134 | 1.9881 | 0.0002 | 1.0905 | 0.258 |
| F8T9R1 | Isocitrate dehydrogenase | 35.59 | 1 | 1.81791 | 0.112061 | 2.0026 | 0.013 | 1.1016 | 0.593 |
| G6CZT2 | Tropomyosin-1 | 57.39 | 1 | 1.81556 | 0.059964 | 1.8595 | 0.0023 | 1.0242 | 0.854 |
| H9JJ13 | Malate synthase | 5.21 | 2 | 1.8152 | 0.027403 | 1.5849 | 0.0112 | 0.8732 | 0.261 |
| Q1HPN2 | Heat shock protein 1 ] | 62.12 | 9 | 1.80182 | 0.014301 | 1.8404 | 0.0031 | 1.0214 | 0.798 |
| B3VTP0 | Paramyosin | 69.02 | 2 | 1.80143 | 0.000387 | 2.0626 | 0.0003 | 1.145 | 0.008 |
| G6CV09 | Putative myosin light chain kinase | 11.39 | 1 | 1.79931 | 0.0232 | 1.9427 | 0.0028 | 1.0797 | 0.433 |
| Q9U557 | Cytosolic juvenile hormone binding protein 36 kDa subunit | 28.87 | 1 | 1.79662 | 0.014387 | 1.9234 | 0.0096 | 1.0706 | 0.496 |
| H9JU51 | Malate dehydrogenase | 58.06 | 14 | 1.78759 | 1.56E-05 | 2.0013 | 3E-05 | 1.1195 | 7E-04 |
| P07836 | Actin, muscle-type A1 | 72.07 | 7 | 1.78291 | 0.00058 | 1.9558 | 0.0007 | 1.097 | 0.053 |
| Q95UT2 | Iron regulatory protein 1 | 12.23 | 3 | 1.76633 | 0.057952 | 1.7091 | 0.0021 | 0.9676 | 0.795 |
| Q8T103 | BMKETTIN O | 35.8 | 1 | 1.76548 | 0.013531 | 1.7831 | 0.001 | 1.01 | 0.881 |
| B7U2V3 | Putative uncharacterized protein | 75.63 | 1 | 1.76169 | 0.001938 | 2.182 | 7E-05 | 1.2386 | 0.001 |
| B5M9A0 | Myosin heavy chain | 59.97 | 4 | 1.72906 | 0.000492 | 1.9802 | 0.0009 | 1.1453 | 0.016 |
| G6DIY5 | 26S proteasome non-ATPase regulatory subunit 14 | 33.12 | 1 | 1.72697 | 0.218787 | 1.6591 | 0.251 | 0.9607 | 0.85 |
| Q4ACU7 | Glutathione S-transferase | 38.92 | 1 | 1.71428 | 0.029261 | 2.0134 | 0.0098 | 1.1745 | 0.173 |
| L7QYC5 | Glucose-6-phosphate 1-dehydrogenase | 9.18 | 1 | 1.71021 | 0.091006 | 1.5436 | 0.0181 | 0.9026 | 0.516 |
| S4NHC7 | EH domain-containing protein 1 | 33.63 | 1 | 1.70861 | 0.005897 | 1.9147 | 0.0002 | 1.1206 | 0.053 |
| I4DKP7 | Muscle LIM protein | 74.74 | 1 | 1.70777 | 0.001087 | 2.7587 | 0.0002 | 1.6154 | 1E-04 |
| A7Y0T5 | Kettin1 protein | 13.61 | 2 | 1.70668 | 0.000346 | 1.801 | 0.0024 | 1.0553 | 0.231 |
| F2YDQ4 | ATP-binding cassette transporter subfamily G | 15.67 | 9 | 1.70638 | 0.000865 | 1.6002 | 0.0058 | 0.9378 | 0.241 |
| M1KUJ8 | Putative enolase protein | 22.13 | 2 | 1.69944 | 0.002903 | 1.7951 | 0.0003 | 1.0563 | 0.228 |
| Q86PP4 | Glutathione S-transferase 1 | 17.12 | 1 | 1.69555 | 0.018228 | 1.8652 | 0.008 | 1.1 | 0.117 |
| S5Y9E8 | Elongation factor-1 alpha | 60.31 | 1 | 1.68705 | 0.0056 | 2.0409 | 0.0008 | 1.2098 | 0.016 |
| G6DJA3 | Putative DNA ligase IV | 0.54 | 1 | 1.68387 | 0.000868 | 1.7884 | 0.005 | 1.0621 | 0.309 |
| C7AQP4 | Sarco/endoplasmic reticulum calcium ATPase | 43.12 | 43 | 1.67984 | 0.000168 | 2.0771 | 2E-05 | 1.2365 | 1E-04 |
| S5ZYM5 | Arginine kinase | 47.45 | 1 | 1.67861 | 0.040675 | 1.9751 | 0.002 | 1.1766 | 0.137 |
| Q5VCE9 | Arginine kinase | 35.69 | 1 | 1.6772 | 0.031339 | 1.9095 | 0.003 | 1.1385 | 0.213 |
| S4PIY9 | Protein split ends | 5.03 | 1 | 1.67363 | 0.129475 | 1.7168 | 0.0881 | 1.0258 | 0.904 |
| Q6U6J0 | Juvenile hormone epoxide hydrolase | 22.78 | 1 | 1.66934 | 0.074744 | 1.3425 | 0.0948 | 0.8042 | 0.215 |
| F8T9Q3 | Isocitrate dehydrogenase | 31.78 | 1 | 1.66889 | 0.000696 | 1.6573 | 0.0061 | 0.9931 | 0.893 |
| Q4ZJ79 | Heat shock cognate 70 protein | 55.13 | 1 | 1.66457 | 0.000704 | 1.5379 | 0.0015 | 0.9239 | 0.01 |
| G9LPT8 | UDP-glycosyltransferase UGT39B1 | 34.04 | 20 | 1.66455 | 0.000592 | 2.1364 | 0.0002 | 1.2835 | 7E-04 |
| I4DQ13 | Similar to CG9399 | 10.43 | 1 | 1.66327 | 0.001234 | 1.7456 | 0.0003 | 1.0495 | 0.141 |
| O96647 | 60S ribosomal protein L10 | 55.71 | 1 | 1.66128 | 0.027409 | 1.5831 | 0.0137 | 0.9529 | 0.619 |
| A9XZ22 | Putative N-terminal acetyltransferase complex ARD1 subunit-like protein | 6.86 | 1 | 1.65863 | 0.001814 | 1.145 | 0.1183 | 0.6903 | 0.002 |
| F2YDQ6 | ATP-binding cassette transporter subfamily G member Bm3 | 10.34 | 4 | 1.65363 | 0.002289 | 1.625 | 4E-05 | 0.9827 | 0.604 |
| C0J8G3 | Serpin-14 | 17.29 | 6 | 1.64108 | 0.006806 | 1.8075 | 0.0006 | 1.1014 | 0.106 |
| C0H6G7 | Putative cuticle protein | 32.85 | 1 | 1.64045 | 0.010372 | 2.7202 | 0.0001 | 1.6582 | 3E-04 |
| H9JT44 | Uncharacterized protein | 3.08 | 1 | 1.64036 | 0.025062 | 1.5145 | 0.142 | 0.9233 | 0.586 |
| U3RD44 | Geranylgeranyl diphosphate synthase | 3.31 | 1 | 1.62122 | 0.015494 | 1.5154 | 0.017 | 0.9347 | 0.345 |
| G6CQ56 | Putative plasma membrane associated protein, S3-12-like protein | 2.95 | 2 | 1.61988 | 0.000409 | 1.7305 | 0.0028 | 1.0683 | 0.148 |
| Q1HPX8 | 6-phosphogluconate dehydrogenase | 65.63 | 24 | 1.61329 | 0.00644 | 2.5593 | 0.0011 | 1.5864 | 0.001 |
| C6KYM1 | Juvenile hormone binding protein | 26.46 | 5 | 1.6065 | 0.000861 | 0.8304 | 0.0038 | 0.5169 | 3E-05 |
| I4DRJ2 | Dihydrolipoamide succinyltransferase component of 2-oxoglutarate dehydrogenase | 17.9 | 1 | 1.6003 | 0.112422 | 1.4738 | 0.0658 | 0.921 | 0.583 |
| S4PGX9 | Alpha actinin | 46.99 | 5 | 1.5997 | 0.013206 | 1.9823 | 0.0003 | 1.2392 | 0.016 |
| H9JC99 | 3-hydroxyisobutyrate dehydrogenase | 20.5 | 1 | 1.59944 | 0.003625 | 1.9373 | 0.0028 | 1.2113 | 0.025 |
| Q1HQ61 | Cytochrome b5 | 10.16 | 2 | 1.59432 | 0.000629 | 1.7254 | 0.0015 | 1.0822 | 0.069 |
| G6D5R8 | Peroxiredoxin | 12.82 | 1 | 1.59062 | 0.009233 | 1.6921 | 0.0089 | 1.0638 | 0.428 |
| Q2F5T4 | ATP synthase | 44.44 | 1 | 1.58836 | 0.003758 | 1.8161 | 0.0001 | 1.1434 | 0.016 |
| H6VTQ2 | DnaJ-12 | 22.35 | 4 | 1.58828 | 0.039645 | 1.9994 | 0.0005 | 1.2588 | 0.042 |
| H9J910 | Integrin beta | 2.44 | 1 | 1.58258 | 0.000294 | 2.4406 | 1E-05 | 1.5422 | 1E-06 |
| Q008X1 | Putative defense protein | 36.26 | 4 | 1.58086 | 0.002984 | 2.4101 | 2E-05 | 1.5246 | 1E-04 |
| Q11212 | Actin (Fragment) | 73.78 | 1 | 1.57503 | 0.00188 | 1.6615 | 0.0026 | 1.0549 | 0.193 |
| G9JQU1 | Catalase | 17.22 | 1 | 1.56718 | 0.002791 | 1.6195 | 0.0016 | 1.0334 | 0.332 |
| S5ZQE0 | Arginine kinase | 38.82 | 1 | 1.55995 | 0.013984 | 1.7121 | 0.0013 | 1.0975 | 0.187 |
| H9JEW9 | Aminopeptidase N-8 | 27.05 | 24 | 1.55662 | 0.000764 | 1.756 | 3E-05 | 1.1281 | 0.004 |
| Q8T101 | Titin-like protein | 8.74 | 8 | 1.55631 | 0.016447 | 1.8038 | 0.0065 | 1.1591 | 0.121 |
| C0H6N3 | Putative cuticle protein | 62.34 | 11 | 1.55119 | 0.002497 | 2.4453 | 0.0011 | 1.5764 | 9E-04 |
| C0LZ15 | Troponin I transcript variant A | 47.8 | 4 | 1.55118 | 0.000878 | 1.6756 | 0.0013 | 1.0802 | 0.082 |
| Q1HPS0 | Myosin regulatory light chain 2 | 83.08 | 6 | 1.54719 | 0.06383 | 1.6198 | 0.0005 | 1.0469 | 0.651 |
| Q3HLZ8 | Actin (Fragment) | 58.6 | 2 | 1.54589 | 3.14E-05 | 1.7186 | 2E-05 | 1.1117 | 4E-04 |
| M1VTM4 | Cytochrome c oxidase subunit 2 | 19.3 | 2 | 1.54479 | 0.12039 | 1.7306 | 0.054 | 1.1203 | 0.218 |
| Q18NR4 | Cocoonase (Fragment) | 7.57 | 1 | 1.54428 | 0.122159 | 1.4464 | 0.1225 | 0.9366 | 0.495 |
| M4M821 | Mitochondrial ATP synthase subunit epsilon | 11.48 | 1 | 1.54424 | 0.118239 | 1.6884 | 0.0268 | 1.0934 | 0.526 |
| S4PAT4 | Phosphatase type 2c | 9.7 | 1 | 1.53946 | 0.003 | 1.5209 | 0.0017 | 0.988 | 0.72 |
| E5LAN2 | Phosphotriesterase-like protein | 6.86 | 2 | 1.53859 | 0.004747 | 1.2031 | 0.0409 | 0.7819 | 0.007 |
| I4DNF6 | Isocitrate dehydrogenase | 30 | 4 | 1.53482 | 0.018626 | 1.8262 | 0.0008 | 1.1899 | 0.043 |
| Q1HPR6 | NADH-ubiquinone oxidoreductase Fe-S protein 7 | 21.9 | 2 | 1.5318 | 0.003117 | 1.7824 | 0.0001 | 1.1636 | 0.004 |
| F5C3T7 | Protein phosphatase 2 regulatory subunit A alpha isoform | 20 | 1 | 1.53159 | 0.071938 | 1.6073 | 0.0429 | 1.0494 | 0.714 |
| H9JSP4 | ATP synthase subunit beta | 69.59 | 2 | 1.53153 | 0.001139 | 1.64 | 0.0046 | 1.0709 | 0.202 |
| C0L810 | Protein Wnt | 7.55 | 1 | 1.5243 | 0.600077 | 0.941 | 0.6498 | 0.6173 | 0.449 |
| Q9BLJ6 | BAG domain-containing protein Samui | 6.5 | 3 | 1.52137 | 0.011125 | 1.6795 | 0.0074 | 1.104 | 0.218 |
| B3VTP1 | Paramyosin | 69.02 | 2 | 1.51338 | 0.00241 | 1.6727 | 0.0025 | 1.1053 | 0.077 |
| I4DMH8 | Membrane steroid binding protein | 12.43 | 3 | 1.51154 | 0.001903 | 1.8838 | 0.0003 | 1.2463 | 0.002 |
| S4PE30 | Pantothenate kinase | 3.85 | 1 | 1.50528 | 0.005286 | 1.6633 | 0.0009 | 1.105 | 0.027 |
| G6CXS8 | Hemocyte-specific integrin alpha subunit 2 | 10.69 | 1 | 1.5033 | 0.036188 | 1.5603 | 0.0032 | 1.0379 | 0.647 |
| J7EJ70 | Beta actin | 61.35 | 1 | 1.49848 | 0.004533 | 1.565 | 0.0023 | 1.0444 | 0.353 |
| G6CQG9 | Putative Ccar1 protein | 0.56 | 1 | 1.49604 | 0.000168 | 1.4694 | 0.0006 | 0.9822 | 0.376 |
| G6DFC1 | Adenylosuccinate synthetase | 5.63 | 1 | 1.49221 | 0.003946 | 1.5082 | 0.0495 | 1.0107 | 0.896 |
| Q2F625 | BWK-1-like protein | 11.11 | 1 | 1.49185 | 0.08667 | 1.6236 | 0.0611 | 1.0883 | 0.584 |
| K9J9Z3 | Beta-tubulin | 43.4 | 1 | 1.49161 | 0.001491 | 1.6278 | 0.0012 | 1.0913 | 0.042 |
| A8TUB4 | Aldehyde oxidase 1 | 27.56 | 1 | 1.48874 | 0.042623 | 1.6879 | 0.0041 | 1.1338 | 0.193 |
| A4UU25 | Cytochrome P450 | 24.11 | 11 | 1.48793 | 0.001343 | 1.3247 | 0.0015 | 0.8903 | 0.003 |
| Q2F5T3 | ATP synthase subunit alpha | 60.76 | 32 | 1.48376 | 0.009845 | 1.733 | 0.0005 | 1.168 | 0.027 |
| H9B421 | Yellow-d | 16.59 | 5 | 1.48229 | 0.011502 | 1.3842 | 1E-04 | 0.9338 | 0.221 |
| B2Z6M7 | B-actin | 59.84 | 1 | 1.48129 | 0.014525 | 1.5184 | 0.0062 | 1.0251 | 0.59 |
| H9J314 | ATP synthase subunit beta | 28.6 | 2 | 1.48088 | 0.011209 | 1.5703 | 0.0159 | 1.0604 | 0.454 |
| G6CRY8 | Thioredoxin-like protein | 7.14 | 1 | 1.48037 | 0.03711 | 1.6897 | 0.0018 | 1.1414 | 0.141 |
| H9J1D2 | Aspartate aminotransferase | 36.32 | 10 | 1.47712 | 5.78E-05 | 1.6817 | 0.0001 | 1.1385 | 2E-04 |
| B6VFJ5 | NADH-ubiquinone oxidoreductase chain 6 | 5.11 | 1 | 1.47519 | 5.39E-05 | 1.716 | 0.0028 | 1.1632 | 0.017 |
| Q5CCJ5 | Ras oncogene | 34 | 6 | 1.47398 | 0.043631 | 1.5549 | 8E-05 | 1.0549 | 0.502 |
| C0H6Z0 | Putative cuticle protein | 28.93 | 9 | 1.4714 | 0.020653 | 1.5318 | 0.0017 | 1.041 | 0.524 |
| H9J441 | Glutamine synthetase | 7.43 | 3 | 1.46921 | 8.78E-06 | 1.7813 | 0.0009 | 1.2124 | 0.002 |
| H9JRM8 | UDP-glucose 6-dehydrogenase | 2.92 | 1 | 1.46821 | 0.233515 | 1.5846 | 0.0176 | 1.0793 | 0.674 |
| S4PIM7 | Muscular protein 20 | 33.7 | 1 | 1.46136 | 0.000443 | 1.6002 | 0.0126 | 1.095 | 0.18 |
| S4PXB3 | E3 SUMO-protein ligase RanBP2 | 13.85 | 1 | 1.46071 | 0.047192 | 1.2806 | 0.3313 | 0.8767 | 0.447 |
| I4DIU5 | ATP synthase subunit beta | 64.4 | 1 | 1.46004 | 0.011375 | 1.587 | 0.0005 | 1.0869 | 0.142 |
| S4PAG3 | Protein kinase C beta type | 14.53 | 2 | 1.45797 | 0.001609 | 1.7475 | 0.0004 | 1.1986 | 0.002 |
| G6DN69 | G protein alpha S subunit Gs1 | 8.44 | 1 | 1.45638 | 0.250364 | 1.2631 | 0.1927 | 0.8673 | 0.523 |
| Q2F681 | Isocitrate dehydrogenase | 66.18 | 14 | 1.45534 | 0.000699 | 1.6565 | 0.0006 | 1.1382 | 0.007 |
| H9J9Q3 | Malic enzyme | 24.6 | 12 | 1.45465 | 0.000593 | 1.6739 | 6E-05 | 1.1507 | 0.001 |
| C3UZ71 | Myofilin variant C | 43.36 | 1 | 1.45288 | 0.005648 | 1.5236 | 0.0028 | 1.0486 | 0.062 |
| S4PE14 | Prolyl endopeptidase | 2.69 | 1 | 1.45138 | 0.00093 | 1.6119 | 0.0007 | 1.1106 | 0.017 |
| B6V762 | Flightin | 25.32 | 3 | 1.44881 | 0.02777 | 1.5317 | 0.0034 | 1.0572 | 0.441 |
| S4NNK0 | Spectrin beta chain | 12.75 | 1 | 1.44505 | 0.037879 | 1.4385 | 0.0077 | 0.9955 | 0.955 |
| G6CXW6 | Putative hormone-sensitive lipase | 2.21 | 1 | 1.44183 | 0.011218 | 1.4841 | 0.0054 | 1.0293 | 0.531 |
| B3Y9F5 | Fatty acid transport protein | 33.67 | 11 | 1.4399 | 0.003211 | 1.6597 | 6E-05 | 1.1527 | 0.006 |
| O17506 | Xanthine dehydrogenase | 1.92 | 3 | 1.43596 | 0.03149 | 0.9812 | 0.6094 | 0.6833 | 0.007 |
| S4NRV6 | Vacuolar protein sorting-associated protein (Vps13) | 22.86 | 1 | 1.43548 | 0.107289 | 1.456 | 0.0284 | 1.0143 | 0.91 |
| Q8T102 | Titin-like protein | 11.21 | 10 | 1.43446 | 0.000832 | 1.4887 | 0.0001 | 1.0378 | 0.131 |
| G6DQJ3 | Putative gamma-glutamylcysteine synthetase | 3.09 | 1 | 1.43407 | 0.03667 | 1.666 | 0.0004 | 1.1618 | 0.077 |
| I4DRS9 | Mitochondrial processing peptidase alpha subunit | 11.73 | 2 | 1.43404 | 0.301507 | 1.1125 | 0.204 | 0.7757 | 0.307 |
| S5M0T7 | Actin-4 | 59.84 | 1 | 1.43111 | 0.056387 | 1.5848 | 0.003 | 1.1074 | 0.268 |
| A0FDR1 | Thioredoxin | 50 | 4 | 1.43023 | 0.008291 | 1.4867 | 0.0023 | 1.0395 | 0.438 |
| S4PEZ6 | Phosphatidylinositol transfer protein/retinal degeneration b protein | 22.43 | 7 | 1.43022 | 0.04565 | 1.737 | 0.0013 | 1.2145 | 0.049 |
| W0M134 | Isocitrate dehydrogenase (Fragment) | 28.72 | 1 | 1.42859 | 0.001246 | 1.4317 | 0.0599 | 1.0022 | 0.98 |
| B7TZA3 | Uridine diphosphate glucosyltransferase | 3.11 | 1 | 1.42693 | 0.211107 | 1.4366 | 0.0138 | 1.0068 | 0.967 |
| C0H6F9 | Putative cuticle protein | 55 | 11 | 1.42463 | 0.000437 | 1.6286 | 0.0002 | 1.1432 | 4E-04 |
| J9XLX3 | Malate dehydrogenase | 6.31 | 1 | 1.42028 | 0.3294 | 1.8199 | 0.0224 | 1.2813 | 0.264 |
| I4DS67 | Scheggia | 34.55 | 2 | 1.41845 | 0.000497 | 1.5449 | 0.0002 | 1.0891 | 0.003 |
| Q5VCF7 | Arginine kinase | 43.92 | 1 | 1.41785 | 0.147812 | 1.475 | 0.0622 | 1.0403 | 0.796 |
| Q5R1T8 | NAD-dependent deacetylase sirtuin 2 homolog | 6.72 | 2 | 1.41752 | 0.007159 | 1.4358 | 0.0023 | 1.0129 | 0.737 |
| Q9NL59 | Annexin | 73.07 | 1 | 1.41543 | 0.024113 | 1.4931 | 0.0126 | 1.0549 | 0.48 |
| B6A8I8 | Guanine nucleotide-binding protein subunit gamma | 31.67 | 1 | 1.41395 | 0.075301 | 1.3641 | 0.0026 | 0.9648 | 0.705 |
| Q2F640 | Ubiquinol-cytochrome c reductase core protein II | 48.05 | 16 | 1.41347 | 0.048423 | 1.4499 | 4E-05 | 1.0258 | 0.732 |
| H6WC44 | Plasma membrane calcium ATPase | 14.97 | 1 | 1.41203 | 0.074207 | 1.3922 | 0.0023 | 0.986 | 0.874 |
| C3UZ70 | Myofilin variant A | 55.07 | 7 | 1.41087 | 0.000283 | 1.6527 | 0.0016 | 1.1714 | 0.007 |
| Q2F5K6 | Receptor expression enhancing protein isoform 2 | 31.93 | 7 | 1.40987 | 0.000746 | 1.6043 | 0.0002 | 1.1379 | 0.003 |
| G6DKC7 | Filamin | 26.22 | 7 | 1.40976 | 0.00142 | 1.6552 | 8E-05 | 1.1741 | 9E-04 |
| S4PSM8 | Patj-like protein | 24.72 | 1 | 1.40876 | 0.017911 | 1.4149 | 0.0226 | 1.0043 | 0.942 |
| C0H6L9 | Putative cuticle protein | 26.32 | 3 | 1.40697 | 0.005143 | 1.5344 | 0.0005 | 1.0906 | 0.059 |
| B1AAB5 | CYP332A1 | 5.8 | 3 | 1.40555 | 0.024356 | 1.4131 | 0.0027 | 1.0054 | 0.928 |
| H9N4S7 | Ubiquitin conjugating enzyme | 16.82 | 1 | 1.40118 | 0.001975 | 1.6077 | 0.0026 | 1.1474 | 0.02 |
| Q2F677 | Muscle LIM protein | 36.84 | 8 | 1.39948 | 0.000133 | 1.4805 | 0.0009 | 1.0579 | 0.053 |
| S4PBD2 | Heat shock protein cognate 5 | 11.72 | 1 | 1.39795 | 0.173592 | 1.3413 | 0.0426 | 0.9595 | 0.77 |
| B2CN03 | Glyceraldehyde-3-phosphate dehydrogenase | 43.91 | 1 | 1.39719 | 0.033736 | 1.6326 | 0.0055 | 1.1685 | 0.074 |
| H9N4S8 | Alpha-tubulin | 76.67 | 1 | 1.39588 | 0.007226 | 1.474 | 0.0002 | 1.056 | 0.188 |
| Q2F5P8 | Malate dehydrogenase | 56.5 | 9 | 1.39572 | 0.052063 | 1.4526 | 0.0052 | 1.0408 | 0.629 |
| L0N4J3 | Cytochrome P450 | 7.75 | 1 | 1.39465 | 0.054267 | 1.4979 | 0.0142 | 1.0741 | 0.449 |
| C4P7H1 | Myosin heavy chain variant C | 67.65 | 1 | 1.3932 | 0.017445 | 1.457 | 0.0065 | 1.0458 | 0.48 |
| E7CIC2 | 14-3-3 zeta | 50.77 | 1 | 1.39224 | 0.366317 | 1.6708 | 0.0286 | 1.2001 | 0.35 |
| G3K804 | Malate dehydrogenase | 20.36 | 1 | 1.3894 | 0.053586 | 1.5992 | 0.0161 | 1.151 | 0.196 |
| Q1HPL2 | Phosphate transport protein | 41.9 | 13 | 1.38918 | 0.000556 | 1.4816 | 0.0006 | 1.0666 | 0.04 |
| G9LPW1 | UDP-glycosyltransferase UGT46A2 | 7.81 | 4 | 1.38711 | 0.003497 | 1.3409 | 0.0085 | 0.9667 | 0.452 |
| Q7YZB5 | Glycerophosphoryl diester phosphodiesterase | 6.18 | 2 | 1.38679 | 0.001501 | 1.0708 | 0.2852 | 0.7721 | 0.001 |
| I6R2H4 | Peroxisomal membrane protein | 4.17 | 1 | 1.38672 | 0.061794 | 1.2599 | 0.0164 | 0.9085 | 0.3 |
| S4PXJ9 | Aconitase | 10 | 1 | 1.38626 | 0.218807 | 1.3503 | 0.0627 | 0.974 | 0.87 |
| S4PF21 | E3 ubiquitin-protein ligase suppressor of deltex | 8.74 | 1 | 1.38537 | 0.028158 | 1.5969 | 0.0194 | 1.1527 | 0.103 |
| G6DF88 | Citrate synthase | 38.92 | 2 | 1.38437 | 0.018563 | 1.4035 | 0.0001 | 1.0138 | 0.779 |
| S4PVY0 | Spectrin beta chain | 21.79 | 1 | 1.38422 | 0.048696 | 1.2536 | 0.0275 | 0.9056 | 0.209 |
| H9J2I8 | Glycylpeptide N-tetradecanoyltransferase | 10 | 3 | 1.38381 | 0.067335 | 1.4116 | 0.0038 | 1.0201 | 0.817 |
| G6DEN0 | Putative WD repeat domain 75 | 1.03 | 1 | 1.38146 | 0.004825 | 1.3102 | 0.0099 | 0.9484 | 0.251 |
| Q1HPP3 | H+ transporting ATP synthase subunit d | 89.39 | 12 | 1.37907 | 0.015651 | 1.4905 | 0.0005 | 1.0808 | 0.155 |
| G6CVQ1 | Putative fibrillin 2 | 0.26 | 1 | 1.37807 | 0.185484 | 1.3641 | 0.0555 | 0.9899 | 0.946 |
| Q2F648 | Tetraspanin D | 30.93 | 4 | 1.37774 | 0.001207 | 1.4409 | 0.0037 | 1.0458 | 0.257 |
| E3UKL9 | Nipsnap | 21.15 | 1 | 1.3772 | 0.078627 | 1.411 | 0.0006 | 1.0245 | 0.78 |
| C9EJW5 | Glycerol-3-phosphate dehydrogenase | 25.1 | 13 | 1.37627 | 0.061691 | 1.3451 | 0.0106 | 0.9774 | 0.791 |
| H9IXG0 | ATPase ASNA1 homolog | 10.45 | 2 | 1.37619 | 0.001944 | 1.558 | 0.0016 | 1.1321 | 0.008 |
| E9LG57 | Glyceraldehyde-3-phosphate dehydrogenase | 28.51 | 1 | 1.37524 | 0.004915 | 1.592 | 0.0387 | 1.1576 | 0.169 |
| Q1HPS8 | Chloride intracellular channel isoform 1 | 43.08 | 2 | 1.37453 | 0.149678 | 1.3639 | 0.0458 | 0.9923 | 0.951 |
| D5MTP1 | Bm8 interacting protein 2-5 | 9.09 | 1 | 1.37428 | 0.01097 | 1.3042 | 0.0015 | 0.949 | 0.23 |
| Q1HPM6 | ATP synthase-coupling factor 6 | 47.17 | 4 | 1.37112 | 0.00164 | 1.6377 | 0.0018 | 1.1944 | 0.007 |
| Q5EPR3 | Elongation factor 1-alpha | 59.44 | 1 | 1.37082 | 0.419297 | 1.1591 | 0.0525 | 0.8456 | 0.529 |
| Q2F5I9 | Aldehyde dehydrogenase | 43.55 | 2 | 1.36854 | 0.000142 | 1.6887 | 0.0005 | 1.2339 | 6E-04 |
| P22922 | Antitrypsin | 63.78 | 1 | 1.366 | 0.51866 | 0.9998 | 0.9969 | 0.7319 | 0.4 |
| Q1HQ06 | H+ transporting ATP synthase subunit e | 21.18 | 2 | 1.36351 | 0.000128 | 1.5516 | 0.0105 | 1.138 | 0.065 |
| D2WJ81 | Enolase | 22.43 | 1 | 1.36313 | 0.004739 | 1.4926 | 0.002 | 1.095 | 0.069 |
| I4DPR9 | D-amino acid oxidase | 3.73 | 1 | 1.36233 | 0.498395 | 1.0894 | 0.1119 | 0.7996 | 0.498 |
| Q86PG2 | ADP/ATP translocase | 65.67 | 21 | 1.36188 | 0.00326 | 1.4895 | 0.0001 | 1.0937 | 0.022 |
| Q1HQ25 | H+ transporting ATP synthase subunit g | 47.47 | 5 | 1.36125 | 0.00103 | 1.6922 | 0.001 | 1.2431 | 0.001 |
| G6CQQ1 | Atypical protein kinase C | 1.45 | 1 | 1.35751 | 0.037996 | 1.4995 | 0.0005 | 1.1046 | 0.155 |
| H9IT19 | Succinyl-CoA:3-ketoacid-coenzyme A transferase | 31.78 | 10 | 1.35373 | 0.000163 | 1.6053 | 0.0001 | 1.1858 | 3E-04 |
| G6DR05 | Beta-tubulin | 22.18 | 1 | 1.35234 | 0.053653 | 1.4018 | 0.0122 | 1.0366 | 0.652 |
| G6D9B6 | Putative serine protease-like protein 2 | 13.18 | 1 | 1.35157 | 0.098479 | 1.2967 | 0.0569 | 0.9594 | 0.613 |
| L7QL97 | Glucose-6-phosphate isomerase | 8.56 | 1 | 1.34997 | 0.083064 | 1.3169 | 0.0145 | 0.9755 | 0.775 |
| D0FHT5 | Mitogen-activated protein kinase p38 | 26.67 | 1 | 1.34793 | 0.309882 | 1.6522 | 0.0028 | 1.2257 | 0.252 |
| Q8N0P2 | Heat shock cognate protein | 65.49 | 2 | 1.34754 | 0.038947 | 1.1911 | 0.0187 | 0.8839 | 0.126 |
| G6DPP5 | Ubiquitin-activating enzyme E1 | 9.08 | 1 | 1.34721 | 0.00123 | 1.361 | 0.0033 | 1.0102 | 0.744 |
| H9ITA1 | Sulfhydryl oxidase | 9.09 | 3 | 1.34558 | 0.014444 | 1.2344 | 0.0487 | 0.9174 | 0.222 |
| Q2F6A1 | Electron-transfer-flavoprotein beta polypeptide | 50.99 | 13 | 1.34536 | 0.000355 | 1.4366 | 0.0001 | 1.0678 | 0.008 |
| Q6VEC7 | Cytochrome b | 3.55 | 2 | 1.34368 | 0.146022 | 1.6422 | 0.001 | 1.2222 | 0.103 |
| B3GQU6 | 3-hydroxyisobutyrate dehydrogenase | 20.5 | 1 | 1.3422 | 0.040541 | 1.5632 | 0.0201 | 1.1646 | 0.058 |
| Q1HPX4 | ATP synthase subunit gamma | 46.96 | 12 | 1.34207 | 0.001155 | 1.4396 | 0.0011 | 1.0727 | 0.046 |
| Q9GU82 | Pheromone gland-specific acyl-CoA desaturase | 37.58 | 2 | 1.34062 | 0.000837 | 1.4634 | 0.0042 | 1.0916 | 0.064 |
| Q1HQ34 | H+ transporting ATP synthase delta subunit | 73.91 | 8 | 1.33627 | 0.01022 | 1.5205 | 0.0004 | 1.1379 | 0.021 |
| Q68AX4 | Glycerol-3-phosphate dehydrogenase-1 | 52.49 | 15 | 1.33612 | 0.027757 | 0.9225 | 0.0003 | 0.6904 | 0.003 |
| E0D4V6 | Phenylalanyl-tRNA synthetase alpha subunit | 6.31 | 2 | 1.33556 | 0.10815 | 1.2588 | 0.0085 | 0.9425 | 0.553 |
| S4NZC3 | Corkscrew | 5.88 | 1 | 1.33268 | 0.00265 | 1.2503 | 0.0731 | 0.9382 | 0.32 |
| A5A7A3 | Isopentenyl diphosphate isomerase | 6.29 | 1 | 1.33142 | 0.032705 | 1.4206 | 0.0181 | 1.067 | 0.394 |
| Q1HPX9 | 3-hydroxyacyl-CoA dehydrogenase O | 3.53 | 1 | 1.33111 | 0.006264 | 1.2699 | 0.0041 | 0.954 | 0.156 |
| G6D995 | Adducin | 2.6 | 2 | 1.33091 | 0.006588 | 1.4049 | 0.0028 | 1.0556 | 0.222 |
| S4NWJ4 | Charged multivesicular body protein 2a | 3.02 | 1 | 1.32955 | 0.045748 | 1.1993 | 0.2066 | 0.9021 | 0.146 |
| H9J473 | Transporter | 1.26 | 1 | 1.32891 | 0.057088 | 1.3688 | 0.0127 | 1.0301 | 0.63 |
| S4PBZ7 | Neurochondrin-like protein | 6.24 | 1 | 1.32795 | 0.430835 | 1.1723 | 0.4528 | 0.8828 | 0.65 |
| S4PY31 | Glutathione S-transferase zeta 1 | 6.54 | 2 | 1.32611 | 0.041617 | 1.3029 | 0.0672 | 0.9825 | 0.791 |
| H9IV82 | Cytochrome P450 | 31.47 | 15 | 1.32342 | 0.25619 | 0.7649 | 0.0037 | 0.578 | 0.036 |
| G6CIX5 | Putative ankyrin 2,3/unc44 | 11.04 | 8 | 1.32235 | 0.097294 | 1.1302 | 0.0008 | 0.8547 | 0.141 |
| Q1HQ98 | Cytochrome c oxidase polypeptide IV | 37.08 | 5 | 1.32214 | 0.041765 | 1.4874 | 0.0033 | 1.125 | 0.123 |
| E5CWJ4 | Elongation factor 1-alpha | 47.95 | 1 | 1.32149 | 0.036247 | 1.2015 | 0.0125 | 0.9092 | 0.154 |
| C0H6J8 | Putative cuticle protein | 11.25 | 1 | 1.32076 | 0.012879 | 1.5351 | 0.0088 | 1.1623 | 0.059 |
| F8V3L0 | Gamma-glutamyl transpeptidase | 4.25 | 1 | 1.31903 | 0.369751 | 1.8271 | 0.0412 | 1.3852 | 0.165 |
| A8D0B7 | 1-Cys peroxiredoxin | 13.9 | 3 | 1.31901 | 0.007134 | 1.3704 | 0.0001 | 1.039 | 0.255 |
| L7QM00 | Triosephosphate isomerase | 23.65 | 1 | 1.31831 | 0.072519 | 1.3999 | 0.0024 | 1.0619 | 0.443 |
| Q1PCB1 | Pyridoxal kinase | 2.35 | 1 | 1.31539 | 0.210102 | 1.5378 | 0.0099 | 1.1691 | 0.239 |
| E2EHN2 | Glyceraldehyde-3-phosphate dehydrogenase | 39.57 | 1 | 1.31412 | 0.067317 | 1.3963 | 0.0146 | 1.0626 | 0.476 |
| H9JJI3 | Proteasome subunit beta type | 36.86 | 6 | 1.31371 | 0.002117 | 1.3619 | 0.0111 | 1.0367 | 0.415 |
| Q1HPL8 | NADH dehydrogenase | 25.16 | 4 | 1.3133 | 0.02161 | 1.2439 | 0.0001 | 0.9471 | 0.272 |
| H9C8H1 | Mutant chemosensory protein 1 variant | 27.18 | 3 | 1.31301 | 0.005971 | 1.4633 | 0.0005 | 1.1145 | 0.019 |
| E9LFX3 | Elongation factor-1 alpha | 17.24 | 1 | 1.3126 | 0.001848 | 1.1178 | 0.057 | 0.8516 | 0.007 |
| H9JHY9 | Oxysterol-binding protein | 0.53 | 1 | 1.31138 | 0.020518 | 1.4399 | 0.0004 | 1.098 | 0.084 |
| D2WIX9 | Dopa decarboxylase | 11.11 | 1 | 1.31048 | 0.080178 | 1.1184 | 0.6179 | 0.8534 | 0.328 |
| J9V7I8 | Isocitrate dehydrogenase | 15.25 | 1 | 1.30976 | 0.313222 | 1.332 | 0.0536 | 1.017 | 0.918 |
| Q306T4 | Alpha-tubulin | 68.48 | 1 | 1.30869 | 0.035725 | 1.319 | 0.0168 | 1.0078 | 0.848 |
| Q1HPV1 | Ubiquinone biosynthesis protein COQ4 homolog | 6.56 | 1 | 1.30645 | 0.401527 | 1.0684 | 0.1823 | 0.8178 | 0.386 |
| H9JSX4 | E3 ubiquitin-protein ligase | 1.08 | 1 | 1.3057 | 0.293913 | 1.2991 | 0.0642 | 0.995 | 0.972 |
| C0STP6 | Sepiapterin reductase | 3.07 | 1 | 1.29856 | 0.010448 | 1.2155 | 0.0039 | 0.936 | 0.081 |
| S4PG37 | N-alpha-acetyltransferase 35 | 1.46 | 1 | 1.29815 | 0.187678 | 1.0606 | 0.3234 | 0.817 | 0.166 |
| Q86GF8 | Putative uncharacterized protein | 26.27 | 1 | 1.29776 | 0.383379 | 1.2941 | 0.0044 | 0.9972 | 0.988 |
| D2Y061 | Isocitrate dehydrogenase | 34.02 | 9 | 1.29592 | 0.00011 | 1.4749 | 0.0003 | 1.1381 | 8E-04 |
| Q9ME79 | ATP synthase protein 8 | 26.42 | 4 | 1.29335 | 0.255922 | 1.4902 | 0.0157 | 1.1522 | 0.334 |
| I4DPX4 | Swiprosin | 12.31 | 1 | 1.29276 | 0.002244 | 1.1029 | 0.0174 | 0.8532 | 0.002 |
| L7R3G6 | Putative enolase protein | 29.1 | 1 | 1.29231 | 0.001752 | 1.5218 | 0.0028 | 1.1776 | 0.009 |
| A0FGR6 | NADPH--cytochrome P450 reductase | 39.3 | 1 | 1.29169 | 0.023451 | 1.3395 | 2E-05 | 1.037 | 0.416 |
| A0FDQ6 | ATPase inhibitor-like protein | 50.48 | 9 | 1.29111 | 0.004513 | 1.5216 | 0.0008 | 1.1785 | 0.004 |
| R4ZHH2 | Na+/K+ ATPase alpha subunit | 17.72 | 1 | 1.28764 | 0.093043 | 1.3006 | 0.0216 | 1.0101 | 0.905 |
| G6CPW3 | Oxygen resistance protein 1 | 5.59 | 1 | 1.28632 | 0.06905 | 1.383 | 0.018 | 1.0752 | 0.391 |
| Q6TAC3 | Sui1 | 8.18 | 1 | 1.28558 | 0.26543 | 1.1484 | 0.0654 | 0.8933 | 0.453 |
| H9JLF0 | NADH dehydrogenase [ubiquinone] 1 subunit C2 | 18.92 | 2 | 1.28399 | 0.190302 | 1.2975 | 0.0183 | 1.0105 | 0.927 |
| S4NTD0 | Delta-1-pyrroline-5-carboxylate synthase | 2.71 | 1 | 1.28394 | 0.032999 | 1.6569 | 0.1776 | 1.2905 | 0.27 |
| G6CXV7 | Ribonuclease L inhibitor-like protein | 11.68 | 1 | 1.28387 | 0.105133 | 1.2968 | 0.0258 | 1.0101 | 0.885 |
| G6DQ32 | Mitochondrial prohibitin complex protein 2 | 45.15 | 1 | 1.2826 | 0.300453 | 1.0432 | 0.5188 | 0.8133 | 0.255 |
| G1CKC4 | Isocitrate dehydrogenase | 41.18 | 1 | 1.28256 | 0.159821 | 1.4499 | 0.0017 | 1.1305 | 0.241 |
| C0J8G2 | Serpin-13 | 6.45 | 2 | 1.28173 | 0.048207 | 1.3143 | 0.01 | 1.0254 | 0.581 |
| D1LAP2 | Glyceraldehyde-3-phosphate dehydrogenase | 44.78 | 2 | 1.27862 | 0.069357 | 1.1319 | 0.037 | 0.8853 | 0.131 |
| Q2F672 | NADH-ubiquinone oxidoreductase B8 subunit | 8.99 | 1 | 1.2783 | 0.032136 | 1.3784 | 0.0023 | 1.0783 | 0.194 |
| Q9U8L9 | Mitochondrial carrier homolog | 10.51 | 2 | 1.27783 | 0.14947 | 1.2732 | 0.0079 | 0.9963 | 0.969 |
| H9JCE9 | Lon protease homolog, mitochondrial | 9.38 | 4 | 1.27551 | 0.008458 | 1.3592 | 0.0016 | 1.0656 | 0.059 |
| R4HKY3 | Heat shock protein 70 | 17.95 | 2 | 1.27421 | 0.07766 | 1.2792 | 0.0002 | 1.0039 | 0.954 |
| S4PCV0 | Plasma membrane calcium ATPase | 13.62 | 1 | 1.2742 | 0.218044 | 1.425 | 0.0024 | 1.1184 | 0.344 |
| Q308N8 | Glutathione S-transferase | 34.72 | 1 | 1.2733 | 0.194331 | 1.4471 | 0.0103 | 1.1365 | 0.283 |
| S4PVZ2 | Putative sporozoite surface protein | 13.11 | 1 | 1.26904 | 0.239193 | 1.1012 | 0.0618 | 0.8678 | 0.31 |
| S4NRB6 | Protein FAM98A | 6.31 | 1 | 1.26782 | 0.404194 | 1.1686 | 0.1715 | 0.9217 | 0.658 |
| A5JTL8 | L(B002) | 37.34 | 14 | 1.26618 | 0.00908 | 1.4087 | 0.0012 | 1.1126 | 0.029 |
| H9J0M9 | Cytochrome b-c1 complex subunit Rieske | 57.2 | 14 | 1.26573 | 0.080489 | 1.3672 | 0.0299 | 1.0801 | 0.399 |
| E9NYX5 | 3-hydroxyacyl-CoA dehydrogenase | 46.77 | 3 | 1.26368 | 0.010398 | 1.3866 | 0.0035 | 1.0973 | 0.039 |
| G6CS59 | Sterol carrier protein 2/3-oxoacyl-CoA thiolase | 12.63 | 1 | 1.26368 | 0.010676 | 1.1102 | 0.0891 | 0.8786 | 0.008 |
| S4NNG2 | Perilipin-4 | 4.64 | 1 | 1.26335 | 0.017291 | 1.2679 | 0.0022 | 1.0036 | 0.909 |
| Q8MUB0 | Dihydrolipoyl dehydrogenase | 57.66 | 17 | 1.26332 | 0.003024 | 1.4404 | 0.0018 | 1.1402 | 0.011 |
| G6CJ88 | Pyruvate kinase | 13.22 | 1 | 1.2621 | 0.037367 | 1.4138 | 0.0104 | 1.1202 | 0.017 |
| Q1HPZ7 | 60S ribosomal protein L7/l12 | 12.29 | 1 | 1.26001 | 0.280841 | 1.0784 | 0.0969 | 0.8558 | 0.303 |
| Q1HPM7 | Mitochondrial aldehyde dehydrogenase | 35.28 | 15 | 1.25973 | 0.009033 | 1.1651 | 0.0018 | 0.9249 | 0.044 |
| S4PEG4 | Alpha-1,3/1,6-mannosyltransferase ALG2 | 5.65 | 1 | 1.25767 | 0.107437 | 1.2891 | 0.0091 | 1.025 | 0.738 |
| D5GUW0 | Triosephosphate isomerase | 34.85 | 1 | 1.25723 | 0.015689 | 1.2389 | 0.0085 | 0.9854 | 0.694 |
| I4DN83 | Similar to CG4239 | 4 | 1 | 1.2565 | 0.285426 | 1.4226 | 0.0179 | 1.1322 | 0.363 |
| G9JQ10 | SUMO-1 activating enzyme | 11.94 | 1 | 1.25558 | 0.005878 | 1.5647 | 0.001 | 1.2462 | 0.002 |
| Q5UAT0 | Ribosomal protein L10 | 55.71 | 1 | 1.25548 | 0.098658 | 1.0537 | 0.5914 | 0.8392 | 0.127 |
| S4PKI2 | Putative methylmalonate-semialdehyde dehydrogenase | 13.3 | 4 | 1.25491 | 0.084769 | 1.2549 | 0.049 | 1 | 1 |
| V5ND41 | DnaJ | 6.2 | 1 | 1.25476 | 0.047236 | 1.1681 | 0.0516 | 0.9309 | 0.292 |
| G6D312 | Glucose transporter | 2.53 | 1 | 1.2546 | 0.00564 | 1.5216 | 0.001 | 1.2128 | 0.002 |
| Q3LB97 | Chemosensory protein 8 | 30.65 | 4 | 1.25412 | 0.140091 | 1.067 | 0.1907 | 0.8508 | 0.144 |
| H9JJZ4 | Acyl carrier protein | 17.28 | 3 | 1.25355 | 0.004122 | 1.3545 | 0.0077 | 1.0805 | 0.105 |
| K7W491 | Phosphoglycerate mutase | 19.22 | 3 | 1.25341 | 0.00662 | 1.2648 | 0.0779 | 1.0091 | 0.895 |
| J9XH29 | Glyceraldhyde-3-phosphate dehydrogenase | 21.56 | 1 | 1.25257 | 0.053394 | 1.132 | 0.014 | 0.9038 | 0.142 |
| Q2F5J9 | Mitochondrial thioredoxin 2 | 30.2 | 3 | 1.25122 | 0.016729 | 1.4519 | 0.0037 | 1.1604 | 0.006 |
| Q9NCX6 | Elongation factor 1-alpha | 61.63 | 1 | 1.25002 | 0.083703 | 0.9458 | 0.7046 | 0.7566 | 0.055 |
| E5CWT6 | Histone H3 | 70.64 | 1 | 1.24906 | 0.456784 | 0.8597 | 0.5172 | 0.6883 | 0.217 |
| Q9GU89 | Prophenoloxidase-1 | 34.74 | 1 | 1.2488 | 0.006334 | 1.5065 | 0.0092 | 1.2063 | 0.019 |
| H9JPB4 | Ubiquitin carboxyl-terminal hydrolase | 6.71 | 3 | 1.24807 | 0.005429 | 1.2398 | 0.0015 | 0.9933 | 0.796 |
| I6P6E4 | Dynamin | 9.74 | 1 | 1.24648 | 0.046776 | 1.1703 | 0.0116 | 0.9389 | 0.293 |
| Q19P01 | F-actin bundling protein | 25.5 | 2 | 1.24567 | 0.002181 | 1.3282 | 0.0014 | 1.0663 | 0.009 |
| H9IUJ6 | Transaldolase | 43.07 | 12 | 1.24516 | 0.004302 | 1.4667 | 0.0002 | 1.1779 | 0.002 |
| Q2F673 | Vacuolar ATPase subunit C | 41.71 | 16 | 1.24511 | 0.000343 | 1.3079 | 1E-04 | 1.0504 | 0.007 |
| G6DFU6 | Putative cullin 3 | 2.24 | 1 | 1.245 | 0.125924 | 1.2299 | 0.0237 | 0.9879 | 0.866 |
| S4P647 | UPF0396 protein | 11.84 | 1 | 1.24364 | 0.560932 | 1.1796 | 0.0065 | 0.9485 | 0.836 |
| A5A7A1 | Phosphomevalonate kinase | 15.05 | 3 | 1.24296 | 0.026782 | 1.3192 | 0.0025 | 1.0613 | 0.177 |
| B3TEF1 | Putative enolase protein | 31.75 | 2 | 1.24181 | 0.375937 | 1.1765 | 0.0281 | 0.9474 | 0.74 |
| E1U7I4 | Phosphoglucomutase | 3.4 | 1 | 1.24154 | 0.006505 | 1.3715 | 0.001 | 1.1047 | 0.021 |
| S4PH37 | Alpha Spectrin | 34.96 | 4 | 1.24023 | 0.003221 | 1.2665 | 0.0004 | 1.0211 | 0.303 |
| S4PS74 | Ras-related GTP-binding protein 4b | 16.98 | 1 | 1.24011 | 0.204807 | 1.1694 | 0.0591 | 0.943 | 0.595 |
| G6D528 | Putative cxpwmw03 | 20.3 | 5 | 1.23975 | 0.004345 | 1.3101 | 0.006 | 1.0567 | 0.136 |
| Q2F5R3 | Glutaredoxin | 31.9 | 4 | 1.23941 | 0.004232 | 1.3292 | 0.0013 | 1.0724 | 0.048 |
| S4PXT4 | Phosphatidylinositol-binding clathrin assembly protein | 7.95 | 3 | 1.23751 | 0.035431 | 1.1567 | 9E-06 | 0.9347 | 0.184 |
| E3WCW9 | Fatty acid transport protein | 15.38 | 1 | 1.23749 | 0.00175 | 1.4347 | 0.0002 | 1.1593 | 6E-04 |
| L7R607 | Putative enolase protein | 21.69 | 1 | 1.2368 | 0.351466 | 1.396 | 0.0971 | 1.1287 | 0.491 |
| G6DBI7 | Putative kinesin family member 21A | 0.73 | 1 | 1.23666 | 0.221959 | 1.335 | 0.0707 | 1.0795 | 0.366 |
| S4PZN7 | Serine/threonine-protein kinase unc-51 | 12.73 | 1 | 1.23624 | 0.558545 | 1.2418 | 0.2666 | 1.0045 | 0.985 |
| Q09IV5 | Troponin C type IIIa-like protein | 9.8 | 1 | 1.23616 | 0.167452 | 1.825 | 0.0048 | 1.4763 | 0.011 |
| F5BYI5 | Annexin | 44.27 | 2 | 1.23534 | 0.010197 | 1.2056 | 0.0137 | 0.9759 | 0.521 |
| Q1HPK7 | SUMO-1 activating enzyme | 19.17 | 3 | 1.23462 | 0.098743 | 1.1721 | 0.0008 | 0.9493 | 0.463 |
| H6A2U2 | Translation elongation factor 1-alpha | 64.1 | 1 | 1.23449 | 0.475967 | 0.7045 | 0.0041 | 0.5707 | 0.077 |
| S4PSF1 | Phosphoglycerate kinase | 27.23 | 1 | 1.23413 | 0.000129 | 1.3628 | 0.0043 | 1.1042 | 0.023 |
| S4NUC2 | Stromal membrane-associated protein 1 | 5.26 | 1 | 1.23157 | 0.035564 | 1.5203 | 0.0526 | 1.2344 | 0.104 |
| G6DRV9 | Hippocalcin | 25.26 | 5 | 1.23152 | 0.011572 | 1.6125 | 7E-06 | 1.3094 | 3E-04 |
| B1AAB4 | CYP4L6 | 18.44 | 6 | 1.23116 | 0.022493 | 0.9909 | 0.7811 | 0.8049 | 0.006 |
| S4PNK2 | Putative synaptojanin | 22.78 | 1 | 1.23094 | 0.577055 | 1.1869 | 0.0828 | 0.9642 | 0.887 |
| A5A7A2 | Diphosphomevalonate decarboxylase | 11.03 | 4 | 1.2308 | 0.052322 | 1.1824 | 5E-05 | 0.9607 | 0.446 |
| I4DR40 | Dihydropteridine reductase | 25.64 | 1 | 1.23064 | 0.613796 | 1.0336 | 0.2008 | 0.8399 | 0.569 |
| Q1HPI3 | C14orf124 protein | 18.81 | 1 | 1.22922 | 0.322928 | 1.4117 | 0.0183 | 1.1485 | 0.331 |
| F8UF66 | Isocitrate dehydrogenase | 36 | 1 | 1.22813 | 0.598975 | 1.761 | 0.0731 | 1.4339 | 0.125 |
| Q27451 | Phenoloxidase subunit 1 | 36.79 | 4 | 1.2243 | 0.007716 | 1.3043 | 0.0018 | 1.0653 | 0.079 |
| Q8MP08 | Serine protease | 5.9 | 2 | 1.22265 | 0.131269 | 1.2096 | 0.016 | 0.9893 | 0.883 |
| S4P9R8 | Bancal | 3.41 | 1 | 1.22243 | 0.033078 | 1.1769 | 0.0571 | 0.9628 | 0.517 |
| D1GJ60 | Sterol carrier protein 2/3-oxoacyl-CoA thiolase | 10.65 | 1 | 1.22238 | 0.020101 | 0.8773 | 0.0108 | 0.7177 | 7E-04 |
| D8VD26 | Sterol carrier protein 2/3-oxoacyl-CoA thiolase | 18.66 | 2 | 1.22171 | 0.012372 | 1.0469 | 0.3443 | 0.8569 | 0.015 |
| I6VP81 | Adaptor protein complex-1 gamma subunit transcript b | 2.91 | 1 | 1.2202 | 0.165645 | 1.2138 | 0.099 | 0.9947 | 0.959 |
| G6DEE0 | Superoxide dismutase | 10.65 | 1 | 1.22012 | 0.268154 | 1.573 | 0.0069 | 1.2892 | 0.061 |
| B6VFJ0 | Cytochrome c oxidase subunit 3 | 5.34 | 2 | 1.22001 | 0.00758 | 1.4209 | 0.0004 | 1.1647 | 0.003 |
| E9JEG8 | ASK1 | 1.26 | 1 | 1.21999 | 0.513513 | 1.1309 | 0.4218 | 0.927 | 0.736 |
| L7QWI8 | 6-phosphogluconate dehydrogenase | 22.8 | 1 | 1.21989 | 0.262057 | 1.2831 | 0.006 | 1.0518 | 0.636 |
| S5M4H4 | Actin-4 | 67.29 | 1 | 1.21923 | 0.094836 | 1.3005 | 0.0061 | 1.0667 | 0.339 |
| G9JQP3 | Proteasome 54kD subunit | 21.38 | 3 | 1.21867 | 0.002354 | 1.2294 | 0.0074 | 1.0088 | 0.719 |
| S4PC80 | 26S protease regulatory subunit 4 | 34.4 | 10 | 1.2185 | 0.005281 | 1.1948 | 0.0169 | 0.9806 | 0.561 |
| A5A7A4 | Farnesyl diphosphate synthase | 6.79 | 3 | 1.21843 | 0.013146 | 1.1906 | 0.0162 | 0.9772 | 0.485 |
| B5A989 | Lethal(3)neo18 | 15.26 | 3 | 1.21809 | 0.063019 | 1.3417 | 0.0014 | 1.1015 | 0.104 |
| S4P635 | Low-density lipoprotein receptor (Ldl) | 2.17 | 1 | 1.21764 | 0.011735 | 1.134 | 0.2566 | 0.9313 | 0.354 |
| A4GR70 | Yellow2 | 15.03 | 6 | 1.21681 | 0.00452 | 1.479 | 0.0003 | 1.2155 | 6E-04 |
| S4PEW9 | ADP-ribosylation factor 6 | 9.71 | 1 | 1.21611 | 0.001416 | 1.2703 | 0.0057 | 1.0446 | 0.174 |
| Q8T0Z6 | Ubiquitin conjugating enzyme-like protein | 9.55 | 1 | 1.21581 | 0.044717 | 1.1522 | 0.0051 | 0.9477 | 0.292 |
| Q2F666 | Phytanoyl-CoA dioxygenase peroxisomal | 9.33 | 2 | 1.21535 | 0.185464 | 0.7138 | 0.0004 | 0.5873 | 0.007 |
| Q1HPS9 | Vacuolar ATP synthase subunit E | 42.92 | 10 | 1.2153 | 0.005434 | 1.2013 | 0.0113 | 0.9885 | 0.714 |
| D1LAJ1 | Glyceraldehyde-3-phosphate dehydrogenase | 36.09 | 1 | 1.21409 | 0.412762 | 1.2898 | 0.0994 | 1.0624 | 0.691 |
| K9M0T0 | RpL40 protein | 49.22 | 2 | 1.21246 | 0.049611 | 1.2329 | 0.002 | 1.0169 | 0.72 |
| A1YQ87 | Enolase | 55.2 | 10 | 1.21118 | 0.058345 | 1.486 | 0.0002 | 1.2269 | 0.009 |
| A0FDQ0 | Programmed cell death protein 5-like protein | 24.62 | 2 | 1.21027 | 0.031265 | 1.0402 | 0.6182 | 0.8594 | 0.069 |
| C7E8M5 | Elongation factor 1-alpha | 52.32 | 1 | 1.21013 | 0.172399 | 0.984 | 0.8521 | 0.8132 | 0.074 |
| Q5KT57 | Ras protein | 32.07 | 4 | 1.20764 | 0.155398 | 1.0985 | 0.0785 | 0.9096 | 0.288 |
| Q1HPH7 | GA18592-PA | 30.56 | 3 | 1.20754 | 0.026384 | 1.1742 | 0.0037 | 0.9724 | 0.462 |
| H9IZ23 | Pyruvate kinase | 36.44 | 12 | 1.20655 | 0.011563 | 1.2337 | 0.0047 | 1.0225 | 0.507 |
| C6L8Q2 | Putative acetyl transferase | 64.65 | 18 | 1.20624 | 0.013541 | 1.3624 | 0.0005 | 1.1295 | 0.011 |
| H9JXM7 | Citrate synthase | 19.83 | 3 | 1.20611 | 0.022909 | 1.3315 | 0.0132 | 1.104 | 0.083 |
| H9J7L9 | Cytochrome c oxidase subunit 6A, | 40 | 3 | 1.20509 | 0.040562 | 1.3349 | 0.006 | 1.1077 | 0.089 |
| B7SJR4 | Protein kinase C | 3.44 | 1 | 1.20453 | 0.005134 | 1.4653 | 0.0012 | 1.2165 | 0.002 |
| S5M0X5 | Actin-4 | 67.82 | 1 | 1.20383 | 0.326467 | 1.7222 | 0.0408 | 1.4306 | 0.051 |
| I1XB05 | Adenylate kinase 2 | 18.6 | 1 | 1.20373 | 0.020408 | 1.2961 | 0.0063 | 1.0768 | 0.114 |
| Q1HPX2 | H+ transporting ATP synthase O subunit isoform 2 | 50.25 | 2 | 1.20372 | 0.132415 | 1.3626 | 0.0006 | 1.132 | 0.114 |
| Q2F624 | Peptidyl-prolyl cis-trans isomerase | 8.63 | 1 | 1.20313 | 0.094234 | 1.3578 | 0.0063 | 1.1285 | 0.113 |
| D2KWB9 | Carboxyl/cholinesterase 4A | 3.67 | 2 | 1.20281 | 0.144322 | 1.0824 | 0.0397 | 0.8999 | 0.214 |
| P82205 | Superoxide dismutase [Cu-Zn] | 86.36 | 8 | 1.2026 | 0.021741 | 1.3297 | 0.0794 | 1.1057 | 0.288 |
| G1CK89 | Glyceraldehyde-3-phosphate dehydrogenase | 51.4 | 3 | 1.20113 | 0.038678 | 1.2724 | 0.004 | 1.0594 | 0.237 |
| Q1HQB0 | Thioredoxin | 15.68 | 3 | 1.20008 | 0.014551 | 1.1846 | 0.0005 | 0.9871 | 0.648 |
| S4PD59 | Serine/threonine-protein phosphatase | 30.98 | 2 | 1.19821 | 0.022097 | 1.1679 | 0.0034 | 0.9747 | 0.459 |
| D5LR56 | ATP synthase subunit a | 7.56 | 3 | 1.19755 | 0.430448 | 1.516 | 0.0015 | 1.2659 | 0.138 |
| S4PXP2 | Putative ATP-dependent RNA helicase DHX36 | 1.2 | 1 | 1.1975 | 0.004993 | 1.1948 | 0.0062 | 0.9978 | 0.917 |
| S4NNL3 | Coronin | 12.8 | 3 | 1.19731 | 0.002057 | 1.2582 | 0.0009 | 1.0509 | 0.044 |
| K0GM01 | Na+,K+ ATPase alpha-subunit 1 | 17.18 | 5 | 1.19606 | 0.02066 | 1.3031 | 0.0003 | 1.0895 | 0.041 |
| G6DR28 | Mitochondria-associated granulocyte macrophage CSF signaling molecule | 17.6 | 1 | 1.1941 | 0.300938 | 1.1273 | 0.1362 | 0.944 | 0.594 |
| S4NQL9 | Transmembrane protein | 7.63 | 1 | 1.19325 | 0.110118 | 1.3346 | 0.0057 | 1.1185 | 0.119 |
| Q0MW37 | Ubiquitin | 57.89 | 1 | 1.19205 | 0.212783 | 1.2048 | 0.0152 | 1.0107 | 0.903 |
| G6D2K8 | PolyQ domain-containing hypothetical conserved protein | 3.2 | 1 | 1.18964 | 0.084571 | 1.672 | 0.005 | 1.4054 | 0.002 |
| A1YM13 | Phosphoribosylaminoimidazole carboxylase | 27.37 | 9 | 1.18957 | 0.019912 | 1.0889 | 0.0262 | 0.9154 | 0.048 |
| Q2F632 | ADP-ribosylation factor-like protein | 20.32 | 3 | 1.18952 | 0.359424 | 1.3948 | 0.0013 | 1.1725 | 0.209 |
| G3K9G8 | Acetoacetyl-CoA thiolase | 29.43 | 1 | 1.18948 | 0.007062 | 1.249 | 0.0124 | 1.05 | 0.219 |
| G6CUP3 | Proteasome subunit beta type | 18.05 | 4 | 1.18821 | 0.00731 | 1.3438 | 0.0018 | 1.1309 | 0.009 |
| I7AIS7 | Elongation factor 1a | 66 | 1 | 1.18802 | 0.062428 | 1.212 | 0.0444 | 1.0202 | 0.724 |
| I4DKE8 | Oligomycin sensitivity-conferring protein | 22.12 | 1 | 1.18788 | 0.013734 | 1.3168 | 0.007 | 1.1085 | 0.032 |
| Q2F5N8 | Nuclear migration protein nudC | 15.95 | 4 | 1.18768 | 0.021013 | 1.2027 | 0.015 | 1.0126 | 0.73 |
| S4PID6 | Dynein heavy chain | 4.07 | 1 | 1.18755 | 0.105562 | 1.2505 | 0.0156 | 1.053 | 0.286 |
| H9ITV0 | Glutamine synthetase | 18.72 | 6 | 1.1875 | 0.001109 | 1.2577 | 0.01 | 1.0591 | 0.112 |
| S4PY84 | Effete | 28.57 | 3 | 1.18673 | 0.018569 | 1.2181 | 0.0727 | 1.0264 | 0.657 |
| Q09GP9 | Putative GTP cyclohydrolase 1 type 2 | 8.86 | 2 | 1.1861 | 0.125711 | 1.3195 | 0.0073 | 1.1125 | 0.097 |
| Q2F645 | Transketolase | 59.81 | 17 | 1.18574 | 0.077015 | 1.1137 | 0.0857 | 0.9393 | 0.326 |
| H9JGA7 | Mitochondrial genome maintenance exonuclease 1 | 9.9 | 2 | 1.18558 | 0.112562 | 1.2816 | 0.0167 | 1.081 | 0.267 |
| Q2F5R4 | Glutamate dehydrogenase | 52.71 | 25 | 1.18534 | 0.004243 | 1.3415 | 0.0007 | 1.1317 | 0.003 |
| H9J5N5 | DNA polymerase | 0.91 | 1 | 1.18452 | 0.001329 | 1.1497 | 0.1295 | 0.9706 | 0.576 |
| E9KFH7 | Glu-+ pro-tRNA synthetase | 31.34 | 1 | 1.18401 | 0.026001 | 1.2171 | 0.0018 | 1.028 | 0.428 |
| L7R2G7 | Putative enolase protein | 19.58 | 1 | 1.18282 | 0.058618 | 1.3524 | 0.0002 | 1.1434 | 0.026 |
| G6DD05 | Tyrosine phosphatase IVA1 | 22.81 | 1 | 1.18237 | 0.121923 | 1.2698 | 0.0029 | 1.074 | 0.27 |
| Q6RJ00 | Cytochrome c oxidase subunit 2 | 10.67 | 1 | 1.18173 | 0.019074 | 1.2774 | 0.0032 | 1.081 | 0.048 |
| D2KMR4 | Putative lipase | 33.64 | 5 | 1.27777 | 0.009603 | 1.2661 | 0.0731 | 0.9909 | 0.876 |
| Q2F5J1 | 26S proteasome non-ATPase regulatory subunit 13 | 36.88 | 10 | 1.18 | 0.000173 | 1.1721 | 0.0004 | 0.9933 | 0.451 |
| Q1HQC6 | 26S protease regulatory subunit 6B | 35.18 | 11 | 1.17991 | 0.230334 | 1.1833 | 0.1219 | 1.0029 | 0.978 |
| H9JI85 | Proteasome subunit alpha type | 52.94 | 12 | 1.17922 | 0.00026 | 1.2837 | 0.0023 | 1.0886 | 0.01 |
| A0FDR2 | Putative uncharacterized protein | 15 | 2 | 1.17903 | 0.284048 | 1.3582 | 0.0002 | 1.152 | 0.168 |
| A9QUN2 | HnRNPA/B-like protein | 33.2 | 3 | 1.17764 | 0.195417 | 1.1513 | 0.0027 | 0.9777 | 0.769 |
| Q27452 | Phenoloxidase subunit 2 | 41.13 | 1 | 1.17575 | 0.020118 | 1.4135 | 0.0014 | 1.2022 | 9E-04 |
| G6DL76 | Fructose-bisphosphate aldolase | 38.05 | 1 | 1.17568 | 0.031352 | 1.1613 | 0.0199 | 0.9877 | 0.628 |
| G6CHV9 | Ubiquitin-conjugating enzyme E2L | 29.87 | 3 | 1.17563 | 0.005572 | 1.2442 | 0.0097 | 1.0583 | 0.139 |
| P41385 | Tubulin beta chain | 14.22 | 1 | 1.17423 | 0.241003 | 1.3171 | 0.0172 | 1.1217 | 0.246 |
| E2EIE5 | Isocitrate dehydrogenase | 31.78 | 1 | 1.17327 | 0.01365 | 1.2334 | 0.0007 | 1.0512 | 0.089 |
| Q60GK5 | Glutathione S-transferase delta | 91.67 | 9 | 1.17267 | 0.116723 | 1.0781 | 0.1081 | 0.9193 | 0.227 |
| S4NVE5 | Ankyrin repeat and FYVE domain-containing protein 1 | 8.62 | 1 | 1.17174 | 0.007282 | 1.2446 | 0.0035 | 1.0622 | 0.011 |
| Q1HPS1 | ML-domain containing secreted protein | 43.51 | 6 | 1.17073 | 0.001789 | 0.7847 | 0.0009 | 0.6703 | 7E-06 |
| Q1HQ18 | Proteasome beta subunit | 24.57 | 4 | 1.17064 | 0.016197 | 1.3723 | 0.0017 | 1.1723 | 0.002 |
| Q1HPP8 | Tropomyosin isoform 3 | 61.11 | 12 | 1.16843 | 0.02714 | 1.1857 | 7E-05 | 1.0148 | 0.623 |
| G0T414 | Alcohol dehydrogenase | 25.27 | 7 | 1.16838 | 0.016661 | 1.274 | 0.0143 | 1.0904 | 0.092 |
| G6CMQ8 | Putative vacuolar protein sorting 35 isoform 1 | 8.04 | 1 | 1.16811 | 0.257489 | 1.0072 | 0.8776 | 0.8622 | 0.156 |
| Q2QAJ8 | Sterol carrier protein x | 33.58 | 6 | 1.16803 | 0.002575 | 0.94 | 0.0815 | 0.8048 | 3E-04 |
| H9U5T8 | Catalase | 9.66 | 1 | 1.1677 | 0.011809 | 1.156 | 0.092 | 0.99 | 0.811 |
| I4DK96 | Similar to CG16787 | 5.61 | 2 | 1.16762 | 0.118553 | 1.2868 | 0.0054 | 1.1021 | 0.147 |
| F8UEV5 | Glyceraldehyde-3-phosphate dehydrogenase | 62.9 | 1 | 1.16563 | 0.047943 | 1.2136 | 0.0653 | 1.0412 | 0.376 |
| H9JKB2 | Methionine aminopeptidase 2 | 9.42 | 4 | 1.16536 | 0.00071 | 1.0732 | 0.0007 | 0.9209 | 4E-04 |
| H9J1E8 | Proteasome subunit beta type | 40 | 7 | 1.16434 | 0.138744 | 1.1977 | 0.001 | 1.0287 | 0.633 |
| I6XKQ0 | Heat shock protein 70-5 | 24.35 | 7 | 1.16388 | 0.007805 | 1.1607 | 8E-05 | 0.9972 | 0.882 |
| S4P912 | Selenium-binding protein 1 | 3.71 | 1 | 1.16338 | 0.06353 | 1.2845 | 0.0112 | 1.1041 | 0.108 |
| I4DJI9 | Cyclohex-1-ene-1-carboxyl-CoA hydratase | 35.35 | 2 | 1.16298 | 0.153008 | 1.2888 | 0.0037 | 1.1082 | 0.153 |
| O16143 | Rab1 protein | 64.36 | 9 | 1.16234 | 0.122482 | 1.0682 | 0.1773 | 0.919 | 0.226 |
| H9JFZ5 | Pyruvate carboxylase | 27.76 | 28 | 1.16229 | 0.000596 | 1.1997 | 0.0062 | 1.0322 | 0.183 |
| G6CR57 | Beta-tubulin | 15.21 | 1 | 1.1622 | 0.010089 | 1.133 | 0.0012 | 0.9749 | 0.273 |
| B0I1G6 | Glycerol kinase-3 | 2.87 | 1 | 1.16154 | 0.183772 | 1.1534 | 0.0165 | 0.993 | 0.92 |
| I4DRR4 | WD-repeat protein | 6.1 | 1 | 1.16134 | 0.338483 | 0.9821 | 0.842 | 0.8457 | 0.186 |
| B7SEP7 | Elongation factor 1-alpha | 50.13 | 1 | 1.16056 | 0.249024 | 1.0976 | 0.1452 | 0.9458 | 0.535 |
| I4DK20 | Proteasome subunit alpha type | 43.19 | 9 | 1.16018 | 0.037584 | 1.3532 | 0.0011 | 1.1664 | 0.003 |
| L7R1S9 | Putative enolase protein | 22.75 | 1 | 1.16001 | 0.002951 | 1.2773 | 0.0022 | 1.1011 | 0.006 |
| Q25516 | Methionine-rich storage protein 1 | 2.53 | 1 | 1.15952 | 0.008808 | 1.3677 | 0.064 | 1.1795 | 0.109 |
| H9JPA3 | DJ-1 beta | 42.63 | 8 | 1.15851 | 0.100707 | 1.24 | 0.0848 | 1.0703 | 0.407 |
| Q1HPT8 | U2 small nuclear ribonucleoprotein A' | 4.84 | 1 | 1.15775 | 0.455135 | 1.063 | 0.1623 | 0.9181 | 0.546 |
| M4MBL2 | Acyltransferase AGPAT2 | 17.04 | 5 | 1.15658 | 0.095358 | 1.4668 | 0.0271 | 1.2682 | 0.039 |
| H9J8X4 | Glucose-6-phosphate 1-dehydrogenase | 28.8 | 12 | 1.15658 | 0.015031 | 1.2191 | 0.0024 | 1.0541 | 0.067 |
| G6DDJ0 | 26S protease regulatory subunit 6A | 47.9 | 15 | 1.15625 | 3.69E-05 | 1.1502 | 0.016 | 0.9948 | 0.813 |
| I4DK99 | Kynurenine aminotransferase | 1.66 | 1 | 1.15566 | 0.507205 | 1.5536 | 0.007 | 1.3443 | 0.065 |
| D5MTP2 | Bm8 interacting protein 2-11 | 1.84 | 2 | 1.1554 | 0.221712 | 1.1924 | 0.0059 | 1.032 | 0.67 |
| Q2F5N7 | 26S proteasome non-ATPase regulatory subunit 14 | 36.98 | 2 | 1.15512 | 0.244735 | 1.1579 | 0.2208 | 1.0024 | 0.981 |
| S4P907 | Hepatoma-derived growth factor | 1.48 | 1 | 1.1549 | 0.138254 | 1.1475 | 0.0931 | 0.9936 | 0.917 |
| Q1HPX0 | Cytochrome c oxidase polypeptide Vb | 59.84 | 7 | 1.15409 | 0.034069 | 1.2726 | 0.0122 | 1.1027 | 0.077 |
| Q2F5X2 | Proteasome subunit alpha type | 21.69 | 5 | 1.15337 | 0.004519 | 1.2175 | 0.0275 | 1.0556 | 0.219 |
| C7ED93 | Heat shock protein 60 | 37.24 | 2 | 1.1531 | 0.267694 | 1.0787 | 0.1726 | 0.9355 | 0.467 |
| Q2F5P7 | Mitochondrial matrix protein p32 | 25.27 | 4 | 1.15307 | 0.073729 | 1.1941 | 0.0312 | 1.0356 | 0.499 |
| Q1HPP5 | Actin-depolymerizing factor 1 | 52.7 | 7 | 1.15208 | 7.53E-05 | 1.2299 | 4E-05 | 1.0675 | 2E-04 |
| G6CKF6 | Putative prolyl endopeptidase isoform 1 | 3.4 | 1 | 1.15144 | 0.012271 | 1.2364 | 0.0133 | 1.0738 | 0.081 |
| S4PD27 | Transcription initiation factor TFIID subunit 2 | 1.11 | 1 | 1.14951 | 0.399359 | 1.3152 | 0.0025 | 1.1441 | 0.235 |
| H9J567 | Proteasome subunit alpha type | 49.59 | 10 | 1.14932 | 0.008619 | 1.3394 | 0.0036 | 1.1654 | 0.006 |
| H9JKV0 | Proteasome assembly chaperone 2 | 17.55 | 3 | 1.14799 | 0.355297 | 0.9385 | 0.2451 | 0.8175 | 0.127 |
| C0L7N5 | Elongation factor 1-alpha | 41.69 | 1 | 1.14697 | 0.151588 | 0.9907 | 0.8205 | 0.8638 | 0.066 |
| E3VWK8 | Protein tyrosine phosphatase type IVA 1 | 31.65 | 1 | 1.14545 | 0.091538 | 1.1132 | 0.0459 | 0.9718 | 0.552 |
| Q1HQ44 | Proteasome subunit alpha type | 23.53 | 8 | 1.14293 | 0.00392 | 1.3691 | 0.0003 | 1.1979 | 2E-05 |
| G6D3N9 | Rab7 | 29.33 | 5 | 1.14245 | 0.237695 | 1.1356 | 0.0111 | 0.994 | 0.931 |
| S5M0Y3 | Actin-4 | 67.82 | 1 | 1.14221 | 0.518761 | 1.1368 | 0.1817 | 0.9952 | 0.972 |
| C0H6E4 | Putative cuticle protein | 6.38 | 1 | 1.14133 | 0.297368 | 0.5874 | 0.0113 | 0.5146 | 0.006 |
| Q2F5I6 | Glucose-6-phosphate isomerase | 15.47 | 4 | 1.14128 | 0.158322 | 1.1857 | 0.051 | 1.0389 | 0.531 |
| Q1HQ07 | 1110059p08rik-like protein | 8.04 | 3 | 1.14036 | 0.221663 | 1.1584 | 0.1497 | 1.0158 | 0.859 |
| S4PS71 | Annexin | 40.39 | 1 | 1.13977 | 0.030295 | 1.1038 | 0.3916 | 0.9684 | 0.689 |
| H9JJB7 | GrpE protein homolog | 23.18 | 3 | 1.13941 | 0.217297 | 1.055 | 0.7966 | 0.9259 | 0.638 |
| G6DE12 | Rotamase Pin1 | 4.49 | 1 | 1.13894 | 0.068758 | 1.1746 | 0.0183 | 1.0314 | 0.419 |
| Q2F662 | Proline synthetase co-transcribed bacterial-like protein | 23.66 | 5 | 1.13892 | 0.254701 | 1.1595 | 0.0806 | 1.0181 | 0.831 |
| Q1HQB6 | Ras-related GTP-binding protein Rab8 | 39.52 | 6 | 1.13846 | 0.129745 | 1.0503 | 0.5741 | 0.9226 | 0.336 |
| D2XZ04 | Guanine nucleotide-binding protein G(O) subunit alpha 1 isoform 1 | 10.73 | 1 | 1.13836 | 0.204358 | 1.2217 | 0.0035 | 1.0732 | 0.258 |
| Q1HQC7 | Aspartate aminotransferase | 39.21 | 14 | 1.13801 | 0.160107 | 1.3462 | 0.0017 | 1.183 | 0.029 |
| I4DK27 | Mitochondrial processing peptidase beta subunit | 12.85 | 1 | 1.13791 | 0.046376 | 1.267 | 0.0183 | 1.1135 | 0.078 |
| F2YDQ5 | ATP-binding cassette transporter subfamily B | 6.82 | 3 | 1.13763 | 0.107928 | 1.1669 | 0.2428 | 1.0257 | 0.784 |
| S4PL92 | Akt1 | 2.99 | 1 | 1.13697 | 0.209112 | 1.4071 | 0.0125 | 1.2376 | 0.035 |
| Q2F5P1 | N-acetyltransferase | 6.36 | 1 | 1.13626 | 0.641503 | 1.0921 | 0.3305 | 0.9611 | 0.838 |
| Q3ZJR2 | Elongation factor 1-alpha | 50.13 | 1 | 1.13559 | 0.337677 | 0.9726 | 0.6184 | 0.8565 | 0.139 |
| Q1HPJ9 | Vacuolar ATP synthase subunit H | 19.37 | 8 | 1.13511 | 0.042674 | 1.1572 | 0.0007 | 1.0195 | 0.514 |
| Q5CCL6 | P23-like protein | 14.02 | 2 | 1.1349 | 0.157916 | 1.2858 | 0.0246 | 1.133 | 0.016 |
| S5MNJ0 | Actin-4 | 67.82 | 1 | 1.13467 | 0.073431 | 1.1524 | 0.1936 | 1.0156 | 0.817 |
| C7AQZ3 | DnaJ-5 | 33.62 | 11 | 1.13433 | 0.415674 | 1.2396 | 0.0049 | 1.0928 | 0.405 |
| S4NIN4 | Collagen alpha-1(IV) chain | 2.26 | 1 | 1.13408 | 0.268815 | 1.1503 | 0.0197 | 1.0143 | 0.844 |
| Q2F5S7 | Coproporphirynogen oxidase | 5.88 | 2 | 1.13346 | 0.16561 | 1.212 | 0.022 | 1.0693 | 0.312 |
| E1XUQ3 | Thioredoxin | 23.58 | 1 | 1.13242 | 0.270084 | 1.1287 | 0.0849 | 0.9967 | 0.968 |
| I4DJY4 | 26S protease regulatory subunit | 39.3 | 12 | 1.13241 | 0.000615 | 1.1486 | 0.0004 | 1.0143 | 0.15 |
| G6DN87 | S-formylglutathione hydrolase | 12.06 | 1 | 1.13214 | 0.295029 | 1.11 | 0.132 | 0.9805 | 0.818 |
| G6CM39 | Serine/threonine-protein phosphatase | 31.71 | 3 | 1.13205 | 0.079081 | 1.1724 | 0.0162 | 1.0357 | 0.374 |
| I4DM38 | Beta-tubulin | 60.18 | 2 | 1.13192 | 0.278613 | 1.1101 | 0.2109 | 0.9807 | 0.828 |
| G6DQE9 | Putative coracle | 10.21 | 6 | 1.13179 | 0.023549 | 1.1919 | 0.0023 | 1.0532 | 0.099 |
| H9JTJ5 | Cytochrome b-c1 complex subunit 6 | 47.62 | 3 | 1.13172 | 0.111375 | 1.4864 | 0.0051 | 1.3134 | 0.006 |
| D2KQN9 | Serpin-4 | 14.25 | 5 | 1.13142 | 0.00328 | 1.2237 | 0.0005 | 1.0815 | 2E-04 |
| W5QMH3 | Fatty-acyl-CoA reductase | 4.97 | 1 | 1.13107 | 0.186837 | 1.0011 | 0.9906 | 0.8851 | 0.214 |
| B1NM55 | Elongation factor-1 alpha | 60.99 | 1 | 1.1309 | 0.073008 | 1.0845 | 0.4566 | 0.959 | 0.615 |
| H9JAG6 | Microtubule-associated protein | 13.49 | 3 | 1.13088 | 0.51798 | 1.8524 | 2E-05 | 1.638 | 0.007 |
| Q1HQ19 | Preimplantation protein | 4.09 | 1 | 1.13087 | 0.143953 | 1.8318 | 0.0432 | 1.6198 | 0.024 |
| G6DRK6 | Serine/threonine-protein phosphatase | 3.43 | 1 | 1.1291 | 0.5002 | 1.1529 | 0.0358 | 1.0211 | 0.868 |
| Q4H1F9 | Glutathione peroxidase | 40.7 | 5 | 1.12893 | 0.172994 | 1.2757 | 0.0196 | 1.13 | 0.105 |
| H9J2M1 | Importin subunit alpha | 23.06 | 6 | 1.12816 | 0.360832 | 1.0914 | 0.0587 | 0.9674 | 0.716 |
| Q2V0H5 | Transitional endoplasmic reticulum ATPase TER94 | 46.09 | 31 | 1.12721 | 0.083808 | 1.088 | 0.0023 | 0.9652 | 0.369 |
| P53359 | Guanine nucleotide-binding protein G(o) subunit alpha | 10.7 | 1 | 1.12693 | 0.08239 | 1.1987 | 0.0068 | 1.0637 | 0.114 |
| E5G3X5 | Death-associated LIM-only protein | 6.67 | 1 | 1.12525 | 0.206896 | 1.2785 | 0.0892 | 1.1362 | 0.195 |
| S0BCV4 | Apolipoprotein of lipid transfer particle-I/II | 3.28 | 6 | 1.12484 | 0.013583 | 1.3249 | 0.0014 | 1.1778 | 0.001 |
| B5LXT8 | Elongation factor 1-alpha | 47.3 | 1 | 1.12423 | 0.266417 | 1.0322 | 0.8105 | 0.9182 | 0.277 |
| Q1HQ99 | Vacuolar H+ ATP synthase 16 kDa proteolipid subunit | 11.61 | 1 | 1.12368 | 0.021553 | 1.4041 | 0.005 | 1.2495 | 0.004 |
| H9JEK2 | Delta-aminolevulinic acid dehydratase | 3.9 | 3 | 1.12279 | 0.270093 | 1.1298 | 0.0504 | 1.0062 | 0.928 |
| A9XXI1 | Putative 26S proteasome non-ATPase regulatory subunit 1 | 31.68 | 1 | 1.12189 | 0.567615 | 1.1448 | 0.1698 | 1.0205 | 0.887 |
| A7KCX9 | Heat shock protein hsp21.4 | 64.17 | 1 | 1.12183 | 0.79998 | 1.3623 | 0.5826 | 1.2144 | 0.475 |
| G6CWF0 | Peptidyl-prolyl cis-trans isomerase | 11.11 | 1 | 1.12167 | 0.152786 | 1.1355 | 0.0755 | 1.0123 | 0.787 |
| G6DDM6 | 3-hydroxyacyl-CoA dehydrogenase | 16.67 | 1 | 1.12117 | 0.376875 | 1.2548 | 0.0538 | 1.1192 | 0.292 |
| S4PFH0 | 2-hydroxy-3-oxopropionate reductase | 49.63 | 3 | 1.12097 | 0.088092 | 1.1107 | 0.1255 | 0.9908 | 0.862 |
| L7R3M8 | Putative enolase protein | 21.69 | 1 | 1.12033 | 0.619132 | 0.9342 | 0.4707 | 0.8338 | 0.304 |
| P82204 | Triosephosphate isomerase | 54.84 | 6 | 1.12032 | 0.018513 | 1.336 | 5E-05 | 1.1925 | 4E-04 |
| H9J615 | Protein-L-isoaspartate O-methyltransferase | 7.77 | 1 | 1.11997 | 0.364552 | 0.9598 | 0.4066 | 0.857 | 0.121 |
| E5EVW5 | Inorganic pyrophosphatase | 51.39 | 14 | 1.11987 | 0.071541 | 1.3483 | 6E-05 | 1.204 | 0.003 |
| G6D4R5 | Putative ATP-binding cassette sub-family B member 7 | 2.64 | 1 | 1.11726 | 0.401815 | 1.2137 | 0.061 | 1.0863 | 0.419 |
| Q1HPR4 | NADP-dependent oxidoreductase | 16.72 | 4 | 1.11706 | 0.034424 | 1.3462 | 0.0009 | 1.2052 | 8E-04 |
| Q5CCL2 | FK506-binding protein FKBP59 homologue | 7.1 | 3 | 1.11673 | 0.214836 | 1.1537 | 0.0011 | 1.0331 | 0.558 |
| B7SEP1 | Elongation factor 1-alpha | 48.43 | 2 | 1.11628 | 0.425709 | 1.0156 | 0.769 | 0.9098 | 0.387 |
| S4PXN1 | Transketolase | 13.36 | 1 | 1.11619 | 0.544671 | 0.9914 | 0.9419 | 0.8882 | 0.4 |
| I3NWV8 | Ryanodine receptor | 1.97 | 3 | 1.115 | 0.084727 | 1.1951 | 0.0029 | 1.0718 | 0.067 |
| Q1ET59 | Sorbitol dehydrogenase-2 | 16.76 | 5 | 1.11415 | 0.147701 | 1.0305 | 0.3004 | 0.925 | 0.166 |
| G6D091 | Muscle-specific protein 300 | 1.38 | 2 | 1.11213 | 0.24081 | 1.0258 | 0.5512 | 0.9224 | 0.238 |
| H9J1X9 | Adenylyl cyclase-associated protein | 55.06 | 4 | 1.11186 | 0.000906 | 1.2029 | 0.0047 | 1.0819 | 0.01 |
| U5KFM7 | Putative fatty acyl-CoA reductase | 7.45 | 1 | 1.11171 | 0.035232 | 1.0473 | 0.4118 | 0.9421 | 0.227 |
| Q1HPV9 | Glutathione transferase o1 | 32.92 | 7 | 1.11153 | 0.362743 | 1.1477 | 0.1118 | 1.0325 | 0.724 |
| A2TF14 | 6-phosphogluconolactonase | 9.87 | 2 | 1.11112 | 0.000231 | 1.2752 | 0.0079 | 1.1476 | 0.008 |
| Q2F5M8 | Phosphoserine aminotransferase | 18.68 | 5 | 1.11074 | 0.247993 | 1.3971 | 0.0076 | 1.2578 | 0.02 |
| L7R1H0 | Putative enolase protein | 24.34 | 1 | 1.10951 | 0.089344 | 1.2551 | 0.0032 | 1.1312 | 0.024 |
| E2EG90 | Elongation factor 1 alpha | 41.1 | 1 | 1.10939 | 0.685644 | 0.8279 | 0.0436 | 0.7463 | 0.214 |
| S4PXV8 | ATP-dependent Clp protease proteolytic subunit | 10.4 | 3 | 1.10864 | 0.162833 | 1.1753 | 0.0002 | 1.0602 | 0.217 |
| S4P6S5 | Leucine-rich repeat-containing protein 47 | 3.21 | 1 | 1.1085 | 0.211912 | 1.3093 | 0.0147 | 1.1811 | 0.042 |
| I4DPV2 | Mitochondrial import inner membrane translocase subunit tim44 | 5.18 | 1 | 1.10768 | 0.410166 | 1.0287 | 0.7575 | 0.9287 | 0.368 |
| S4PYI0 | Nop5 | 8.03 | 1 | 1.10728 | 0.5264 | 0.9937 | 0.8008 | 0.8974 | 0.388 |
| C0H6J3 | Putative cuticle protein | 20.28 | 2 | 1.10718 | 0.004835 | 1.1425 | 0.0065 | 1.0319 | 0.12 |
| I4DRV9 | Simila to CG15412 | 9.82 | 1 | 1.10703 | 0.262669 | 0.9957 | 0.9159 | 0.8994 | 0.122 |
| F2YHL5 | Robl | 10.53 | 1 | 1.10592 | 0.338791 | 1.1595 | 4E-05 | 1.0484 | 0.5 |
| Q1EPM0 | Glyceraldehyde-3-phosphate dehydrogenase | 49.1 | 2 | 1.10443 | 0.108135 | 1.232 | 0.0104 | 1.1155 | 0.052 |
| E2EJ91 | Malate dehydrogenase | 39.26 | 1 | 1.10371 | 0.129033 | 1.2134 | 0.0005 | 1.0994 | 0.051 |
| B2CMZ0 | Glyceraldehyde-3-phosphate dehydrogenase | 42.61 | 2 | 1.10321 | 0.375419 | 1.0887 | 0.1679 | 0.9868 | 0.848 |
| B7XFU6 | Clathrin heavy chain | 37.18 | 50 | 1.10254 | 0.077435 | 1.0248 | 0.4071 | 0.9295 | 0.097 |
| Q1HPU7 | Adenosylhomocysteinase | 23.95 | 10 | 1.10187 | 0.314808 | 1.2116 | 0.0048 | 1.0996 | 0.187 |
| A1E9B3 | Vacuolar ATP synthase catalytic subunit A | 53.97 | 1 | 1.101 | 0.017199 | 1.1281 | 0.0049 | 1.0246 | 0.163 |
| Q2F5R8 | Eukaryotic translation initiation factor 3 subunit E | 22.7 | 3 | 1.10084 | 0.363305 | 0.9103 | 0.1356 | 0.8269 | 0.077 |
| Q5CCJ4 | Glutathione S-transferase sigma | 69.12 | 5 | 1.10062 | 0.003783 | 1.0898 | 0.0436 | 0.9902 | 0.612 |
| G6CU56 | Putative 5-3 exoribonuclease 1 | 0.96 | 1 | 1.09966 | 0.411924 | 1.1251 | 0.25 | 1.0231 | 0.822 |
| Q2LAQ3 | Elongation factor 1-alpha | 33.58 | 1 | 1.09962 | 0.494175 | 0.9304 | 0.1154 | 0.8461 | 0.165 |
| H9JDV4 | Nucleoside diphosphate kinase | 67.32 | 8 | 1.09946 | 0.092854 | 1.1547 | 0.0081 | 1.0503 | 0.202 |
| H9CVB8 | Elongation factor 1 alpha | 29.36 | 1 | 1.09922 | 0.278682 | 1.0437 | 0.7186 | 0.9495 | 0.619 |
| S4P9G7 | Putative signal recognition particle 54 kDa protein | 21.56 | 1 | 1.0985 | 0.440149 | 0.9347 | 0.0882 | 0.8509 | 0.134 |
| H6TSI8 | Cytochrome c oxidase subunit 1 | 6.67 | 1 | 1.09697 | 0.500634 | 1.1167 | 0.2328 | 1.018 | 0.825 |
| A5A798 | 3-hydroxy-3-methylglutaryl-CoA synthase | 20.61 | 9 | 1.09623 | 0.068761 | 1.1246 | 0.0206 | 1.0259 | 0.449 |
| S4PV92 | Protein transport protein Sec24A | 10.67 | 2 | 1.09593 | 0.48277 | 0.9616 | 0.5198 | 0.8775 | 0.242 |
| I7EDT1 | ADP-ribosylation factor | 49.17 | 3 | 1.09503 | 0.010251 | 1.1314 | 0.0108 | 1.0332 | 0.137 |
| Q19P02 | Cytochrome b-c1 complex subunit 7 | 42.74 | 5 | 1.095 | 0.353574 | 1.1699 | 0.0064 | 1.0684 | 0.311 |
| Q1HQ04 | Transcription elongation factor B polypeptide 1 | 23.13 | 2 | 1.09499 | 0.137798 | 1.174 | 0.0517 | 1.0722 | 0.223 |
| G6CRJ2 | Putative UPF2 regulator of nonsense transcripts-like protein | 1.08 | 1 | 1.09416 | 0.643887 | 1.0813 | 0.1861 | 0.9883 | 0.934 |
| S4PDJ2 | Serine/threonine-protein kinase OSR1 | 5.33 | 1 | 1.09335 | 0.336859 | 1.127 | 0.1192 | 1.0308 | 0.688 |
| E9KFG2 | Glycogen synthase | 16.31 | 4 | 1.09296 | 0.145208 | 1.1159 | 0.163 | 1.021 | 0.603 |
| D5MTP5 | Bm8 interacting protein 2d-4 | 3.76 | 1 | 1.09281 | 0.357162 | 1.4976 | 0.0021 | 1.3704 | 0.005 |
| G6CMU9 | Putative kakapo | 3.67 | 2 | 1.09239 | 0.119825 | 1.1843 | 0.0491 | 1.0841 | 0.126 |
| Q0PVE3 | Copper chaperone | 38.89 | 2 | 1.09193 | 0.160136 | 1.1559 | 0.0591 | 1.0585 | 0.313 |
| E3WCW5 | Unclassified glutathione S-transferase | 42.13 | 7 | 1.09175 | 0.110919 | 1.1301 | 0.0074 | 1.0352 | 0.332 |
| S4PV72 | Tubulin beta-1 chain | 67.76 | 1 | 1.09165 | 0.23829 | 1.1037 | 0.1316 | 1.011 | 0.79 |
| C7ASM7 | Antitrypsin isoform 2 | 56.52 | 1 | 1.09156 | 0.033729 | 1.0646 | 0.2403 | 0.9753 | 0.491 |
| B6VAH6 | Proteasome subunit beta type | 18.93 | 5 | 1.09113 | 0.017173 | 1.0947 | 0.0223 | 1.0032 | 0.88 |
| Q1HQC4 | GTP binding protein | 21.16 | 7 | 1.09095 | 0.337559 | 0.9838 | 0.5051 | 0.9018 | 0.151 |
| Q2F5R9 | Eukaryotic translation initiation factor 1A | 21.38 | 3 | 1.09049 | 0.001594 | 1.1147 | 0.0009 | 1.0222 | 7E-04 |
| C0KYB6 | DDRGK domain-containing protein 1 | 14.88 | 3 | 1.08979 | 0.579914 | 1.1281 | 0.0159 | 1.0352 | 0.754 |
| D2CZY1 | Nuclear excision repair protein Rad23 | 17.59 | 6 | 1.08958 | 0.044816 | 1.1281 | 0.0004 | 1.0354 | 0.136 |
| S4P8E9 | Erythrocyte band 7 integral membrane protein | 7.83 | 1 | 1.08883 | 0.714732 | 0.9576 | 0.7634 | 0.8795 | 0.462 |
| Q1HPQ9 | Proteasome subunit beta type | 29.68 | 6 | 1.08865 | 0.03494 | 1.2078 | 0.0087 | 1.1094 | 0.021 |
| H9J2I0 | 6-phosphofructokinase | 2.83 | 3 | 1.08861 | 0.346547 | 1.1913 | 0.0259 | 1.0943 | 0.22 |
| Q8T7B0 | Dopa decarboxylase | 9.71 | 1 | 1.08804 | 0.627097 | 1.5358 | 0.0012 | 1.4115 | 0.026 |
| B7SEL5 | Elongation factor 1-alpha | 47.94 | 1 | 1.08747 | 0.064782 | 0.9385 | 0.2714 | 0.863 | 0.021 |
| H9JDT4 | Phosphoglycerate kinase | 34.75 | 3 | 1.08664 | 0.029436 | 1.2666 | 0.0039 | 1.1656 | 0.002 |
| C7AQY9 | DnaJ-1 | 21.08 | 7 | 1.08635 | 0.089717 | 0.9709 | 0.3131 | 0.8937 | 0.024 |
| I6P143 | Triosephosphate isomerase | 27.01 | 1 | 1.08579 | 0.625946 | 1.2084 | 0.1153 | 1.1129 | 0.326 |
| S4PZU9 | Rab11 | 41.75 | 9 | 1.08495 | 0.069104 | 1.1039 | 0.0654 | 1.0174 | 0.62 |
| Q1HQB8 | Peripheral-type benzodiazepine receptor | 13.02 | 3 | 1.08486 | 0.660603 | 1.1898 | 0.0193 | 1.0968 | 0.492 |
| B2CMB5 | Phosphorylase | 21.88 | 15 | 1.08468 | 0.091616 | 1.2071 | 0.0007 | 1.1129 | 0.012 |
| A1E131 | Kinesin-like protein 13 | 2.71 | 2 | 1.0844 | 0.197569 | 1.0169 | 0.8053 | 0.9377 | 0.29 |
| Q3HR36 | L-threonine dehydrogenase | 18.18 | 5 | 1.0842 | 0.145419 | 1.1232 | 0.0056 | 1.036 | 0.273 |
| G6DCY8 | Ras-related protein Rab-2A | 29.25 | 5 | 1.08416 | 0.143796 | 1.0572 | 0.1642 | 0.9751 | 0.534 |
| S4PE11 | Protein fat-free-like protein | 5.41 | 1 | 1.08379 | 0.23515 | 1.1106 | 0.3187 | 1.0247 | 0.766 |
| C0H6P2 | Putative cuticle protein | 14.06 | 1 | 1.08323 | 0.189438 | 1.019 | 0.6126 | 0.9407 | 0.11 |
| B9VTR3 | JAB-MPN domain protein | 1.72 | 1 | 1.08318 | 0.276994 | 1.0897 | 0.0939 | 1.006 | 0.907 |
| G6DPS6 | Putative 39S ribosomal protein L15, | 2.04 | 1 | 1.08113 | 0.058348 | 1.0234 | 0.3126 | 0.9466 | 0.08 |
| G6DD84 | ATPase n2b | 2.32 | 1 | 1.08051 | 0.572895 | 1.0516 | 0.1923 | 0.9733 | 0.79 |
| Q65Y02 | Superoxide dismutase | 31.48 | 5 | 1.08046 | 0.492448 | 1.3347 | 0.0043 | 1.2353 | 0.041 |
| Q8T9W9 | cGMP-dependent protein kinase | 4.47 | 2 | 1.07975 | 0.084978 | 1.088 | 0.157 | 1.0077 | 0.846 |
| G6DFA4 | Putative ATPase family AAA domain-containing protein 3 | 2.56 | 1 | 1.07955 | 0.377181 | 1.1536 | 0.0244 | 1.0685 | 0.298 |
| H9J6X0 | 4-hydroxyphenylpyruvate dioxygenase | 25.41 | 7 | 1.07913 | 0.070173 | 1.1211 | 0.062 | 1.0389 | 0.304 |
| Q1HQ55 | Peptidyl-prolyl cis-trans isomerase | 23.15 | 2 | 1.07832 | 0.047725 | 1.0871 | 0.2557 | 1.0082 | 0.873 |
| C7SIR9 | Heat shock protein 70 | 57.34 | 1 | 1.07796 | 0.373969 | 1.2445 | 0.0158 | 1.1545 | 0.072 |
| C8C300 | Glyceraldehyde-3-phosphate dehydrogenase | 42.61 | 1 | 1.07796 | 0.724487 | 1.2634 | 0.4395 | 1.1721 | 0.538 |
| Q56R81 | Double-time protein | 2.64 | 1 | 1.07662 | 0.126374 | 1.2262 | 0.02 | 1.1389 | 0.01 |
| S4PCB8 | Fatty acid transport protein | 23.78 | 1 | 1.07473 | 0.556775 | 1.0723 | 0.0217 | 0.9977 | 0.979 |
| S4PHL4 | Putative oxidoreductase GLYR1-like protein | 11.79 | 1 | 1.07463 | 0.206591 | 0.9703 | 0.6383 | 0.9029 | 0.141 |
| B5LXT6 | Elongation factor 1-alpha | 54.41 | 1 | 1.07446 | 0.067933 | 0.9502 | 0.1891 | 0.8844 | 0.005 |
| Q2F5Y9 | Mitochondrial aldehyde dehydrogenase | 10.66 | 4 | 1.07387 | 0.110002 | 1.1396 | 0.0274 | 1.0612 | 0.13 |
| Q1HQB1 | Salivary secreted ribonuclease | 28 | 2 | 1.07346 | 0.064202 | 1.1425 | 0.0274 | 1.0644 | 0.095 |
| S4P6I5 | LMBR1 domain-containing protein 2-like protein | 10.29 | 2 | 1.07342 | 0.216124 | 1.0278 | 0.4406 | 0.9575 | 0.281 |
| H9J4R0 | Ubiquitin carboxyl-terminal hydrolase | 1.06 | 1 | 1.07164 | 0.208366 | 1.0425 | 0.5038 | 0.9728 | 0.603 |
| Q2I0J5 | Glutathione S-transferase 3 | 17.73 | 3 | 1.07142 | 0.46014 | 0.9301 | 0.1317 | 0.8681 | 0.115 |
| H9IWL6 | Ferrochelatase | 1.82 | 1 | 1.07122 | 0.298444 | 1.0485 | 0.0871 | 0.9788 | 0.622 |
| G6DL70 | Putative Acidic fibroblast growth factor intracellular-binding protein | 10.28 | 1 | 1.07053 | 0.45798 | 1.0441 | 0.3722 | 0.9753 | 0.692 |
| G6DQG3 | Rer1 protein | 5.13 | 1 | 1.07039 | 0.816865 | 0.9592 | 0.5482 | 0.8961 | 0.641 |
| S5MIA3 | Actin-4 | 56.12 | 1 | 1.06969 | 0.388767 | 1.1297 | 0.0006 | 1.0561 | 0.327 |
| Q1G0S6 | Hemocyte-specific integrin alpha subunit 2 | 0.64 | 1 | 1.06966 | 0.174577 | 1.1561 | 0.0072 | 1.0808 | 0.052 |
| Q2F6C5 | Coiled-coil domain containing 25 protein | 15.46 | 2 | 1.06931 | 0.586361 | 1.0602 | 0.427 | 0.9915 | 0.927 |
| H9JBB0 | Probable cytosolic iron-sulfur protein assembly protein Ciao1 | 3.87 | 1 | 1.0693 | 0.043004 | 1.0776 | 0.008 | 1.0078 | 0.667 |
| Q2F693 | Glutamate receptor Gr1 | 2.22 | 1 | 1.06844 | 0.523329 | 1.2347 | 0.0606 | 1.1556 | 0.157 |
| H9J8L9 | Reticulon-like protein | 10.13 | 1 | 1.06776 | 0.288811 | 1.1517 | 0.0257 | 1.0786 | 0.155 |
| P31478 | V-type proton ATPase subunit F | 9.68 | 1 | 1.06754 | 0.572264 | 1.0907 | 0.1172 | 1.0217 | 0.805 |
| G6DE59 | ATP-dependent RNA helicase belle-like protein | 7.25 | 1 | 1.06619 | 0.419875 | 0.9854 | 0.544 | 0.9242 | 0.208 |
| G6D2S7 | Putative Spectrin beta chain | 18.76 | 21 | 1.06608 | 0.003792 | 1.1592 | 0.0004 | 1.0874 | 2E-04 |
| Q2HZG4 | Yellow-c | 30.47 | 1 | 1.06587 | 0.421151 | 1.2148 | 0.2427 | 1.1397 | 0.294 |
| G6CVH5 | Aspartyl-tRNA synthetase | 4.62 | 1 | 1.06475 | 0.144731 | 0.9953 | 0.8591 | 0.9347 | 0.06 |
| G6DRQ9 | Clip domain serine protease 4 | 2.49 | 1 | 1.06442 | 0.20533 | 1.0961 | 0.1325 | 1.0298 | 0.266 |
| Q2F5M0 | Ras-related protein 2 | 5 | 1 | 1.06398 | 0.07578 | 0.9423 | 0.1179 | 0.8856 | 0.008 |
| Q2F610 | DNA-damage inducible protein | 7.46 | 3 | 1.0638 | 0.302237 | 1.0708 | 0.1142 | 1.0066 | 0.888 |
| H9JY57 | Sulfhydryl oxidase | 7.1 | 1 | 1.06363 | 0.32383 | 1.0529 | 0.3835 | 0.9899 | 0.863 |
| S4PDV8 | Endoplasmic reticulum mannosyl-oligosaccharide 1,2-alpha-mannosidase | 2.76 | 1 | 1.0634 | 0.505328 | 1.081 | 0.1837 | 1.0166 | 0.776 |
| E9LFT6 | Elongation factor 1 alpha | 38.46 | 1 | 1.06319 | 0.151296 | 1.0069 | 0.7838 | 0.947 | 0.127 |
| Q2LAV0 | Elongation factor-1 alpha | 41.18 | 1 | 1.06303 | 0.802766 | 0.8138 | 0.1111 | 0.7656 | 0.26 |
| M1KUG4 | Carbamoylphosphate synthetase/aspartate transcarbamylase/dihydroorotase | 0.96 | 1 | 1.06251 | 0.195873 | 1.1081 | 0.0195 | 1.0429 | 0.119 |
| G4WE80 | Glyceraldehyde-3-phosphate dehydrogenase | 44.29 | 1 | 1.06108 | 0.69954 | 1.1448 | 0.1956 | 1.0789 | 0.541 |
| B8Y173 | Elongation factor 1-alpha | 64.71 | 1 | 1.06019 | 0.628689 | 0.9717 | 0.5731 | 0.9166 | 0.397 |
| Q1HPL3 | Proteasome 26S non-ATPase subunit 7 | 28.48 | 7 | 1.06016 | 0.188101 | 0.9856 | 0.6281 | 0.9297 | 0.028 |
| Q1HPS4 | Eukaryotic translation initiation factor 3 subunit K | 21.1 | 4 | 1.05995 | 0.763102 | 0.9938 | 0.844 | 0.9376 | 0.662 |
| S4P8T8 | Rho guanine dissociation factor | 15.67 | 3 | 1.05896 | 0.178404 | 1.1748 | 0.0038 | 1.1094 | 0.02 |
| Q0KIX8 | Glutamate synthase | 8.5 | 13 | 1.05831 | 0.337819 | 1.1094 | 0.0672 | 1.0483 | 0.363 |
| J4AF95 | Glyceraldehyde-3-phosphate dehydrogenase | 51.26 | 1 | 1.05733 | 0.141041 | 1.2099 | 0.0478 | 1.1443 | 0.05 |
| H9IXT4 | Alpha-tubulin N-acetyltransferase | 3.86 | 1 | 1.05725 | 0.013519 | 0.9434 | 0.5483 | 0.8923 | 0.156 |
| L7QTP0 | Glucose-6-phosphate 1-dehydrogenase | 7.73 | 1 | 1.05619 | 0.297438 | 1.0478 | 0.2857 | 0.9921 | 0.827 |
| G6CQL8 | AMP-activated protein kinase beta subunit | 3.93 | 1 | 1.0558 | 0.207393 | 1.2636 | 0.1708 | 1.1968 | 0.15 |
| B9U7C7 | Elongation factor 1-alpha | 56.48 | 1 | 1.05454 | 0.594476 | 0.8693 | 0.0199 | 0.8244 | 0.063 |
| G6CHT9 | Heterochromatin protein 1beta-like protein | 16.29 | 2 | 1.05426 | 0.396691 | 1.1723 | 0.0085 | 1.112 | 0.043 |
| S4PJR2 | Protein suppressor of forked | 3.55 | 1 | 1.05411 | 0.326862 | 1.0224 | 0.4689 | 0.97 | 0.467 |
| D6RVR8 | Ca2+/calmodulin-dependent protein kinase II | 13.73 | 5 | 1.0541 | 0.045102 | 0.8651 | 0.041 | 0.8207 | 0.004 |
| D2Y062 | Adenylate kinase 2 | 32.64 | 5 | 1.05399 | 0.055585 | 1.0787 | 0.0889 | 1.0235 | 0.406 |
| Q1HPY8 | Phosphatidylethanolamine binding protein isoform 1 | 52.97 | 5 | 1.05388 | 0.067181 | 1.2899 | 0.0027 | 1.224 | 5E-04 |
| W0I6G3 | DSX isoform F2 | 5.31 | 1 | 1.05371 | 0.540831 | 1.0674 | 0.3689 | 1.0129 | 0.866 |
| B9U6T6 | Carbamoylphosphate synthetase/aspartate transcarbamylase/dihydroorotase | 3.69 | 1 | 1.05333 | 0.69698 | 1.1226 | 0.249 | 1.0658 | 0.528 |
| Q2F5I1 | N-acetyl-glucosamine-6-phosphate isomerase | 4.87 | 1 | 1.05271 | 0.181619 | 1.1173 | 0.2826 | 1.0613 | 0.427 |
| Q2F5J2 | Prohibitin protein WPH | 54.74 | 12 | 1.05239 | 0.180463 | 1.1003 | 0.0592 | 1.0455 | 0.229 |
| M4TP13 | Elongation factor-1 alpha | 50.97 | 1 | 1.05106 | 0.550542 | 0.9691 | 0.6042 | 0.9221 | 0.265 |
| S4NMS9 | UPF0483 protein | 7.38 | 2 | 1.05103 | 0.073928 | 0.9494 | 0.5457 | 0.9033 | 0.162 |
| L7R3V7 | Glu-/pro-tRNA synthetase | 11.94 | 1 | 1.05039 | 0.843279 | 0.9985 | 0.987 | 0.9506 | 0.799 |
| C0H6N9 | Putative cuticle protein | 2.26 | 1 | 1.04917 | 0.754078 | 1.0306 | 0.5338 | 0.9823 | 0.876 |
| Q7Z1Z7 | Juvenile hormone binding protein | 4.94 | 1 | 1.04873 | 0.777095 | 1.1019 | 0.3891 | 1.0507 | 0.727 |
| Q9GSB6 | Heat shock protein hsp20.4 | 72.38 | 2 | 1.04864 | 0.483147 | 1.1468 | 0.0115 | 1.0936 | 0.128 |
| Q0ZB74 | Eukaryotic translation initiation factor 5 | 18.14 | 6 | 1.04853 | 0.696271 | 1.0954 | 0.0408 | 1.0447 | 0.627 |
| C0J8F8 | Serpin-7 | 20.05 | 5 | 1.04852 | 0.175581 | 0.9997 | 0.9964 | 0.9534 | 0.382 |
| A6P319 | HSP70 | 26.72 | 1 | 1.04851 | 0.633633 | 1.1303 | 0.0099 | 1.078 | 0.326 |
| A8R5V3 | Omega-class glutathione S-transferase | 10.16 | 2 | 1.04819 | 0.687151 | 1.206 | 0.0196 | 1.1506 | 0.157 |
| G1ERM8 | Histone 3 | 60.44 | 1 | 1.04712 | 0.676037 | 1.0822 | 0.3617 | 1.0335 | 0.741 |
| B7SEK4 | Elongation factor 1-alpha | 49.39 | 2 | 1.04642 | 0.854935 | 0.8371 | 0.1007 | 0.8 | 0.317 |
| I4DR57 | Eukaryotic translation initiation factor 3 subunit B | 10.82 | 5 | 1.04601 | 0.775588 | 0.8703 | 0.0244 | 0.8321 | 0.2 |
| S4P6J3 | Sorting nexin-12 | 16.67 | 1 | 1.04521 | 0.769919 | 0.9298 | 0.1453 | 0.8895 | 0.363 |
| Q2F5J7 | Ubiquitin conjugating enzyme isoform 2 | 14.88 | 2 | 1.04486 | 0.673717 | 1.0785 | 0.0597 | 1.0322 | 0.684 |
| G6DTG3 | RNA recognition motif protein split ends | 4.83 | 1 | 1.04465 | 0.730112 | 1.1258 | 0.0106 | 1.0777 | 0.419 |
| B6ZIV8 | Globin | 14.45 | 2 | 1.04343 | 0.077983 | 0.956 | 0.0091 | 0.9162 | 0.003 |
| I4DQ99 | Ubiquitin-activating enzyme E1c | 1.56 | 1 | 1.04291 | 0.61756 | 1.1267 | 0.0451 | 1.0803 | 0.269 |
| G6DGN3 | Prenyl-dependent CAAX metalloprotease | 4.31 | 1 | 1.04277 | 0.393598 | 0.9884 | 0.8242 | 0.9478 | 0.257 |
| K0C081 | Elongation factor 1 alpha | 59.2 | 1 | 1.04262 | 0.506793 | 1.0234 | 0.5662 | 0.9816 | 0.73 |
| Q5EPM4 | Elongation factor 1-alpha | 48.55 | 1 | 1.04211 | 0.246229 | 0.9328 | 0.3228 | 0.8951 | 0.077 |
| Q5MGE8 | Cysteine peptidase 2 cathepsin-B-like | 7.99 | 1 | 1.04133 | 0.323813 | 1.3693 | 0.1286 | 1.315 | 0.079 |
| J9Z496 | Serine/threonine-protein phosphatase | 30.1 | 8 | 1.04132 | 0.165962 | 1.089 | 0.0307 | 1.0458 | 0.078 |
| L0CNY1 | Glyceraldehyde-3-phosphate dehydrogenase | 22.97 | 1 | 1.04091 | 0.307189 | 1.082 | 0.4003 | 1.0395 | 0.564 |
| Q1HQ59 | Eukaryotic translation initiation factor 6 | 12.65 | 2 | 1.04083 | 0.596546 | 1.0534 | 0.4483 | 1.0121 | 0.816 |
| F4ZZ04 | Glyceraldehyde-3-phosphate dehydrogenase | 54.35 | 1 | 1.04043 | 0.783502 | 1.0714 | 0.5145 | 1.0298 | 0.821 |
| L7QW54 | Dopa decarboxylase | 10.57 | 1 | 1.04043 | 0.925963 | 1.0117 | 0.9539 | 0.9724 | 0.937 |
| S5M0T2 | Actin-4 | 65.96 | 2 | 1.04018 | 0.622031 | 1.121 | 0.0499 | 1.0777 | 0.254 |
| H9IX58 | Carboxylesterase ae27 | 9.66 | 3 | 1.04011 | 0.631705 | 1.2512 | 0.0691 | 1.2029 | 0.076 |
| Q9BLI6 | Chitinase-related protein 1 | 0.65 | 1 | 1.03983 | 0.887721 | 1.245 | 0.1726 | 1.1973 | 0.415 |
| E0D4V7 | Phenylalanyl-tRNA synthetase beta subunit | 11.15 | 7 | 1.03893 | 0.662668 | 1.0046 | 0.7525 | 0.9669 | 0.615 |
| A9Z0U8 | Microsomal cytochrome P450 | 3.39 | 1 | 1.03889 | 0.763589 | 1.0003 | 0.9955 | 0.9628 | 0.697 |
| D2Y5R5 | Aliphatic nitrilase | 18.93 | 5 | 1.03874 | 0.646881 | 0.9033 | 0.0499 | 0.8696 | 0.1 |
| J9XHD2 | Glyceraldhyde-3-phosphate dehydrogenase | 22.07 | 1 | 1.0381 | 0.646986 | 1.1468 | 0.0737 | 1.1048 | 0.1 |
| S4PDS9 | Heterogeneous nuclear ribonucleoprotein Q | 31.5 | 4 | 1.03797 | 0.430716 | 0.937 | 0.3435 | 0.9027 | 0.128 |
| S5TCX1 | Glyceraldhyde-3-phosphate dehydrogenase | 27.41 | 1 | 1.03778 | 0.326917 | 1.086 | 0.0563 | 1.0464 | 0.036 |
| J9V5U6 | Elongation factor 1 alpha | 31.12 | 2 | 1.03775 | 0.326027 | 0.9351 | 0.0347 | 0.9011 | 0.012 |
| S4PCM7 | Dynein heavy chain | 4.4 | 1 | 1.03679 | 0.360683 | 1.1318 | 0.0996 | 1.0917 | 0.087 |
| S4P9Z3 | Lysophospholipase-like protein 1 | 3.51 | 1 | 1.03502 | 0.506678 | 1.1655 | 0.0374 | 1.1261 | 0.018 |
| Q2F643 | TPR-repeat protein | 2.49 | 1 | 1.03375 | 0.676001 | 1.0845 | 0.3107 | 1.0491 | 0.462 |
| G6DEY4 | TNF receptor-associated factor 3 interacting protein 1 | 2.5 | 1 | 1.03356 | 0.926603 | 1.6344 | 0.211 | 1.5813 | 0.051 |
| S4PKC4 | Dihydropyrimidinase | 13.19 | 2 | 1.03345 | 0.785436 | 0.8863 | 0.0426 | 0.8576 | 0.181 |
| F8UN43 | Heat shock protein 68 | 20.7 | 1 | 1.03314 | 0.721584 | 1.1031 | 0.0025 | 1.0677 | 0.343 |
| Q76FN3 | G protein alpha q isoform 1 | 25.21 | 7 | 1.03276 | 0.135076 | 1.0775 | 0.0211 | 1.0433 | 0.06 |
| B2CPX9 | Glutathione S-transferase theta | 43.52 | 1 | 1.03248 | 0.700423 | 0.9338 | 0.4403 | 0.9044 | 0.282 |
| S4NXJ1 | Spliceosomal protein on the X | 2.49 | 1 | 1.03216 | 0.847309 | 0.977 | 0.8882 | 0.9465 | 0.749 |
| Q9U5M9 | Vacuolar ATPase subunit a | 11.89 | 5 | 1.03205 | 0.690132 | 1.0794 | 0.0748 | 1.0459 | 0.441 |
| D3W865 | Ribosomal protein S5 | 34.57 | 1 | 1.03131 | 0.683083 | 0.9529 | 0.0014 | 0.924 | 0.219 |
| C0H6N5 | Putative cuticle protein | 12.98 | 1 | 1.0311 | 0.737247 | 0.9968 | 0.9259 | 0.9667 | 0.637 |
| A9XZG5 | Putative syntaxin 1A | 8.92 | 1 | 1.03079 | 0.504489 | 1.3201 | 0.0169 | 1.2807 | 0.002 |
| H9JKS9 | Annexin | 75.62 | 3 | 1.03031 | 0.830584 | 1.1199 | 0.062 | 1.0869 | 0.437 |
| H9ITX1 | Catalase | 56.41 | 20 | 1.02971 | 0.362354 | 1.0209 | 0.0339 | 0.9914 | 0.703 |
| E3VLC1 | HOP | 2.51 | 1 | 1.02965 | 0.851871 | 1.2745 | 0.1925 | 1.2378 | 0.168 |
| T2AV85 | Glyceraldhyde-3-phosphate dehydrogenase | 52.07 | 1 | 1.02924 | 0.890528 | 1.1675 | 0.191 | 1.1344 | 0.398 |
| B9W1Z2 | Myofilin isoform B | 54.4 | 1 | 1.02878 | 0.883025 | 1.4174 | 0.0202 | 1.3777 | 0.066 |
| Q0ZB73 | Eukaryotic translation initiation factor 3 subunit J | 17.01 | 3 | 1.02793 | 0.729272 | 1.0744 | 0.1219 | 1.0452 | 0.497 |
| S4P3S2 | Zipper | 18.86 | 3 | 1.02773 | 0.388963 | 0.9279 | 0.006 | 0.9029 | 0.011 |
| I4DNE8 | Surfeit locus protein | 12.96 | 2 | 1.02757 | 0.628677 | 0.9668 | 0.3361 | 0.9408 | 0.159 |
| E2IV54 | V ATPase A | 36.07 | 1 | 1.0275 | 0.921466 | 1.0911 | 0.6791 | 1.0619 | 0.811 |
| C9E270 | DEAD box ATP-dependent RNA helicase-like protein | 10.22 | 2 | 1.02465 | 0.177058 | 0.8143 | 0.0374 | 0.7947 | 0.007 |
| S4NWX2 | Ras-related GTP binding protein | 10.19 | 1 | 1.02459 | 0.837161 | 0.8239 | 0.0329 | 0.8041 | 0.079 |
| H9JEN7 | NAD-dependent protein deacylase | 9.59 | 2 | 1.02446 | 0.678035 | 1.0959 | 0.09 | 1.0698 | 0.162 |
| Q3S8M2 | Beta-tubulin | 50.34 | 1 | 1.023 | 0.96468 | 0.888 | 0.8146 | 0.868 | 0.692 |
| I4DP94 | SkpA protein | 28.95 | 2 | 1.02299 | 0.714368 | 1.0381 | 0.6384 | 1.0148 | 0.844 |
| H9JXC9 | Vacuolar protein sorting-associated protein 35 | 17.85 | 8 | 1.02171 | 0.86723 | 0.983 | 0.7264 | 0.9621 | 0.713 |
| S4PCR5 | AP-3 complex subunit delta-1 | 6.2 | 1 | 1.0202 | 0.765359 | 1.0315 | 0.2656 | 1.0111 | 0.812 |
| G6CIS0 | Putative stromal antigen | 1.4 | 2 | 1.02003 | 0.856702 | 0.9484 | 0.3589 | 0.9298 | 0.453 |
| S4PX15 | COP9 signalosome complex subunit 2 | 6.12 | 2 | 1.01887 | 0.491203 | 0.9856 | 0.5535 | 0.9673 | 0.251 |
| Q6T3A7 | Thiol peroxiredoxin | 72.82 | 13 | 1.01847 | 0.789699 | 1.0683 | 0.1391 | 1.0489 | 0.379 |
| B9ZZR0 | Aquaporin AQP-Bom3 | 5.56 | 1 | 1.01764 | 0.489788 | 1.1821 | 0.012 | 1.1616 | 0.001 |
| G6DBA8 | Ribosomal protein L7A | 27.61 | 1 | 1.01697 | 0.6075 | 1.0043 | 0.8549 | 0.9875 | 0.651 |
| P98092 | Hemocytin | 9.64 | 20 | 1.01544 | 0.771466 | 0.8117 | 0.0023 | 0.7993 | 0.007 |
| Q1HPN7 | Fructose-bisphosphate aldolase | 64.56 | 10 | 1.01516 | 0.835461 | 1.1908 | 0.0027 | 1.1731 | 0.031 |
| E5EVW1 | Ubiquitin carboxy-terminal hydrolase CG4265 | 20.43 | 4 | 1.01473 | 0.791578 | 1.1161 | 0.0191 | 1.0999 | 0.083 |
| Q9NKX2 | Coatomer subunit gamma | 11.4 | 7 | 1.01439 | 0.708795 | 0.9175 | 0.2797 | 0.9044 | 0.137 |
| O44305 | Elongation factor 1-alpha | 50.85 | 1 | 1.01434 | 0.64356 | 0.8726 | 0.0005 | 0.8603 | 0.003 |
| S4PH57 | Poly U binding factor 68kD | 6.42 | 1 | 1.0126 | 0.362765 | 1.0277 | 0.3082 | 1.0149 | 0.457 |
| G6CIU7 | Putative BUB3 budding uninhibited by benzimidazoles 3 | 2.43 | 1 | 1.01253 | 0.82991 | 1.0762 | 0.5262 | 1.0629 | 0.484 |
| Q1HQ36 | Ubiquitin conjugating enzyme E2 | 22.52 | 2 | 1.01243 | 0.845315 | 1.077 | 0.0397 | 1.0638 | 0.242 |
| H9IUV5 | Inosine-5'-monophosphate dehydrogenase | 9.38 | 3 | 1.0124 | 0.942188 | 0.8856 | 0.0812 | 0.8747 | 0.364 |
| S4P4M1 | GTP-binding nuclear protein Ran | 54.93 | 1 | 1.01216 | 0.894534 | 1.0488 | 0.5522 | 1.0362 | 0.65 |
| Q2F5Y8 | Macrophage migration inhibitory factor | 26.05 | 3 | 1.01175 | 0.903558 | 1.0689 | 0.0175 | 1.0565 | 0.457 |
| L0N4K9 | Cytochrome P450 | 3.73 | 1 | 1.01134 | 0.926349 | 1.0942 | 0.0277 | 1.082 | 0.393 |
| E9L3M4 | Yellow-f | 9.32 | 2 | 1.01053 | 0.916225 | 1.0577 | 0.4611 | 1.0467 | 0.617 |
| Q2F5P5 | Mo-molybdopterin cofactor sulfurase | 5.65 | 1 | 1.00983 | 0.914439 | 0.9022 | 0.2644 | 0.8935 | 0.267 |
| S4PFG1 | Ras oncogene at 85D | 19.85 | 1 | 1.00893 | 0.957976 | 1.0511 | 0.7127 | 1.0418 | 0.706 |
| A7UFP8 | Elongation factor 1-alpha | 43.52 | 2 | 1.00892 | 0.930358 | 0.9077 | 0.1362 | 0.8996 | 0.236 |
| D2Y4R7 | Coatomer protein complex subunit zeta | 34.97 | 4 | 1.00871 | 0.874863 | 0.9925 | 0.8467 | 0.9839 | 0.723 |
| Q1HQC8 | ARP1 actin-related protein 1-like protein A | 23.67 | 6 | 1.00839 | 0.846712 | 1.0323 | 0.5326 | 1.0237 | 0.597 |
| L7QKE7 | Ala-tRNA synthetase | 13.25 | 2 | 1.00835 | 0.826951 | 1.0119 | 0.8447 | 1.0035 | 0.945 |
| Q3S2I9 | Phosphoglyceromutase | 34.9 | 1 | 1.00828 | 0.875066 | 1.255 | 0.01 | 1.2447 | 0.003 |
| G6CUQ0 | Putative deoxyribonuclease ii | 2.57 | 1 | 1.0077 | 0.929522 | 1.1778 | 0.312 | 1.1688 | 0.248 |
| C0H6P8 | Putative cuticle protein | 28.42 | 5 | 1.00739 | 0.827878 | 1.1086 | 0.0175 | 1.1005 | 0.028 |
| Q2HXS6 | Actin | 58.87 | 1 | 1.00672 | 0.837214 | 1.6273 | 0.0007 | 1.6164 | 2E-05 |
| Q1HPR5 | NADH-ubiquinone reductase | 44.9 | 9 | 1.0059 | 0.709516 | 1.0928 | 0.0117 | 1.0864 | 0.005 |
| I4DPL0 | Ribosome biogenesis protein WDR12 homolog | 4.15 | 1 | 1.00539 | 0.777051 | 0.983 | 0.7095 | 0.9777 | 0.549 |
| Q2F635 | Adenosine kinase | 23.78 | 5 | 1.00524 | 0.851017 | 1.0301 | 0.2722 | 1.0248 | 0.369 |
| Q2F671 | NIPSNAP protein | 16.55 | 2 | 1.00501 | 0.942495 | 1.0693 | 0.1843 | 1.0639 | 0.33 |
| S4PCN9 | Sortilin-related receptor | 0.73 | 1 | 1.00473 | 0.895977 | 0.9892 | 0.8653 | 0.9845 | 0.734 |
| S4PGG9 | Pre-mRNA-splicing factor SLU7 | 1.05 | 1 | 1.00445 | 0.979124 | 0.9542 | 0.455 | 0.9499 | 0.699 |
| I4DR08 | Ras-related protein Rab-14 | 26.54 | 1 | 1.00404 | 0.827084 | 0.9864 | 0.7276 | 0.9824 | 0.565 |
| G3K8S0 | Heat shock protein | 38.96 | 1 | 1.00366 | 0.921603 | 0.9756 | 0.3963 | 0.972 | 0.22 |
| C0H6H7 | Putative cuticle protein | 14.77 | 1 | 1.00267 | 0.983849 | 0.9141 | 0.1508 | 0.9117 | 0.414 |
| E9JEJ4 | Ribosomal protein S6 kinase | 1.99 | 1 | 1.00124 | 0.954937 | 0.9755 | 0.9148 | 0.9743 | 0.883 |
| H9J0E7 | Lysine--tRNA ligase | 9.83 | 5 | 0.99936 | 0.966682 | 0.9192 | 0.0641 | 0.9198 | 0.029 |
| S4P8V7 | 5'-AMP-activated protein kinase catalytic subunit alpha-2 | 8.79 | 1 | 0.99828 | 0.991075 | 1.0504 | 0.6707 | 1.0522 | 0.724 |
| A0T1L9 | Moesin | 40 | 1 | 0.99772 | 0.979088 | 1.3738 | 0.0027 | 1.3769 | 0.006 |
| S4PZQ7 | Constitutive coactivator of PPAR-gamma-like protein 2 | 1.86 | 1 | 0.99735 | 0.987929 | 0.8372 | 0.0784 | 0.8394 | 0.289 |
| G6CXQ4 | eIF2B-alpha protein | 7.24 | 2 | 0.99636 | 0.951372 | 0.9519 | 0.2721 | 0.9554 | 0.341 |
| C7C325 | Elongation factor-1 alpha | 58.37 | 1 | 0.99588 | 0.957655 | 0.7008 | 0.0014 | 0.7037 | 0.007 |
| S4PMT5 | Barrier-to-autointegration factor | 37.78 | 1 | 0.99543 | 0.966131 | 1.0705 | 0.1931 | 1.0754 | 0.419 |
| A5A797 | Acetoacetyl-CoA thiolase | 59.31 | 1 | 0.9949 | 0.949297 | 1.2883 | 0.1034 | 1.2949 | 0.058 |
| E5EVW4 | Aldo-keto reductase | 3.63 | 1 | 0.99401 | 0.964042 | 0.9745 | 0.5309 | 0.9804 | 0.85 |
| I4DRM3 | Suppressor of sable | 4.82 | 1 | 0.99333 | 0.908286 | 1.0506 | 0.5825 | 1.0576 | 0.44 |
| S4P6J5 | Kinesin heavy chain | 10.33 | 1 | 0.99319 | 0.92449 | 0.9569 | 0.5516 | 0.9635 | 0.457 |
| H9JK90 | Ubiquitin carboxyl-terminal hydrolase | 17.96 | 12 | 0.99298 | 0.938652 | 1.1141 | 0.0049 | 1.122 | 0.14 |
| C9DTM4 | CAP isoform A | 3.56 | 2 | 0.99287 | 0.601937 | 0.985 | 0.7685 | 0.992 | 0.837 |
| H9J7H6 | Clustered mitochondria protein homolog | 2.04 | 3 | 0.99262 | 0.69944 | 0.8056 | 0.005 | 0.8116 | 0.001 |
| B4XVB1 | Cytochrome c oxidase subunit | 23.4 | 1 | 0.99168 | 0.89622 | 1.0956 | 0.2291 | 1.1048 | 0.126 |
| S4P5Y2 | Liquid facets | 5.03 | 1 | 0.99155 | 0.918823 | 1.0015 | 0.9186 | 1.01 | 0.875 |
| H9J1X8 | Adenylyl cyclase-associated protein | 41.93 | 10 | 0.99103 | 0.9289 | 1.1235 | 0.0146 | 1.1337 | 0.148 |
| Q1HQB7 | Proteasome subunit alpha type | 35.47 | 6 | 0.99061 | 0.617872 | 1.0798 | 0.0656 | 1.09 | 0.02 |
| I4DMU6 | Eukaryotic translation initiation factor 3 subunit E | 17.98 | 1 | 0.9904 | 0.901657 | 1.0431 | 0.5801 | 1.0532 | 0.388 |
| S4PXV2 | Chromodomain-helicase-DNA-binding protein 1 | 0.67 | 1 | 0.98983 | 0.917016 | 0.9076 | 0.289 | 0.917 | 0.408 |
| Q6L609 | Elongation factor 1 beta | 48.77 | 1 | 0.98924 | 0.778014 | 0.8868 | 0.0433 | 0.8964 | 0.048 |
| G6CU99 | Lon protease homolog | 4.52 | 1 | 0.98833 | 0.666998 | 0.9854 | 0.4389 | 0.997 | 0.886 |
| Q5CCL3 | Peptidyl-prolyl cis-trans isomerase | 65.45 | 8 | 0.98812 | 0.846677 | 1.0042 | 0.9016 | 1.0163 | 0.751 |
| G6CIJ1 | Autophagy related protein Atg12-like protein | 5.74 | 1 | 0.98777 | 0.852612 | 1.0965 | 0.0675 | 1.1101 | 0.102 |
| E3VQ37 | P70S6K | 6.75 | 3 | 0.98649 | 0.740466 | 0.8475 | 0.0199 | 0.8591 | 0.023 |
| Q1HQ81 | Nascent polypeptide associated complex protein alpha subunit | 29.38 | 5 | 0.98563 | 0.45548 | 0.9611 | 0.2273 | 0.9752 | 0.348 |
| S4PCP2 | Protein ROP | 3.27 | 2 | 0.98445 | 0.806228 | 1.1273 | 0.1903 | 1.1451 | 0.052 |
| A5JNM1 | CAMP-dependent protein kinase C1 | 9.92 | 3 | 0.98426 | 0.93161 | 1.1216 | 0.0849 | 1.1395 | 0.367 |
| H8PHZ9 | Elongation factor 1 alpha | 45.15 | 1 | 0.98394 | 0.838825 | 0.9085 | 0.295 | 0.9234 | 0.043 |
| S4PMJ5 | Protein-tyrosine sulfotransferase | 3.4 | 1 | 0.98288 | 0.711966 | 0.909 | 0.0712 | 0.9248 | 0.088 |
| S4P8S3 | Nejire | 1.04 | 1 | 0.98282 | 0.901757 | 0.89 | 0.1392 | 0.9056 | 0.37 |
| H9J9S0 | T-complex protein 1 subunit alpha | 27.21 | 12 | 0.98259 | 0.647326 | 0.8869 | 0.0156 | 0.9026 | 0.024 |
| G6D9Q2 | Shaggy | 13.96 | 5 | 0.98075 | 0.398102 | 1.0723 | 0.1348 | 1.0933 | 0.031 |
| G6DNZ3 | Paternally expressed 3 | 1.81 | 2 | 0.98056 | 0.163482 | 0.929 | 0.0274 | 0.9474 | 0.019 |
| Q5UAS7 | Ribosomal protein L12 | 30.49 | 3 | 0.98042 | 0.750746 | 0.7798 | 0.016 | 0.7954 | 0.018 |
| S4P6I1 | Transportin | 4.41 | 1 | 0.97993 | 0.612115 | 1.0025 | 0.9738 | 1.0231 | 0.695 |
| A0MA79 | TIN-ag-RP | 27.48 | 8 | 0.97969 | 0.843072 | 1.1879 | 0.0113 | 1.2125 | 0.053 |
| Q0ZB80 | EIF2B-beta protein | 7.37 | 1 | 0.97961 | 0.681038 | 0.9479 | 0.4039 | 0.9676 | 0.554 |
| Q0VJ88 | Interstitial collagenase | 7.36 | 1 | 0.97913 | 0.770575 | 1.101 | 0.3856 | 1.1245 | 0.178 |
| Q2F5N1 | Mitochondrial prohibitin complex protein 2 | 58.53 | 3 | 0.97879 | 0.683705 | 0.9881 | 0.5509 | 1.0095 | 0.817 |
| H9JCR8 | Ubiquitin carboxyl-terminal hydrolase | 10.31 | 12 | 0.97853 | 0.840489 | 0.9824 | 0.6036 | 1.004 | 0.963 |
| A0FDQ5 | Dolichyl-diphosphooligosaccharide protein glycotransferase | 34.86 | 4 | 0.97802 | 0.80263 | 0.9098 | 0.0241 | 0.9302 | 0.348 |
| F2WZH2 | Reverse transcriptase | 3.26 | 1 | 0.97736 | 0.799978 | 0.91 | 0.0967 | 0.9311 | 0.384 |
| H9JK49 | Annexin | 35.4 | 13 | 0.97507 | 0.194726 | 1.0053 | 0.7894 | 1.031 | 0.172 |
| Q9BLC5 | Heat shock protein 83 | 62.71 | 27 | 0.97438 | 0.826849 | 0.97 | 0.5516 | 0.9955 | 0.963 |
| S4P2M1 | Vacuole membrane protein 1 | 9.17 | 1 | 0.97397 | 0.526785 | 0.9429 | 0.0942 | 0.9681 | 0.348 |
| H9JU13 | Eukaryotic translation initiation factor 3 subunit A | 17.66 | 16 | 0.97352 | 0.250815 | 0.831 | 0.0112 | 0.8536 | 0.004 |
| B3F882 | Mago nashi | 4.11 | 1 | 0.97346 | 0.82629 | 0.9667 | 0.5588 | 0.9931 | 0.946 |
| Q2F5Q1 | Lysophospholipase | 21.82 | 4 | 0.97329 | 0.865339 | 1.0896 | 0.1711 | 1.1195 | 0.382 |
| G6DL17 | Putative G protein beta subunit-like protein | 3.49 | 1 | 0.97266 | 0.906157 | 0.8713 | 0.0726 | 0.8958 | 0.582 |
| S4P9Q0 | ATP-dependent zinc metalloprotease YME1-like protein | 2.62 | 1 | 0.97189 | 0.521506 | 0.9677 | 0.1204 | 0.9957 | 0.897 |
| D2KMR2 | Putative peptidase | 13.36 | 7 | 0.97183 | 0.100947 | 1.0565 | 0.0622 | 1.0871 | 0.003 |
| D2Y4R4 | Coatomer protein complex subunit beta 2 | 5.45 | 6 | 0.97179 | 0.123095 | 0.9424 | 0.0115 | 0.9697 | 0.066 |
| S4NI84 | Splicing factor 3B subunit 2 | 4.59 | 1 | 0.97156 | 0.696406 | 1.0466 | 0.7844 | 1.0772 | 0.591 |
| Q4ZIR3 | Beta-actin | 47.69 | 1 | 0.97143 | 0.710449 | 1.059 | 0.3515 | 1.0901 | 0.212 |
| Q2F5R2 | G protein pathway suppressor 1 | 1.25 | 1 | 0.97136 | 0.812393 | 0.9581 | 0.5089 | 0.9864 | 0.869 |
| Q2F5L1 | Signal peptidase 18 kDa subunit | 22.47 | 1 | 0.97062 | 0.791156 | 0.9428 | 0.3032 | 0.9713 | 0.752 |
| G9LPV9 | UDP-glycosyltransferase UGT44A1 | 21.14 | 9 | 0.96957 | 0.498935 | 1.0295 | 0.2541 | 1.0618 | 0.16 |
| Q1HQ82 | Methylthioadenosine phosphorylase | 5.02 | 1 | 0.96934 | 0.651452 | 1.064 | 0.3537 | 1.0976 | 0.029 |
| S4PPF6 | U6 snRNA-associated Sm-like protein LSm2 | 48.42 | 3 | 0.96909 | 0.689967 | 1.0652 | 0.0412 | 1.0992 | 0.144 |
| C8C3D2 | Ribosomal protein S5 | 36.31 | 1 | 0.96874 | 0.164877 | 0.8819 | 0.002 | 0.9103 | 0.001 |
| S4PQY1 | Cyclin-G-associated kinase | 12.02 | 2 | 0.96754 | 0.531727 | 0.9772 | 0.5302 | 1.01 | 0.834 |
| S4NJS1 | Dynamin related protein 1 | 6.42 | 1 | 0.96677 | 0.744985 | 0.9637 | 0.5726 | 0.9969 | 0.973 |
| S4PTA5 | UPF0172 protein | 3.95 | 1 | 0.96654 | 0.170552 | 1.0327 | 0.1719 | 1.0684 | 0.024 |
| D4NXP4 | Cell differentiation protein | 2.81 | 1 | 0.96581 | 0.640825 | 0.9342 | 0.5047 | 0.9672 | 0.663 |
| S4PEX5 | ATP citrate lyase | 10.4 | 5 | 0.96571 | 0.822325 | 1.1107 | 0.0572 | 1.1501 | 0.265 |
| D6BNT9 | Glucose-6-phosphate isomerase | 5.04 | 1 | 0.96541 | 0.37963 | 0.9586 | 0.2845 | 0.993 | 0.794 |
| D2Y4R2 | Coatomer protein complex subunit alpha | 18.13 | 17 | 0.96518 | 0.02713 | 0.8917 | 0.0314 | 0.9238 | 0.033 |
| E3UJZ8 | Calmodulin | 51.68 | 9 | 0.96502 | 0.690331 | 1.1709 | 0.0016 | 1.2134 | 0.031 |
| Q1HPL7 | OCIA domain containing protein 2 | 3.74 | 1 | 0.96436 | 0.824367 | 1.1947 | 0.0472 | 1.2388 | 0.095 |
| D2Y4R6 | Coatomer protein complex subunit epsilon | 36.09 | 7 | 0.96337 | 0.759574 | 0.9222 | 0.2267 | 0.9573 | 0.682 |
| H2E289 | Thrombospondin type-1 domain-containing protein 4-like protein | 2.61 | 1 | 0.96329 | 0.806457 | 1.0079 | 0.9104 | 1.0463 | 0.705 |
| S6B5B9 | Short type 6-pyrovoyltetrahydropterin synthase | 14.38 | 2 | 0.96304 | 0.465608 | 0.9154 | 0.1265 | 0.9505 | 0.259 |
| B9U7F2 | Elongation factor 1-alpha | 41.56 | 1 | 0.96262 | 0.775526 | 0.799 | 0.1458 | 0.8301 | 0.085 |
| E5LCP3 | ATP-binding cassette sub-family F member 2 | 3.14 | 1 | 0.96227 | 0.577479 | 0.7013 | 0.0005 | 0.7288 | 0.005 |
| S5TCZ8 | Ribosomal protein S5 | 13.13 | 2 | 0.96184 | 0.567077 | 0.9203 | 0.2354 | 0.9568 | 0.531 |
| H9JM68 | Ribonucleoside-diphosphate reductase | 4.51 | 3 | 0.96095 | 0.800288 | 0.9493 | 0.2421 | 0.9879 | 0.923 |
| D2Y4R5 | Coatomer protein complex subunit delta | 16.37 | 7 | 0.9609 | 0.461548 | 0.9301 | 0.0044 | 0.968 | 0.447 |
| S4NMZ3 | Reticulon-like protein | 9.42 | 2 | 0.96053 | 0.687478 | 1.0868 | 0.0757 | 1.1315 | 0.155 |
| Q402D5 | Juvenile hormone binding protein | 4.08 | 1 | 0.9603 | 0.382863 | 1.2282 | 0.0056 | 1.279 | 3E-04 |
| E3VQ36 | Rock1 | 1.47 | 2 | 0.95986 | 0.67587 | 0.9383 | 0.3382 | 0.9776 | 0.766 |
| E9JEI1 | Survivin-1 | 2.41 | 7 | 0.95947 | 0.432304 | 0.9533 | 0.41 | 0.9936 | 0.91 |
| H9J6N4 | Synaptosomal-associated protein | 3.7 | 1 | 0.95804 | 0.647848 | 1.1022 | 0.5792 | 1.1505 | 0.362 |
| B8Y1A2 | Elongation factor 1-alpha | 54.36 | 1 | 0.95796 | 0.466756 | 0.8808 | 0.0953 | 0.9195 | 0.034 |
| Q1HQ14 | Signal peptidase complex subunit 3 | 11.17 | 1 | 0.95747 | 0.31328 | 1.019 | 0.703 | 1.0643 | 0.236 |
| G6CZV1 | Putative kinesin heavy chain | 1.95 | 1 | 0.95728 | 0.235625 | 0.9937 | 0.9 | 1.0381 | 0.413 |
| E9KFX5 | Arg methyltransferase | 14.75 | 4 | 0.95631 | 0.147066 | 0.9684 | 0.4282 | 1.0126 | 0.719 |
| F5BYH7 | Aldo-keto reductase | 9.46 | 1 | 0.95545 | 0.672595 | 0.995 | 0.8946 | 1.0414 | 0.64 |
| Q1HQ80 | NADH dehydrogenase-ubiquinone Fe-S protein 2 | 32.19 | 7 | 0.95522 | 0.234125 | 0.9842 | 0.4725 | 1.0304 | 0.354 |
| Q2F5L0 | Signal sequence receptor | 7.89 | 2 | 0.95472 | 0.700043 | 0.7754 | 0.0617 | 0.8122 | 0.152 |
| Q2F679 | U6 snRNA-associated Sm-like protein | 21.19 | 1 | 0.95394 | 0.300783 | 0.8475 | 0.0682 | 0.8884 | 0.073 |
| H9IVF7 | Branched-chain-amino-acid aminotransferase | 4.46 | 1 | 0.95277 | 0.828127 | 1.475 | 0.0088 | 1.5482 | 0.026 |
| Q1HPV5 | Muscular protein 20 | 10.99 | 1 | 0.95269 | 0.681843 | 1.0335 | 0.3804 | 1.0848 | 0.365 |
| S4P7P7 | Putative tyrosine-protein phosphatase non-receptor type 9-like protein | 15.38 | 1 | 0.95128 | 0.778214 | 0.8974 | 0.2304 | 0.9433 | 0.674 |
| I4DJP9 | Tyrosyl-tRNA synthetase | 5.87 | 1 | 0.9509 | 0.482075 | 0.927 | 0.4048 | 0.9749 | 0.758 |
| Q1HQ69 | WD repeat domain 61 | 6.99 | 2 | 0.94983 | 0.422172 | 0.9776 | 0.731 | 1.0292 | 0.676 |
| G6DDQ0 | Moesin | 39.04 | 1 | 0.94916 | 0.462749 | 0.9261 | 0.3049 | 0.9757 | 0.734 |
| A3QVV0 | Small nuclear ribonucleoprotein sm d2 | 32.77 | 2 | 0.9485 | 0.142046 | 0.9282 | 0.2794 | 0.9786 | 0.677 |
| G6DK31 | Putative lethal giant larvae protein | 10.38 | 5 | 0.94843 | 0.028214 | 0.9941 | 0.5699 | 1.0482 | 0.009 |
| B9VTS0 | Heat shock protein 20.1 | 53.37 | 7 | 0.9478 | 0.697424 | 0.9106 | 0.0142 | 0.9608 | 0.716 |
| S4PV63 | Fmr1 | 25 | 1 | 0.94764 | 0.042678 | 0.9056 | 0.0292 | 0.9556 | 0.095 |
| Q75VN3 | Translationally-controlled tumor protein homolog | 31.4 | 6 | 0.94755 | 0.330189 | 0.9364 | 0.0629 | 0.9882 | 0.779 |
| S4PEX7 | Etl1-like protein | 16 | 1 | 0.94717 | 0.701292 | 0.7636 | 0.4461 | 0.8062 | 0.469 |
| S4PSC3 | Histidyl-tRNA synthetase | 6.59 | 2 | 0.94611 | 0.307787 | 0.9039 | 0.0987 | 0.9553 | 0.167 |
| Q587N3 | Protein disulfide-isomerase like protein ERp57 | 48.68 | 1 | 0.94587 | 0.128892 | 0.9061 | 7E-05 | 0.9579 | 0.121 |
| S4P8E7 | Amidophosphoribosyltransferase | 5.17 | 1 | 0.94537 | 0.422547 | 0.9269 | 0.3722 | 0.9804 | 0.804 |
| Q2F5M9 | Phosphoribosyl pyrophosphate synthetase | 13.21 | 3 | 0.94511 | 0.171287 | 0.903 | 0.1175 | 0.9555 | 0.259 |
| G6CPY9 | Putative myosin IA | 2.16 | 2 | 0.94356 | 0.472548 | 1.0138 | 0.8495 | 1.0744 | 0.386 |
| G6D5G2 | Putative tyrosyl-tRNA synthetase | 5.72 | 1 | 0.94268 | 0.214009 | 0.7584 | 0.0066 | 0.8045 | 0.006 |
| S4P6T9 | Glycosyl-phosphatidyl-inositol-anchored protein | 1.97 | 1 | 0.94228 | 0.357174 | 0.9678 | 0.4468 | 1.0271 | 0.619 |
| G6CXC9 | Period protein | 1.13 | 1 | 0.94112 | 0.428521 | 0.9434 | 0.3452 | 1.0024 | 0.955 |
| Q75PV8 | Ribonuclease L inhibitor homolog | 10.26 | 1 | 0.94053 | 0.291175 | 0.7595 | 0.0015 | 0.8075 | 0.006 |
| A8VTN6 | Type IV collagen | 5.98 | 2 | 0.9402 | 0.277236 | 0.9702 | 0.3665 | 1.032 | 0.395 |
| Q2F6A0 | Exuperantia | 13.32 | 5 | 0.93965 | 0.558415 | 0.8487 | 0.0841 | 0.9032 | 0.342 |
| Q1HQC1 | Mitochondrial ribosomal protein S5 isoform 2 | 2.03 | 1 | 0.93892 | 0.652669 | 0.7191 | 0.0027 | 0.7659 | 0.079 |
| C1PIJ1 | Putative uncharacterized protein | 13.64 | 2 | 0.93874 | 0.048446 | 0.9576 | 0.3457 | 1.02 | 0.515 |
| Q0G9Y6 | Arp2/3 complex subunit | 25.28 | 2 | 0.93837 | 0.317251 | 1.055 | 0.4792 | 1.1243 | 0.061 |
| G6DEE7 | Putative dipeptidyl peptidase iii | 3.72 | 1 | 0.93808 | 0.683414 | 0.902 | 0.0576 | 0.9615 | 0.757 |
| D4QF47 | Ced-6 protein | 15.79 | 7 | 0.93793 | 0.581749 | 0.9361 | 0.0423 | 0.998 | 0.983 |
| Q2F5S1 | Microtubule-associated protein RP/EB family member 3 | 21.61 | 6 | 0.93785 | 0.647649 | 1.0334 | 0.5668 | 1.1019 | 0.401 |
| A1YM11 | Chaperonin | 19.63 | 6 | 0.93785 | 0.03751 | 0.8829 | 0.0183 | 0.9414 | 0.041 |
| G6DEV9 | WD40 protein | 7.01 | 3 | 0.93747 | 0.23041 | 0.9524 | 0.0289 | 1.016 | 0.678 |
| P29522 | Elongation factor 1-beta | 76.13 | 1 | 0.93739 | 0.660165 | 0.9395 | 0.1617 | 1.0023 | 0.984 |
| Q2F605 | Ecdysteroid-regulated 16 kDa protein | 35.17 | 3 | 0.93731 | 0.235837 | 1.0225 | 0.5366 | 1.0909 | 0.074 |
| H9JBI3 | Integrin beta | 21.59 | 1 | 0.93642 | 0.221306 | 1.0789 | 0.0414 | 1.1522 | 0.01 |
| R4X5G1 | Beta-hexosaminidase | 4.27 | 1 | 0.93601 | 0.74571 | 0.8657 | 0.2426 | 0.9249 | 0.645 |
| G6DJ30 | Putative DEAD box ATP-dependent RNA helicase | 19.32 | 8 | 0.93598 | 0.131931 | 0.7995 | 0.0039 | 0.8542 | 0.01 |
| Q1HPG7 | Vacuolar protein sorting 29 isoform 2 | 27.93 | 3 | 0.93519 | 0.554827 | 1.023 | 0.5492 | 1.0939 | 0.334 |
| A7UFR2 | Elongation factor-1 alpha | 38.82 | 1 | 0.9335 | 0.430245 | 0.9052 | 0.2045 | 0.9697 | 0.605 |
| Q5EPV3 | Elongation factor 1-alpha | 57.51 | 1 | 0.93316 | 0.67765 | 0.7874 | 0.033 | 0.8438 | 0.289 |
| A1Z267 | Acetylcholinesterase | 6.9 | 2 | 0.93252 | 0.40704 | 1.086 | 0.147 | 1.1646 | 0.064 |
| A8D8P8 | PIWI | 11.46 | 8 | 0.93218 | 0.023937 | 0.8688 | 0.0079 | 0.9321 | 0.006 |
| S4PEX8 | Acylamino-acid-releasing enzyme | 10.62 | 1 | 0.93197 | 0.630022 | 0.9459 | 0.5651 | 1.015 | 0.884 |
| K7WS29 | Elongation factor 1 alpha | 63.27 | 1 | 0.93057 | 0.215221 | 1.0907 | 0.39 | 1.1721 | 0.101 |
| B2ZA60 | Chitinase | 2.22 | 1 | 0.93007 | 0.814209 | 0.8002 | 0.0699 | 0.8604 | 0.563 |
| S4P946 | Abhydrolase domain-containing protein 12 | 2.41 | 1 | 0.92994 | 0.568817 | 0.8215 | 0.0814 | 0.8834 | 0.294 |
| S4NWW1 | Synaptobrevin | 23.01 | 2 | 0.92884 | 0.436236 | 0.9167 | 0.2266 | 0.9869 | 0.87 |
| G6CTF0 | Oxysterol-binding protein | 0.59 | 1 | 0.92859 | 0.220699 | 1.0165 | 0.8157 | 1.0947 | 0.213 |
| Q1PCB0 | Pyridoxine 5'-phosphate oxidase | 3.11 | 1 | 0.92843 | 0.496922 | 0.9691 | 0.4431 | 1.0438 | 0.614 |
| G6CSQ5 | Putative CDK5 regulatory subunit associated protein 3 | 2.39 | 1 | 0.92808 | 0.566545 | 1.0036 | 0.951 | 1.0814 | 0.473 |
| G6CRF4 | Ribose-phosphate pyrophosphokinase 1,2 | 7.89 | 2 | 0.92783 | 0.37927 | 0.8868 | 0.1474 | 0.9557 | 0.565 |
| E9JEH4 | Endog | 10.45 | 2 | 0.92747 | 0.01853 | 1.0393 | 0.0272 | 1.1205 | 0.001 |
| S4PWN8 | Kinesin light chain | 6.13 | 2 | 0.92667 | 0.026841 | 0.9066 | 0.0133 | 0.9783 | 0.188 |
| C0H6L0 | Putative cuticle protein | 22.1 | 4 | 0.92608 | 0.101299 | 0.8988 | 0.0225 | 0.9706 | 0.42 |
| Q587N4 | Protein disulfide-isomerase like protein ERp57 | 48.68 | 1 | 0.92499 | 0.264829 | 0.8587 | 0.0847 | 0.9284 | 0.044 |
| D2Y4R3 | Coatomer protein complex subunit beta | 11.87 | 1 | 0.92456 | 0.719206 | 0.9222 | 0.2976 | 0.9974 | 0.988 |
| G6DLA5 | ABC1 family protein | 3.02 | 1 | 0.92443 | 0.675186 | 0.7327 | 0.046 | 0.7926 | 0.159 |
| L0N722 | Cytochrome P450 | 8.72 | 2 | 0.92441 | 0.534035 | 1.0457 | 0.6129 | 1.1312 | 0.285 |
| A7L7U2 | Elongation factor 1 alpha | 61.18 | 4 | 0.92378 | 0.343935 | 0.8702 | 0.044 | 0.942 | 0.398 |
| H9JY38 | Galectin | 10.96 | 2 | 0.92375 | 0.05588 | 1.057 | 0.0577 | 1.1443 | 0.002 |
| G6CPA0 | Katanin p80 WD40 repeat-containing subunit B1 | 2.4 | 2 | 0.92369 | 0.519744 | 0.9851 | 0.8544 | 1.0665 | 0.559 |
| P21894 | Alanine--tRNA ligase | 12.62 | 9 | 0.92351 | 0.224446 | 0.8092 | 0.0018 | 0.8762 | 0.05 |
| I4DPZ1 | Serine protease | 2.28 | 1 | 0.9234 | 0.330683 | 0.9304 | 0.1115 | 1.0075 | 0.894 |
| I4DN12 | Simila to CG6040 | 3.41 | 1 | 0.92113 | 0.202228 | 0.8332 | 0.0682 | 0.9045 | 0.174 |
| S4PWD5 | Dipeptidyl peptidase 9 | 4.2 | 1 | 0.92069 | 0.204931 | 1.0094 | 0.9481 | 1.0964 | 0.428 |
| A4L7H8 | Ribosomal protein S28 | 49.23 | 3 | 0.9206 | 0.0367 | 0.77 | 0.0019 | 0.8364 | 0.003 |
| D2WJ53 | Enolase | 33.6 | 1 | 0.92044 | 0.421799 | 1.0174 | 0.8531 | 1.1053 | 0.223 |
| S4PAG1 | Dolichyl-diphosphooligosaccharide protein glycotransferase | 22.76 | 1 | 0.91983 | 0.097409 | 0.8434 | 0.0113 | 0.917 | 0.048 |
| I1E4Y7 | Eukaryotic translation initiation factor 3 subunit G | 14.96 | 4 | 0.91898 | 0.303703 | 0.7844 | 0.0012 | 0.8535 | 0.062 |
| E9JEI6 | Parp | 8.39 | 6 | 0.91892 | 0.534528 | 1.04 | 0.5264 | 1.1318 | 0.29 |
| H9J2M2 | Transcription elongation factor SPT5 | 3.23 | 2 | 0.91862 | 0.167187 | 0.8502 | 0.0062 | 0.9255 | 0.137 |
| S4PW98 | Smad nuclear interacting protein 1 | 3.7 | 1 | 0.91769 | 0.286771 | 0.7586 | 0.0576 | 0.8266 | 0.019 |
| Q9NKX3 | Coatomer subunit gamma | 9.06 | 8 | 0.91768 | 0.024568 | 0.8881 | 0.0088 | 0.9677 | 0.042 |
| Q1HPY4 | Sec13-like protein | 6.1 | 2 | 0.91704 | 0.120002 | 0.962 | 0.3925 | 1.049 | 0.035 |
| M4PZX1 | Actin 1 | 53.72 | 1 | 0.91698 | 0.213376 | 0.9602 | 0.1424 | 1.0471 | 0.369 |
| L7QP70 | Carbamoylphosphate synthetase/aspartate transcarbamylase/dihydroorotase | 1.65 | 1 | 0.9169 | 0.056882 | 0.9674 | 0.7556 | 1.0551 | 0.532 |
| D3X5F2 | Elongation factor 1-alpha | 37.14 | 1 | 0.91591 | 0.230839 | 0.8469 | 0.0582 | 0.9247 | 0.215 |
| I4DQA5 | Charged multivesicular body protein | 8.37 | 2 | 0.91515 | 0.508311 | 1.0443 | 0.2539 | 1.1411 | 0.229 |
| C3W4P4 | Microtubule-associated protein | 32.92 | 1 | 0.91514 | 0.278277 | 0.953 | 0.2795 | 1.0414 | 0.475 |
| G6DGW7 | Proliferating cell nuclear antigen | 5.77 | 1 | 0.91511 | 0.464612 | 0.8605 | 0.0049 | 0.9403 | 0.528 |
| C0H6M9 | Putative cuticle protein | 40.44 | 6 | 0.91468 | 0.265853 | 0.9401 | 0.0253 | 1.0278 | 0.627 |
| I3VR83 | Aminopeptidase N-12 | 7.59 | 5 | 0.91413 | 0.043419 | 0.8601 | 0.0004 | 0.9409 | 0.059 |
| C0H6L4 | Putative cuticle protein | 65.38 | 4 | 0.91376 | 0.014064 | 0.9995 | 0.9782 | 1.0938 | 0.003 |
| Q1HPQ5 | Serine proteinase-like protein | 22.56 | 7 | 0.91349 | 0.232932 | 0.8988 | 0.0367 | 0.9839 | 0.782 |
| I4DNU9 | Malic enzyme | 3.41 | 1 | 0.91264 | 0.758416 | 0.7439 | 0.0456 | 0.8151 | 0.461 |
| Q09GR7 | Larval cuticle protein 1 | 54.39 | 3 | 0.91252 | 0.169731 | 0.9424 | 0.0094 | 1.0328 | 0.47 |
| P80034 | Antichymotrypsin-2 | 69.6 | 1 | 0.91041 | 0.297485 | 1.0126 | 0.662 | 1.1123 | 0.144 |
| Q2I355 | Prophenoloxidase | 4.19 | 1 | 0.91001 | 0.350119 | 1.0104 | 0.9102 | 1.1103 | 0.02 |
| S4NZG0 | Peptidyl-prolyl cis-trans isomerase | 3.92 | 1 | 0.90955 | 0.445464 | 0.8522 | 0.0016 | 0.937 | 0.514 |
| Q1HPW2 | Eukaryotic translation initiation factor 4A | 41.43 | 15 | 0.90942 | 0.312941 | 0.8612 | 0.003 | 0.947 | 0.46 |
| S4NQH4 | Membrane-associated protein | 24.05 | 2 | 0.90906 | 0.2397 | 0.9319 | 0.2193 | 1.0251 | 0.707 |
| Q8MY95 | Elongation factor 1-alpha | 68.24 | 1 | 0.90687 | 0.080557 | 0.9149 | 0.2341 | 1.0088 | 0.841 |
| Q0N2S2 | Small GTP-binding protein Rab10 | 28.57 | 4 | 0.90647 | 0.015665 | 0.9291 | 0.2 | 1.025 | 0.555 |
| Q2F654 | Sr protein | 12.99 | 2 | 0.90615 | 0.033738 | 0.8669 | 0.0004 | 0.9566 | 0.103 |
| G6CVC0 | Myosin light polypeptide 9 isoform B | 39.08 | 1 | 0.90479 | 0.399969 | 0.8321 | 0.0208 | 0.9196 | 0.418 |
| G6D4E3 | Eukaryotic translation termination factor 1 | 9.88 | 2 | 0.90387 | 0.008088 | 0.8056 | 0.0063 | 0.8913 | 0.007 |
| D8UYI6 | Elongation factor 1-alpha | 55.64 | 1 | 0.90318 | 0.104119 | 0.7635 | 0.004 | 0.8453 | 0.024 |
| Q4F863 | Eukaryotic initiation factor 5A | 43.75 | 7 | 0.90275 | 0.260659 | 0.9188 | 0.0246 | 1.0178 | 0.79 |
| I4DPW9 | Mitochondrial ribosomal protein L45 | 6.37 | 1 | 0.90248 | 0.050284 | 0.884 | 0.0414 | 0.9795 | 0.592 |
| S4PXN6 | Exocyst complex component 5 | 2.04 | 1 | 0.90225 | 0.065793 | 0.8557 | 0.0812 | 0.9484 | 0.35 |
| Q2F6C2 | Chaperonin containing t-complex polypeptide 1 beta subunit | 37.31 | 17 | 0.89957 | 0.004599 | 0.838 | 0.0096 | 0.9315 | 0.032 |
| L8B6C5 | Glucosidase II beta-subunit | 7.07 | 4 | 0.89955 | 0.435283 | 0.893 | 0.0636 | 0.9927 | 0.943 |
| Q1HE02 | ALY | 29.13 | 5 | 0.89939 | 0.014507 | 0.8726 | 0.0001 | 0.9702 | 0.145 |
| O44300 | Elongation factor 1-alpha | 50.85 | 2 | 0.8982 | 0.553019 | 0.6828 | 0.0086 | 0.7602 | 0.151 |
| L0GGU3 | FK506-binding protein | 28.37 | 1 | 0.89624 | 0.297405 | 0.8994 | 0.2388 | 1.0036 | 0.953 |
| Q2F5V5 | Larval cuticle protein | 32.9 | 4 | 0.89594 | 0.136155 | 0.8846 | 0.0054 | 0.9874 | 0.782 |
| H9JAM9 | Heat shock protein 70-14 | 2.11 | 1 | 0.89558 | 0.372383 | 0.8125 | 0.0128 | 0.9072 | 0.355 |
| G1CJY2 | Elongation factor-1alpha | 42.46 | 1 | 0.89462 | 0.620128 | 0.5979 | 0.0023 | 0.6684 | 0.122 |
| S4NV08 | Putative phospholipid-transporting ATPase IA | 13.58 | 1 | 0.89416 | 0.103562 | 0.766 | 4E-05 | 0.8567 | 0.023 |
| E3UKL7 | Myosin heavy chain | 23.81 | 2 | 0.893 | 0.517899 | 0.9964 | 0.9592 | 1.1157 | 0.452 |
| Q2F6C4 | T-complex protein 1 subunit delta | 38.73 | 14 | 0.89283 | 0.007121 | 0.8097 | 9E-05 | 0.9069 | 0.003 |
| Q68HB4 | Profilin | 80.95 | 7 | 0.89276 | 0.252338 | 0.8321 | 2E-05 | 0.9321 | 0.361 |
| D1MBK3 | Integrin beta | 21.85 | 1 | 0.89234 | 0.327578 | 0.919 | 0.4105 | 1.0299 | 0.797 |
| G6DR63 | Held out wings | 4.68 | 1 | 0.89215 | 0.031624 | 0.8981 | 0.0117 | 1.0067 | 0.774 |
| Q9GN07 | Heat shock protein 20.8 | 51.08 | 3 | 0.89119 | 0.273349 | 1.029 | 0.4911 | 1.1546 | 0.117 |
| H9JJY4 | Structural maintenance of chromosomes protein | 3.16 | 3 | 0.8907 | 0.030845 | 0.79 | 0.0003 | 0.8869 | 0.01 |
| H9J0I1 | DNA-directed RNA polymerase | 3.73 | 1 | 0.88976 | 0.056853 | 0.7462 | 0.0106 | 0.8387 | 0.003 |
| G6DS27 | Putative wd-repeat protein | 1.46 | 1 | 0.88954 | 0.113435 | 0.8956 | 0.1022 | 1.0068 | 0.893 |
| Q0KKW8 | Multi-binding protein | 11.32 | 3 | 0.8895 | 0.019702 | 1.1155 | 0.3561 | 1.254 | 0.05 |
| G9JS66 | Kinesin | 5.56 | 1 | 0.88944 | 0.215118 | 0.935 | 0.3345 | 1.0512 | 0.515 |
| H9IVA5 | N-acetylglucosamine-6-phosphate deacetylase | 8.29 | 3 | 0.88915 | 0.037596 | 1.0452 | 0.5428 | 1.1755 | 0.049 |
| Q0N2R9 | Signal recognition particle 14 kDa protein-like protein | 13.08 | 1 | 0.88903 | 0.104958 | 0.9623 | 0.3504 | 1.0824 | 0.184 |
| H9JA74 | Malic enzyme | 16.36 | 7 | 0.88894 | 0.005691 | 0.8572 | 0.0008 | 0.9643 | 0.082 |
| G6DLX5 | Autophagy-related protein 3 | 4.67 | 1 | 0.88851 | 0.061286 | 0.8458 | 0.0579 | 0.9519 | 0.272 |
| Q1HQA9 | Transmembrane trafficking protein | 20.98 | 5 | 0.88818 | 0.421204 | 0.8552 | 0.055 | 0.9628 | 0.756 |
| C0H6P7 | Putative cuticle protein | 3.48 | 1 | 0.88731 | 0.602171 | 0.7208 | 0.0009 | 0.8124 | 0.331 |
| O44309 | Elongation factor 1-alpha | 47.94 | 2 | 0.88674 | 0.361229 | 0.7943 | 0.0926 | 0.8958 | 0.284 |
| Q2F656 | Ribosomal protein L20 | 5.03 | 1 | 0.88615 | 0.093236 | 0.8275 | 0.032 | 0.9338 | 0.268 |
| S4PBR3 | Minichromosome maintenance 2 | 3.75 | 2 | 0.88472 | 0.124427 | 1.1393 | 0.3688 | 1.2877 | 0.069 |
| Q5UAN8 | 40S ribosomal protein S6 | 41.5 | 1 | 0.88443 | 0.014929 | 0.8522 | 0.0076 | 0.9635 | 0.006 |
| D1LYR7 | Ribosomal protein P1 | 47.75 | 1 | 0.88381 | 0.145362 | 0.6483 | 0.0101 | 0.7335 | 0.003 |
| H9JW04 | Coatomer subunit beta | 13.03 | 5 | 0.88371 | 0.03306 | 0.9002 | 0.027 | 1.0186 | 0.62 |
| I4DNU3 | Ptx1 protein | 8.07 | 2 | 0.88346 | 0.477896 | 0.8802 | 0.0716 | 0.9963 | 0.979 |
| A7BEX9 | Imaginal disk growth factor | 45.39 | 19 | 0.88212 | 0.009122 | 0.9419 | 0.0059 | 1.0678 | 0.02 |
| B1P4Q6 | Protein Wnt | 5.26 | 1 | 0.88207 | 0.017881 | 0.8102 | 0.0135 | 0.9185 | 0.029 |
| Q25C94 | Sex-lethal | 4.48 | 1 | 0.88079 | 0.006908 | 0.9029 | 0.0011 | 1.0251 | 0.184 |
| B3TH82 | Ribosomal protein S2 | 65.44 | 1 | 0.88061 | 0.558896 | 0.8822 | 0.2496 | 1.0018 | 0.992 |
| B0LL83 | Pyruvate dehydrogenase kinase | 7.91 | 2 | 0.88008 | 0.012509 | 0.962 | 0.4298 | 1.093 | 0.084 |
| Q3LBA3 | Chemosensory protein 5 | 13.6 | 2 | 0.87882 | 0.185371 | 0.783 | 0.0018 | 0.8909 | 0.143 |
| C8C3H2 | Ribosomal protein S5 | 36.1 | 1 | 0.87755 | 0.101218 | 0.777 | 0.002 | 0.8855 | 0.083 |
| V9XWE8 | DNA-directed RNA polymerase | 2.72 | 1 | 0.87598 | 0.029809 | 0.6308 | 0.0105 | 0.7201 | 0.005 |
| Q2F6C3 | Chaperonin subunit 6a zeta | 36.91 | 14 | 0.8759 | 0.023985 | 0.8388 | 0.0007 | 0.9576 | 0.195 |
| E9JEG7 | App | 3.49 | 2 | 0.87579 | 0.181405 | 0.8369 | 0.0149 | 0.9556 | 0.547 |
| S4P3C9 | Transcription initiation factor IIB | 4.4 | 1 | 0.87569 | 0.396392 | 0.7417 | 0.0092 | 0.847 | 0.219 |
| B9VJ80 | Ubiquitin-like modifier-activating enzyme 5 | 13.74 | 4 | 0.87566 | 0.004887 | 0.842 | 0.0108 | 0.9616 | 0.169 |
| S4P8I0 | Putative H/ACA ribonucleoprotein complex subunit 1-like protein | 12.59 | 1 | 0.87452 | 0.125705 | 0.7843 | 0.0022 | 0.8969 | 0.135 |
| H9JTI9 | Eukaryotic translation initiation factor 3 subunit C | 11.3 | 9 | 0.87425 | 0.004517 | 0.798 | 0.0176 | 0.9128 | 0.089 |
| G6DGM9 | Endothelial-monocyte activating polypeptide II | 9.86 | 2 | 0.8742 | 0.315663 | 0.7357 | 0.0027 | 0.8416 | 0.173 |
| H9IWV6 | Signal transducer and activator of transcription | 5.79 | 3 | 0.87275 | 0.138325 | 0.9378 | 0.0055 | 1.0746 | 0.252 |
| S4NP45 | Autophagy-specific protein | 4.21 | 2 | 0.87155 | 0.373279 | 0.8463 | 0.1348 | 0.9711 | 0.828 |
| C0H6N4 | Putative cuticle protein | 31.45 | 3 | 0.87073 | 0.069141 | 0.8621 | 0.0472 | 0.99 | 0.866 |
| C5IG30 | ACHI protein | 12.9 | 1 | 0.87069 | 0.349725 | 0.9211 | 0.2375 | 1.0579 | 0.632 |
| S4PNJ2 | Vacuolar protein sorting 35 | 3.7 | 1 | 0.86988 | 0.217385 | 0.7978 | 0.0044 | 0.9171 | 0.347 |
| Q5UAU1 | Ribosomal protein P0 | 70.57 | 7 | 0.86931 | 0.004309 | 0.7239 | 1E-04 | 0.8327 | 5E-04 |
| Q8T109 | FK506-binding protein | 64.47 | 4 | 0.86923 | 0.007264 | 0.9005 | 0.0177 | 1.036 | 0.186 |
| G9LPV7 | UDP-glycosyltransferase UGT42B1 | 3.35 | 1 | 0.86862 | 0.018597 | 0.8005 | 0.0051 | 0.9216 | 0.022 |
| H9IVT2 | 40S ribosomal protein S21 | 41.98 | 3 | 0.86843 | 0.067832 | 0.7982 | 0.0037 | 0.9191 | 0.161 |
| Q1HPJ4 | Mitochondrial ribosomal protein S12 | 12.61 | 2 | 0.86822 | 0.013194 | 0.861 | 0.0009 | 0.9917 | 0.733 |
| Q9GV27 | Eukaryotic translation initiation factor 3 subunit H | 17.51 | 4 | 0.86769 | 0.00868 | 0.7689 | 0.0006 | 0.8862 | 0.003 |
| Q4U1M7 | Elongation factor 1-alpha | 46.65 | 1 | 0.86761 | 0.399444 | 0.8846 | 0.0459 | 1.0195 | 0.88 |
| C0H6M5 | Putative cuticle protein | 47.13 | 5 | 0.8652 | 0.001305 | 0.9001 | 0.0044 | 1.0403 | 0.039 |
| D3GGI8 | Beta-hexosaminidase | 5.86 | 2 | 0.86483 | 0.217451 | 0.9125 | 0.4665 | 1.0551 | 0.611 |
| D1LYK2 | Ribosomal protein L11 | 26.29 | 6 | 0.86471 | 0.009296 | 0.7609 | 0.0006 | 0.8799 | 0.008 |
| S4PN47 | Catenin alpha | 4.92 | 1 | 0.86454 | 0.353365 | 0.7776 | 0.0261 | 0.8994 | 0.451 |
| S4P606 | Cheerio | 3.66 | 1 | 0.8645 | 0.025236 | 0.8926 | 0.0091 | 1.0325 | 0.363 |
| H9JDB0 | Ubiquitin carboxyl-terminal hydrolase | 1.12 | 2 | 0.86447 | 0.122811 | 0.8296 | 0.068 | 0.9597 | 0.635 |
| Q1HQ32 | Carboxypeptidase inhibitor | 14.16 | 1 | 0.86254 | 0.558409 | 0.7513 | 0.0342 | 0.871 | 0.51 |
| B7SES9 | Elongation factor 1-alpha | 48.43 | 2 | 0.86248 | 0.071236 | 0.792 | 0.0008 | 0.9183 | 0.137 |
| Q1HQ86 | Mobility group protein 1B | 33.61 | 4 | 0.86137 | 0.286327 | 0.9215 | 0.1953 | 1.0698 | 0.542 |
| Q5UAT8 | Ribosomal protein L3 | 31.23 | 1 | 0.8608 | 0.532724 | 0.7433 | 0.0157 | 0.8635 | 0.5 |
| S4PGH1 | Chaperonin | 16.18 | 1 | 0.86056 | 0.176578 | 0.737 | 0.0208 | 0.8565 | 0.151 |
| G6DLF9 | Venom serine carboxypeptidase | 4.62 | 1 | 0.86018 | 0.305493 | 0.8919 | 0.15 | 1.0369 | 0.752 |
| S4P5N8 | DNA-directed RNA polymerase | 5.23 | 1 | 0.86012 | 0.063281 | 0.7997 | 0.0401 | 0.9297 | 0.352 |
| Q2F6A6 | Elongation factor Tu | 41.08 | 10 | 0.8595 | 0.202146 | 0.8552 | 0.0089 | 0.995 | 0.953 |
| Q1HPK6 | Translation elongation factor 2 | 49.53 | 9 | 0.85945 | 0.121417 | 0.7037 | 0.0002 | 0.8188 | 0.04 |
| F1C930 | Small heat shock protein 22.2 | 17.19 | 1 | 0.85898 | 0.031247 | 0.988 | 0.8194 | 1.1502 | 0.01 |
| Q86G87 | Integrin alpha 2 | 1.02 | 1 | 0.85831 | 0.487391 | 0.8767 | 0.5632 | 1.0215 | 0.801 |
| F8UZA8 | HMGB protein | 7.69 | 1 | 0.85717 | 0.33152 | 0.7485 | 0.0897 | 0.8733 | 0.239 |
| H9JQT5 | Adenylyltransferase and sulfurtransferase MOCS3 | 1.98 | 1 | 0.85711 | 0.049699 | 0.8589 | 0.1167 | 1.0021 | 0.963 |
| Q19N36 | Small GTP binding protein RAB5 | 21.6 | 4 | 0.85672 | 0.087239 | 1.0345 | 0.096 | 1.2076 | 0.016 |
| Q1HQ85 | Mitochondrial ribosomal protein S10 | 3.49 | 1 | 0.85596 | 0.189162 | 0.7647 | 0.0105 | 0.8934 | 0.28 |
| C6K2H4 | Prophenoloxidase subunit 2 | 10.49 | 1 | 0.85558 | 0.177971 | 1.0301 | 0.7651 | 1.204 | 0.069 |
| C0H6N6 | Putative cuticle protein | 11.45 | 2 | 0.85539 | 0.164328 | 0.7818 | 0.0032 | 0.914 | 0.313 |
| S4NSZ5 | Putative U5 small nuclear ribonucleoprotein 200 kDa helicase | 1.67 | 1 | 0.85493 | 0.285398 | 0.9152 | 0.254 | 1.0705 | 0.529 |
| Q1HQ57 | Fructose-1,6-bisphosphatase | 9.23 | 2 | 0.85271 | 0.111174 | 0.8009 | 0.0123 | 0.9392 | 0.366 |
| A9XZ75 | Putative histone deacetylase 1 | 19.79 | 4 | 0.85271 | 0.064322 | 0.9702 | 0.2335 | 1.1378 | 0.036 |
| Q2F674 | Transcription factor A | 6.02 | 1 | 0.85265 | 0.271093 | 0.875 | 0.0798 | 1.0263 | 0.821 |
| S4P9H2 | Protein phosphatase 1 regulatory subunit 11 | 7.04 | 1 | 0.85255 | 0.038992 | 0.9804 | 0.7771 | 1.15 | 0.063 |
| Q2F5P2 | Myosin light polypeptide 9 isoform 2 | 33.7 | 1 | 0.85222 | 0.256965 | 0.8815 | 0.2058 | 1.0344 | 0.709 |
| S4P9R1 | UPF0389 protein | 8.59 | 1 | 0.85159 | 0.083451 | 0.8376 | 0.0219 | 0.9836 | 0.805 |
| S4PD26 | CWF19L1 protein | 5.66 | 1 | 0.85089 | 0.447345 | 1.0806 | 0.6489 | 1.27 | 0.27 |
| S4NWZ4 | Cut up | 58.43 | 5 | 0.85086 | 0.227364 | 0.8888 | 0.1123 | 1.0446 | 0.679 |
| H9JX68 | DNA-directed RNA polymerase | 1.54 | 2 | 0.85037 | 0.023261 | 0.8885 | 0.1995 | 1.0449 | 0.545 |
| Q5UAS9 | Ribosomal protein L10A | 46.54 | 9 | 0.85005 | 0.002604 | 0.7378 | 0.0002 | 0.8679 | 0.001 |
| Q5UC14 | Ribosomal protein L21 | 22.01 | 1 | 0.84988 | 0.11772 | 0.6278 | 0.0127 | 0.7386 | 0.003 |
| Q5MGJ4 | 40S ribosomal protein S29 | 50 | 4 | 0.84944 | 0.282886 | 0.6872 | 0.0011 | 0.809 | 0.15 |
| C0H6G0 | Putative cuticle protein | 14.1 | 2 | 0.84885 | 0.078467 | 1.0727 | 0.2748 | 1.2638 | 0.021 |
| Q9BLG6 | Prophenoloxidase-2s | 41.13 | 1 | 0.84881 | 0.015798 | 1.1458 | 0.0246 | 1.3499 | 3E-04 |
| I4DNE9 | Obstructor-E | 2.35 | 1 | 0.84875 | 0.059164 | 0.8898 | 0.0086 | 1.0484 | 0.379 |
| Q5UAQ8 | Ribosomal protein L30 | 53.1 | 5 | 0.8487 | 0.155228 | 0.7362 | 0.016 | 0.8675 | 0.2 |
| O44303 | Elongation factor 1-alpha | 53.75 | 1 | 0.84856 | 0.458544 | 0.6484 | 0.0478 | 0.7641 | 0.282 |
| I4DM36 | GST-containing FLYWCH zinc-finger protein | 19.31 | 1 | 0.84747 | 0.079833 | 0.6124 | 0.0058 | 0.7226 | 0.014 |
| Q64FN2 | 40S ribosomal protein S3a | 57.03 | 16 | 0.84695 | 0.02663 | 0.7145 | 0.0001 | 0.8436 | 0.011 |
| E7DZ15 | Ribosomal protein S20 | 28.46 | 3 | 0.84626 | 0.261871 | 0.6796 | 1E-04 | 0.8031 | 0.127 |
| H9JNJ2 | Guanine nucleotide-binding protein subunit gamma | 18.57 | 2 | 0.84432 | 0.124955 | 0.8002 | 0.0039 | 0.9477 | 0.482 |
| H9JEK0 | Sulfiredoxin | 8.18 | 1 | 0.84376 | 0.0161 | 0.8611 | 0.0664 | 1.0205 | 0.622 |
| G3K8N5 | Hairy cell leukemia | 7.34 | 1 | 0.8428 | 0.181872 | 0.9444 | 0.3165 | 1.1205 | 0.268 |
| Q1HPV7 | Histone H2A | 31.01 | 2 | 0.84166 | 0.390678 | 0.9029 | 0.2213 | 1.0728 | 0.666 |
| I4DKM8 | Cuticular protein PxutCPR33 | 9.35 | 1 | 0.84152 | 0.095744 | 0.9242 | 0.2328 | 1.0983 | 0.256 |
| Q5UAN6 | 40S ribosomal protein S8 | 52.88 | 10 | 0.8414 | 0.010821 | 0.7659 | 0.0028 | 0.9102 | 0.061 |
| C0H6G1 | Putative cuticle protein | 43.93 | 4 | 0.84109 | 0.005707 | 0.7271 | 0.0042 | 0.8644 | 0.01 |
| G6CPF5 | MAP kinse-ERK kinase | 2.75 | 1 | 0.84084 | 0.23353 | 0.7829 | 0.0585 | 0.931 | 0.484 |
| H9CVW0 | Ribosomal protein S5 | 41.11 | 1 | 0.84065 | 0.205448 | 0.8054 | 0.0148 | 0.9581 | 0.687 |
| Q8T634 | Pupal cuticle protein 36a | 9.64 | 1 | 0.83982 | 0.002658 | 0.7296 | 0.0003 | 0.8688 | 0.002 |
| B8Y182 | Elongation factor 1-alpha | 55.75 | 1 | 0.83978 | 0.066184 | 0.7516 | 0.0007 | 0.895 | 0.122 |
| Q1HPZ6 | Leukotriene A4 hydrolase | 11.22 | 4 | 0.83964 | 0.047213 | 0.8726 | 0.072 | 1.0393 | 0.524 |
| I4DP87 | Similar to CG9154 | 7.01 | 1 | 0.83823 | 0.133347 | 0.7999 | 0.0824 | 0.9542 | 0.296 |
| Q2F651 | Short-chain dehydrogenease/reductase 2 | 29.73 | 1 | 0.83821 | 0.001393 | 0.8889 | 0.014 | 1.0605 | 0.038 |
| F8T9P8 | Isocitrate dehydrogenase | 35.59 | 1 | 0.83788 | 0.335491 | 0.9048 | 0.4628 | 1.0798 | 0.659 |
| Q9BPP5 | Vitellogenin | 38.09 | 1 | 0.83765 | 0.109404 | 0.8973 | 0.5874 | 1.0711 | 0.678 |
| Q6R558 | Trypsin-like proteinase T2b | 2.03 | 1 | 0.83489 | 0.322044 | 0.7386 | 0.0083 | 0.8847 | 0.407 |
| A0FDQ2 | Eukaryotic initiation factor 5C | 20.53 | 9 | 0.83397 | 0.00992 | 0.8252 | 0.0003 | 0.9894 | 0.72 |
| H9U396 | Heat shock protein 90 beta | 47.04 | 31 | 0.83319 | 0.000265 | 0.7036 | 3E-05 | 0.8445 | 6E-05 |
| Q1HQ08 | Eukaryotic translation initiation factor 4E-binding protein 2 | 14.78 | 1 | 0.83055 | 0.153692 | 0.9206 | 0.1096 | 1.1084 | 0.291 |
| Q2F5L8 | Ribosomal protein L7Ae | 15.38 | 2 | 0.83001 | 0.157306 | 0.7345 | 0.0068 | 0.8849 | 0.28 |
| G6DAS7 | Elongation of very long chain fatty acids protein | 1.78 | 1 | 0.82964 | 0.219994 | 1.0563 | 0.5577 | 1.2732 | 0.061 |
| Q1HDZ3 | Rsf1 | 18.18 | 2 | 0.82933 | 0.052383 | 0.806 | 0.0029 | 0.9719 | 0.634 |
| A0FDQ3 | Bax inhibitor-1-like protein | 3.85 | 1 | 0.82899 | 0.050901 | 0.8885 | 0.1014 | 1.0718 | 0.245 |
| S4P6J9 | Vigilin | 8.55 | 2 | 0.8289 | 0.009341 | 0.6758 | 0.0003 | 0.8153 | 0.004 |
| S4PVV8 | Chromodomain Y-like protein | 4.67 | 1 | 0.82881 | 0.027016 | 0.7976 | 0.0158 | 0.9623 | 0.524 |
| Q8WPG8 | Calreticulin | 19.65 | 1 | 0.82859 | 0.074726 | 0.6102 | 0.0149 | 0.7364 | 0.01 |
| Q2F6B9 | Ubiquinone biosynthesis protein COQ7-like protein | 5.1 | 1 | 0.82818 | 0.001227 | 0.9306 | 0.0393 | 1.1236 | 0.005 |
| Q9BPS3 | Elongation factor 1 gamma | 46.34 | 22 | 0.82798 | 0.044586 | 0.7445 | 1E-04 | 0.8992 | 0.108 |
| G6CWF5 | ATP-binding cassette sub-family C member 4 | 2.36 | 1 | 0.82714 | 0.020176 | 1.1441 | 0.197 | 1.3832 | 0.012 |
| S4P1X1 | C-terminal Binding Protein | 9.21 | 3 | 0.82643 | 0.003963 | 0.8759 | 0.0251 | 1.0599 | 0.064 |
| H9IUH3 | Eukaryotic translation initiation factor 3 subunit M | 26.17 | 9 | 0.82562 | 0.046495 | 0.7533 | 0.0017 | 0.9124 | 0.163 |
| Q4AED5 | Atypical protein kinase C | 1.54 | 1 | 0.82493 | 0.203486 | 0.9209 | 0.1404 | 1.1163 | 0.339 |
| I4DQ01 | Clathrin coat assembly protein orange | 6.28 | 1 | 0.8249 | 0.117824 | 0.7863 | 0.0179 | 0.9532 | 0.607 |
| G6DHP0 | Putative tartan | 1.27 | 1 | 0.82458 | 0.080818 | 0.8657 | 0.112 | 1.0499 | 0.365 |
| Q1HPQ7 | Small nuclear ribonucleoprotein protein F | 21.59 | 2 | 0.82389 | 0.01441 | 0.7246 | 0.0069 | 0.8795 | 0.061 |
| F1C4B9 | Stromal cell derived factor 2-like protein 1 | 6.33 | 2 | 0.82287 | 0.142969 | 0.7852 | 0.0116 | 0.9543 | 0.645 |
| G6DHG8 | Dynein heavy chain | 3.54 | 2 | 0.82239 | 0.02684 | 0.8366 | 0.0107 | 1.0173 | 0.681 |
| S4PWE2 | Protein FAM50-like protein | 7.64 | 1 | 0.82012 | 0.002216 | 0.7952 | 0.009 | 0.9696 | 0.415 |
| Q5UAP0 | 40S ribosomal protein S4 | 58.94 | 18 | 0.81994 | 0.046388 | 0.7047 | 0.0004 | 0.8594 | 0.058 |
| G6DF38 | 26S proteasome non-ATPase regulatory subunit 1 | 9.7 | 3 | 0.81989 | 0.086423 | 0.8663 | 0.0409 | 1.0567 | 0.409 |
| I4DNB4 | 40S ribosomal protein SA | 26.45 | 4 | 0.81935 | 0.00591 | 0.7705 | 0.0001 | 0.9404 | 0.063 |
| A9XXI2 | Putative 26S proteasome non-ATPase regulatory subunit 1 | 12.87 | 1 | 0.8188 | 0.073866 | 0.8878 | 0.1229 | 1.0842 | 0.214 |
| G0ZEG4 | Ribosomal protein L8 | 68.09 | 1 | 0.81828 | 0.340575 | 0.7207 | 0.0133 | 0.8808 | 0.465 |
| Q5EPU0 | Elongation factor 1-alpha | 50.28 | 1 | 0.81819 | 0.052632 | 0.7849 | 0.0427 | 0.9594 | 0.335 |
| S4PKK4 | Serine/threonine-protein kinase minibrain | 18.57 | 1 | 0.81755 | 0.02568 | 0.7891 | 0.0277 | 0.9652 | 0.6 |
| Q0ZAL8 | Splicing factor arginine/serine-rich 6 | 11.44 | 3 | 0.81725 | 0.000241 | 0.789 | 0.0001 | 0.9654 | 0.031 |
| Q1HPN0 | Insulin-related peptide binding protein | 2.75 | 1 | 0.81709 | 0.046817 | 0.6991 | 0.0018 | 0.8556 | 0.071 |
| C3PPG5 | DNA sequence from clone AEHM-21P16 | 3.76 | 1 | 0.81672 | 0.001038 | 0.7039 | 9E-05 | 0.8619 | 3E-04 |
| C0H6Z2 | Putative cuticle protein | 7.44 | 1 | 0.8162 | 0.128303 | 0.8923 | 0.2317 | 1.0932 | 0.426 |
| Q2F607 | Erythrocyte carbonic anhydrase | 4.15 | 1 | 0.81572 | 0.075454 | 1.0547 | 0.6315 | 1.293 | 0.055 |
| H9JJE6 | Histone H4 | 52.43 | 7 | 0.81484 | 0.002335 | 0.8345 | 0.0004 | 1.0242 | 0.267 |
| I4DNK1 | NADH-ubiquinone oxidoreductase fe-s protein 2 | 20.42 | 2 | 0.8144 | 0.051825 | 0.851 | 0.0299 | 1.045 | 0.536 |
| H2EVJ9 | Elongation factor 1 alpha | 24.67 | 1 | 0.81399 | 0.01162 | 0.7661 | 0.0037 | 0.9411 | 0.23 |
| G6D8P0 | Peptidyl-prolyl cis-trans isomerase | 17.58 | 1 | 0.81372 | 0.004402 | 0.7755 | 0.002 | 0.953 | 0.186 |
| S4PKL0 | Protein lin-7-like protein | 14.16 | 1 | 0.81305 | 0.015504 | 0.787 | 0.0012 | 0.968 | 0.385 |
| Q1HPY3 | Secreted protein acidic and rich in cysteine | 15.14 | 4 | 0.81232 | 0.038229 | 0.6703 | 0.0011 | 0.8251 | 0.02 |
| Q59HV9 | Casein kinase 2 alpha subunit | 12.25 | 3 | 0.81195 | 0.120023 | 0.7458 | 0.0016 | 0.9185 | 0.397 |
| B2ZDZ0 | Carboxylesterase CarE-12 | 6.83 | 3 | 0.81192 | 0.063217 | 0.9785 | 0.7109 | 1.2051 | 0.038 |
| Q5UAQ2 | Ribosomal protein L36 | 31.09 | 5 | 0.81138 | 0.001053 | 0.673 | 0.0003 | 0.8294 | 0.001 |
| I4DIY4 | Protein disulfide isomerase | 13.5 | 1 | 0.81107 | 0.009144 | 0.754 | 0.0002 | 0.9296 | 0.077 |
| Q5UAS1 | 60S ribosomal protein L18 | 55.74 | 11 | 0.81076 | 0.001135 | 0.7049 | 0.0001 | 0.8694 | 6E-04 |
| I4DQV7 | Longitudinals lacking | 7.14 | 1 | 0.81048 | 0.033521 | 0.7222 | 0.0001 | 0.8911 | 0.091 |
| Q0ZB78 | Eukaryotic translation initiation factor 2 subunit 2 | 12.26 | 4 | 0.80953 | 0.043612 | 0.7528 | 0.0024 | 0.9299 | 0.304 |
| Q1HPK0 | Vesicle amine transport protein | 55.13 | 20 | 0.80942 | 0.10049 | 0.8338 | 0.0106 | 1.0301 | 0.733 |
| Q0N2S4 | Poly A binding protein | 35.66 | 23 | 0.80827 | 0.024037 | 0.7759 | 0.0005 | 0.9599 | 0.42 |
| Q5UAP3 | Ribosomal protein S2 | 57.09 | 8 | 0.80797 | 0.066251 | 0.6891 | 0.0004 | 0.8528 | 0.088 |
| Q5UAQ3 | Ribosomal protein L35A | 41.77 | 7 | 0.80512 | 0.013171 | 0.7297 | 0.0004 | 0.9063 | 0.071 |
| B7U9X6 | Innexin | 16.36 | 5 | 0.80449 | 0.010567 | 0.812 | 0.0213 | 1.0093 | 0.865 |
| Q5UAL7 | Ribosomal protein S26 | 31.3 | 3 | 0.80423 | 0.162629 | 0.6598 | 0.0003 | 0.8204 | 0.151 |
| C0H6R2 | Putative cuticle protein | 38.66 | 2 | 0.80361 | 0.152971 | 0.8043 | 0.0127 | 1.0008 | 0.994 |
| A7BEX8 | Adenosine deaminase related growth factor | 10.18 | 4 | 0.80343 | 2.45E-06 | 0.7526 | 0.0044 | 0.9367 | 0.108 |
| Q1HQ97 | Cleavage and polyadenylation specific factor 4 | 5.9 | 1 | 0.80319 | 0.007736 | 0.7903 | 0.0082 | 0.9839 | 0.702 |
| G6DEU2 | Putative integrator complex subunit 2 | 1.78 | 2 | 0.80291 | 0.004582 | 0.8532 | 0.007 | 1.0627 | 0.123 |
| S4PW19 | G protein-coupled receptor kinase 2 | 18.75 | 1 | 0.80262 | 0.056901 | 0.6797 | 0.0918 | 0.8469 | 0.337 |
| E7E2K2 | Saposin-like protein | 13.78 | 15 | 0.80244 | 0.089615 | 0.7602 | 0.0035 | 0.9474 | 0.555 |
| S4Q0B8 | Tsunagi | 4.88 | 1 | 0.80231 | 0.082721 | 0.8057 | 0.0104 | 1.0042 | 0.961 |
| G6CQB6 | Cuticular protein hypothetical 7 | 8.9 | 1 | 0.80076 | 0.113782 | 0.9047 | 0.1453 | 1.1298 | 0.249 |
| H9JBD3 | tRNA-splicing ligase RtcB homolog | 15.22 | 7 | 0.80021 | 0.115708 | 0.804 | 0.0013 | 1.0047 | 0.96 |
| C4MI06 | Elongation factor 1 alpha | 71.48 | 1 | 0.79935 | 0.150584 | 0.6819 | 0.0106 | 0.8531 | 0.266 |
| B0FPE7 | Hydroxypyruvate isomerase | 22.31 | 6 | 0.79888 | 0.028886 | 0.7557 | 9E-05 | 0.9459 | 0.339 |
| I6ZYK8 | Elongation factor 1a | 80.4 | 1 | 0.79684 | 0.211029 | 0.622 | 0.0043 | 0.7805 | 0.171 |
| Q1HPW4 | Eukaryotic translation initiation factor 3 subunit I | 25.23 | 7 | 0.79599 | 0.165634 | 0.7649 | 0.0002 | 0.9609 | 0.737 |
| A9XXW1 | CDC5-related protein | 5.41 | 1 | 0.79478 | 5.28E-05 | 0.5814 | 0.0004 | 0.7316 | 3E-04 |
| C0H6R0 | Putative cuticle protein | 17.72 | 1 | 0.79312 | 0.000377 | 0.8605 | 0.0125 | 1.0849 | 0.022 |
| H9J1E9 | mRNA cap guanine-N7 methyltransferase | 2.28 | 1 | 0.79284 | 6.17E-05 | 0.7166 | 0.0003 | 0.9038 | 0.002 |
| C6KI60 | Ribosomal protein S11e | 48.68 | 9 | 0.79253 | 0.150372 | 0.6132 | 0.0012 | 0.7738 | 0.109 |
| A4GHG0 | Trehalose 6-phosphate synthase | 3.15 | 1 | 0.79195 | 0.020277 | 0.7611 | 0.0109 | 0.961 | 0.499 |
| D3J6Y1 | Elongation factor 1 alpha | 60.42 | 2 | 0.79139 | 0.120252 | 0.7681 | 0.0171 | 0.9706 | 0.795 |
| S4NLM7 | 40S ribosomal protein S27 | 40.26 | 3 | 0.79037 | 0.045455 | 0.6737 | 0.0021 | 0.8524 | 0.1 |
| C0H6G5 | Putative cuticle protein | 51.55 | 4 | 0.79026 | 0.01298 | 0.9827 | 0.4123 | 1.2436 | 0.005 |
| S4PCV7 | MOG interacting and ectopic P-granules protein 1 | 3.64 | 2 | 0.79017 | 0.067458 | 0.8223 | 0.0224 | 1.0407 | 0.635 |
| H9J271 | Elongation factor 1-alpha | 63.07 | 2 | 0.79004 | 0.000142 | 0.7145 | 0.004 | 0.9044 | 0.051 |
| S4PAY6 | Dullard | 6.38 | 1 | 0.78959 | 0.162177 | 0.9412 | 0.5175 | 1.192 | 0.199 |
| G6D5W9 | Argonaute 1 | 6.05 | 4 | 0.78952 | 0.003148 | 0.7845 | 0.0062 | 0.9937 | 0.877 |
| I4DM11 | Cuticular protein PpolCPT1 | 4.8 | 1 | 0.7888 | 0.571718 | 0.5339 | 0.2826 | 0.6768 | 0.46 |
| H9JMU2 | Protein BCCIP homolog | 11.24 | 2 | 0.78819 | 0.000798 | 0.7488 | 0.0776 | 0.9501 | 0.625 |
| Q684K3 | Translation initiation factor 2 gamma subunit | 28.85 | 10 | 0.7874 | 0.055075 | 0.6669 | 0.0029 | 0.847 | 0.117 |
| S4P782 | THO complex subunit 2 | 1.34 | 2 | 0.78703 | 0.009393 | 0.6933 | 0.0043 | 0.8809 | 0.082 |
| A6YF40 | Elongation factor 1-alpha | 52.7 | 1 | 0.78666 | 0.274216 | 0.6222 | 0.0018 | 0.7909 | 0.261 |
| G6CZ58 | Fmr | 6.8 | 1 | 0.78491 | 0.062638 | 0.665 | 0.003 | 0.8473 | 0.11 |
| G6CMY2 | PHD-finger 5A | 16.36 | 2 | 0.78397 | 0.012851 | 0.7168 | 0.0052 | 0.9144 | 0.055 |
| G6D0C6 | DNA ligase | 0.98 | 1 | 0.78367 | 0.151471 | 0.6975 | 0.0036 | 0.89 | 0.361 |
| Q59HV8 | Casein kinase 2 beta subunit | 28.64 | 4 | 0.78365 | 0.00787 | 0.7331 | 0.0008 | 0.9355 | 0.153 |
| G6DES6 | Apolipophorins | 1.12 | 1 | 0.78357 | 0.049535 | 0.7038 | 0.0018 | 0.8982 | 0.231 |
| B9X1G5 | Histone H2B | 59.68 | 10 | 0.78347 | 0.067853 | 0.832 | 0.018 | 1.0619 | 0.503 |
| Q5G1P2 | N-ethylmaleimide sensitive fusion protein | 8.88 | 1 | 0.78218 | 0.014201 | 0.9801 | 0.6022 | 1.2531 | 6E-04 |
| Q86M26 | Translation elongation factor 2 | 41.23 | 2 | 0.78072 | 0.032054 | 0.7528 | 0.0098 | 0.9643 | 0.621 |
| S4PFR9 | Armadillo | 12.2 | 1 | 0.78043 | 0.039326 | 0.8163 | 0.0163 | 1.046 | 0.555 |
| E7DZ11 | Ribosomal protein S25 | 37.82 | 4 | 0.77986 | 0.012762 | 0.6855 | 0.0011 | 0.879 | 0.063 |
| Q5R1P7 | Heat shock protein hsp 19.9 | 75.71 | 6 | 0.77978 | 0.006711 | 0.8111 | 0.0172 | 1.0401 | 0.468 |
| S4PT66 | Double-stranded RNA-binding zinc finger protein JAZ | 2.14 | 1 | 0.77846 | 0.008753 | 0.732 | 0.0002 | 0.9403 | 0.189 |
| B8XWD7 | Death-related protein | 41.58 | 2 | 0.77677 | 0.21154 | 0.8427 | 0.0871 | 1.0849 | 0.608 |
| Q03383 | Antichymotrypsin-1 | 29.25 | 2 | 0.77663 | 0.010541 | 0.7886 | 0.006 | 1.0154 | 0.705 |
| D7UT11 | DICER-2 | 11.09 | 15 | 0.77537 | 0.034224 | 0.7098 | 2E-05 | 0.9155 | 0.236 |
| Q5UAS5 | Ribosomal protein L13A | 26.47 | 5 | 0.77472 | 0.00736 | 0.6721 | 9E-05 | 0.8676 | 0.016 |
| G1UIS8 | Apolipophorin protein | 53.03 | 10 | 0.77455 | 0.002471 | 0.8239 | 0.0038 | 1.0637 | 0.091 |
| Q5UAQ0 | Ribosomal protein L37 | 38.71 | 5 | 0.77378 | 0.263885 | 0.6384 | 0.0118 | 0.8251 | 0.38 |
| Q08J22 | Superoxide dismutase [Cu-Zn] | 59.88 | 8 | 0.77294 | 0.002103 | 1.7082 | 0.0027 | 2.21 | 1E-04 |
| Q2F5L3 | Serine hydroxymethyltransferase | 9.46 | 3 | 0.77286 | 0.000248 | 0.875 | 0.014 | 1.1321 | 0.007 |
| G6CYH2 | Putative GTPase activating protein and VPS9 domains 1 isoform 1 | 0.69 | 1 | 0.77233 | 0.037953 | 0.7643 | 0.0267 | 0.9896 | 0.909 |
| D1LYM4 | Ribosomal protein L8 | 68.09 | 1 | 0.77202 | 0.121546 | 0.6739 | 0.0004 | 0.8729 | 0.305 |
| H9JQ10 | DNA topoisomerase 2 | 3 | 4 | 0.77193 | 0.019761 | 0.7481 | 0.0017 | 0.9692 | 0.6 |
| M1Q038 | Nonvisual arrestin | 3.41 | 1 | 0.77189 | 0.007869 | 0.9044 | 0.0156 | 1.1716 | 0.011 |
| O76190 | 60S ribosomal protein L5 | 50.5 | 18 | 0.77165 | 0.010075 | 0.6433 | 0.0004 | 0.8337 | 0.02 |
| B2CN41 | Ribosomal protein S5 | 36.45 | 1 | 0.77153 | 0.030214 | 0.5724 | 0.0032 | 0.7419 | 0.002 |
| D7EYI5 | Elongation factor 1-alpha | 43.22 | 2 | 0.77066 | 0.039534 | 0.7345 | 0.0388 | 0.953 | 0.661 |
| Q2LB51 | Elongation factor 1-alpha | 38.74 | 1 | 0.76992 | 0.055718 | 0.7155 | 0.0009 | 0.9294 | 0.391 |
| Q5UAN4 | Ribosomal protein S10 | 74.38 | 16 | 0.76932 | 0.029592 | 0.6746 | 2E-05 | 0.8769 | 0.107 |
| S4PW21 | Heterogeneous nuclear ribonucleoprotein L | 11.84 | 1 | 0.76905 | 0.182412 | 0.7382 | 0.0088 | 0.9599 | 0.788 |
| D1LYQ9 | Ribosomal protein S15A | 61.11 | 9 | 0.76794 | 0.036769 | 0.6526 | 0.0026 | 0.8498 | 0.116 |
| C7AQZ4 | DnaJ-6 | 2.87 | 1 | 0.76736 | 0.337679 | 0.8059 | 0.3835 | 1.0502 | 0.578 |
| B9A9Z3 | Histone H2A | 37.1 | 2 | 0.76732 | 0.008208 | 0.7882 | 0.0139 | 1.0272 | 0.591 |
| Q2F6B2 | Dynactin 4 protein | 13.26 | 2 | 0.76629 | 0.006433 | 0.8345 | 0.0036 | 1.089 | 0.082 |
| G6CU73 | Transketolase | 14.47 | 1 | 0.76621 | 0.033611 | 0.7473 | 0.0152 | 0.9753 | 0.729 |
| C0H6J6 | Putative cuticle protein | 44.04 | 3 | 0.76576 | 0.104458 | 0.7727 | 0.1056 | 1.0091 | 0.874 |
| I4DIM3 | Ribosomal protein L23 | 51.43 | 6 | 0.76529 | 0.011043 | 0.6719 | 0.0001 | 0.8779 | 0.041 |
| I4DRR2 | Splicing factor pTSR1 | 10.86 | 2 | 0.76442 | 0.003 | 0.694 | 0.0034 | 0.9079 | 0.099 |
| S4PYA6 | Thioredoxin domain-containing protein 1 | 4.42 | 1 | 0.76341 | 0.506136 | 0.5949 | 0.1947 | 0.7793 | 0.536 |
| I4DRV4 | 26S proteasome non-ATPase regulatory subunit rpn12 | 15.57 | 1 | 0.76203 | 0.036471 | 0.8022 | 0.0055 | 1.0528 | 0.449 |
| H9JRG6 | Eukaryotic translation initiation factor 3 subunit L | 7.94 | 4 | 0.76064 | 0.084289 | 0.6466 | 0.0011 | 0.85 | 0.206 |
| U6C391 | Cytochrome c oxidase subunit 1 | 3 | 1 | 0.76002 | 0.056534 | 0.6733 | 0.012 | 0.8859 | 0.132 |
| S4NN45 | Zinc finger protein hangover | 5.1 | 1 | 0.75954 | 0.00981 | 0.7617 | 0.0192 | 1.0028 | 0.964 |
| C0H6L7 | Putative cuticle protein | 61.02 | 6 | 0.7595 | 0.00083 | 0.7264 | 0.0019 | 0.9564 | 0.126 |
| Q5UAS4 | Ribosomal protein L14 | 40.61 | 7 | 0.75949 | 0.022589 | 0.6528 | 0.0007 | 0.8595 | 0.084 |
| S4P9G1 | Zinc finger protein 207 | 5.94 | 1 | 0.75942 | 0.021228 | 0.7191 | 0.001 | 0.947 | 0.415 |
| D1LYM3 | Ribosomal protein L31 | 48.39 | 7 | 0.75861 | 4.12E-05 | 0.6384 | 0.0004 | 0.8415 | 0.002 |
| H6A2V0 | Translation elongation factor 1-alpha | 51.04 | 1 | 0.75846 | 0.026031 | 0.6139 | 0.003 | 0.8094 | 0.061 |
| Q1HQB2 | Septin | 5.8 | 1 | 0.75819 | 0.007649 | 0.8609 | 0.1219 | 1.1354 | 0.146 |
| S4P6P4 | Bitesize | 1.44 | 1 | 0.75817 | 0.020687 | 0.7831 | 0.0362 | 1.0329 | 0.707 |
| I4DJA9 | Myosin light chain cytoplasmic | 37.41 | 5 | 0.7571 | 0.002054 | 0.7908 | 0.0204 | 1.0445 | 0.44 |
| E3UKP3 | 40S ribosomal protein S24 | 47.73 | 7 | 0.756 | 0.070777 | 0.6454 | 0.0001 | 0.8537 | 0.183 |
| Q1HQ28 | DEAD box polypeptide 5 isoform 2 | 11.52 | 1 | 0.75506 | 0.093558 | 0.7311 | 0.0144 | 0.9682 | 0.798 |
| S4PGW4 | Small nuclear ribonucleoprotein sm d2 | 32.77 | 1 | 0.75282 | 0.041434 | 0.7719 | 0.0078 | 1.0254 | 0.772 |
| S4P5E6 | U2 small nuclear ribonucleoprotein auxiliary factor 2 | 11.56 | 1 | 0.75217 | 0.139447 | 0.7829 | 0.1759 | 1.0409 | 0.641 |
| Q5UAS6 | 60S ribosomal protein L13 | 30.45 | 8 | 0.75062 | 0.036604 | 0.6442 | 2E-05 | 0.8582 | 0.119 |
| C3PPG3 | 40S ribosomal protein S13 | 58.28 | 10 | 0.74975 | 0.010996 | 0.6238 | 1E-05 | 0.832 | 0.021 |
| Q5UAR3 | Ribosomal protein L26 | 35.14 | 6 | 0.74973 | 0.040564 | 0.6437 | 0.0045 | 0.8586 | 0.17 |
| Q962T5 | 60S ribosomal protein L24 | 34.84 | 1 | 0.7497 | 0.01217 | 0.7866 | 0.014 | 1.0493 | 0.48 |
| B4Y6M2 | Elongation factor-1 alpha | 43.5 | 2 | 0.74862 | 0.124944 | 0.6974 | 0.0008 | 0.9315 | 0.613 |
| G6D562 | Eukaryotic translation initiation factor 3 subunit F | 2.17 | 1 | 0.74849 | 0.231342 | 0.6922 | 0.2078 | 0.9248 | 0.526 |
| Q8WPG9 | Annexin | 43.3 | 1 | 0.74848 | 0.003371 | 0.66 | 0.0039 | 0.8818 | 0.045 |
| G6CJ69 | Eukaryotic initiation factor 4A-III | 20.05 | 4 | 0.74707 | 0.059073 | 0.6559 | 0.004 | 0.878 | 0.284 |
| H9J5J4 | Protein CLP1 homolog | 2.76 | 1 | 0.7457 | 0.126848 | 0.7659 | 0.021 | 1.0271 | 0.825 |
| S4PXM6 | DNA replication licensing factor Mcm6 | 4.35 | 1 | 0.74529 | 0.323275 | 0.7147 | 0.0091 | 0.9589 | 0.864 |
| G9JLN1 | Nep1 protein | 5.08 | 1 | 0.74523 | 0.115209 | 0.6161 | 0.0008 | 0.8267 | 0.231 |
| G6CPN3 | Protein disulfide-isomerase like protein ERp57 | 13.76 | 1 | 0.74486 | 0.093913 | 0.7017 | 0.001 | 0.9421 | 0.624 |
| I4DJ37 | Similar to CG12947 | 5.2 | 1 | 0.7441 | 0.218404 | 0.9141 | 0.4273 | 1.2285 | 0.302 |
| Q5UAR7 | Ribosomal protein L22 | 42.86 | 6 | 0.74409 | 0.110344 | 0.6622 | 0.0052 | 0.89 | 0.437 |
| Q5UAR0 | Ribosomal protein L28 | 26.62 | 7 | 0.74405 | 0.033187 | 0.6209 | 0.0015 | 0.8345 | 0.104 |
| I4DKY6 | Similar to CG9705 | 10.85 | 1 | 0.74287 | 0.060243 | 0.8276 | 0.1691 | 1.1141 | 0.434 |
| H9J4Z5 | Vitellogenin | 42.87 | 7 | 0.74213 | 0.012142 | 0.9224 | 0.0229 | 1.2429 | 0.01 |
| Q5UAT4 | Ribosomal protein L7 | 49.07 | 17 | 0.74085 | 0.060209 | 0.6279 | 0.0008 | 0.8475 | 0.187 |
| Q1HDZ7 | CG15440-like protein | 7.48 | 2 | 0.7401 | 0.114116 | 0.6947 | 0.0073 | 0.9387 | 0.661 |
| S4PYF8 | Oxysterol binding protein | 2.61 | 1 | 0.7399 | 0.023622 | 0.7366 | 0.0008 | 0.9956 | 0.95 |
| D2CVL0 | GTP-binding nuclear protein ran | 39.91 | 1 | 0.73983 | 0.083109 | 0.6964 | 0.0003 | 0.9413 | 0.615 |
| Q60FC4 | Homeodomain protein PBX | 5.69 | 1 | 0.73977 | 0.001235 | 0.7175 | 0.0029 | 0.9699 | 0.457 |
| C0H6H0 | Putative cuticle protein | 4.12 | 1 | 0.73933 | 0.083383 | 1.2245 | 0.0682 | 1.6563 | 0.008 |
| U6JP65 | Histone H1 | 21.16 | 1 | 0.73899 | 0.020256 | 0.6615 | 0.0008 | 0.8952 | 0.185 |
| Q6PYX4 | Innexin | 12.81 | 4 | 0.73894 | 0.000709 | 0.7269 | 4E-05 | 0.9837 | 0.435 |
| Q5UAP2 | Ribosomal protein S3 | 63.79 | 15 | 0.73856 | 0.011043 | 0.6078 | 0.0004 | 0.8229 | 0.03 |
| Q5UAN7 | Ribosomal protein S7 | 70 | 15 | 0.73458 | 0.015006 | 0.6078 | 1E-05 | 0.8274 | 0.036 |
| B3TH81 | Ribosomal protein S2 | 51.47 | 1 | 0.73417 | 0.000285 | 0.6151 | 0.0003 | 0.8378 | 0.002 |
| Q5UAS2 | 60S ribosomal protein L17 | 30.48 | 6 | 0.73384 | 0.024112 | 0.6042 | 0.0005 | 0.8234 | 0.067 |
| B7U2V4 | Transport protein Sec61 alpha subunit | 10.5 | 5 | 0.73208 | 0.00096 | 0.6729 | 0.0003 | 0.9192 | 0.01 |
| S4P7V2 | Ubl carboxyl-terminal hydrolase | 11.11 | 1 | 0.73207 | 0.119267 | 0.7932 | 0.1261 | 1.0835 | 0.462 |
| T2AV05 | Ribosomal protein S5 | 39.36 | 1 | 0.7316 | 0.034729 | 0.6618 | 0.007 | 0.9046 | 0.186 |
| S4PZZ7 | MOF protein | 13.89 | 1 | 0.73119 | 0.037642 | 0.7346 | 0.0217 | 1.0047 | 0.939 |
| Q5MXS2 | Elongation factor 1-alpha | 36.87 | 2 | 0.73058 | 0.075531 | 0.6664 | 0.0021 | 0.9122 | 0.476 |
| S4PF17 | Glutamate carboxypeptidase | 9.58 | 1 | 0.72973 | 0.030178 | 0.6742 | 0.0045 | 0.9239 | 0.293 |
| H9J3H2 | Peptidyl-prolyl cis-trans isomerase | 57 | 9 | 0.72735 | 0.020473 | 0.7584 | 0.0001 | 1.0427 | 0.541 |
| D1LYN7 | Ribosomal protein L35 | 23.58 | 4 | 0.72636 | 0.129148 | 0.5499 | 0.0009 | 0.757 | 0.168 |
| C4PG27 | Receptor for activated protein kinase C | 66.14 | 15 | 0.72544 | 0.016191 | 0.621 | 0.0001 | 0.856 | 0.08 |
| Q1HQB3 | Syndecan binding protein | 12.94 | 2 | 0.72441 | 0.003556 | 0.8074 | 0.0294 | 1.1146 | 0.141 |
| Q5UAU0 | Ribosomal protein P1 | 76.79 | 1 | 0.72434 | 0.014251 | 0.6019 | 0.0003 | 0.831 | 0.045 |
| A4UWI6 | Elongation factor 1 alpha | 53.99 | 2 | 0.72317 | 0.028643 | 0.5866 | 0.0003 | 0.8112 | 0.068 |
| Q2F687 | Histone H3 | 37.5 | 3 | 0.7221 | 0.133343 | 0.759 | 0.0061 | 1.0511 | 0.75 |
| H9IYR0 | Methionine aminopeptidase | 5.82 | 2 | 0.72135 | 0.000663 | 0.5669 | 0.0003 | 0.7859 | 7E-04 |
| Q5UAR9 | Ribosomal protein L19 | 44 | 10 | 0.7196 | 0.038925 | 0.5488 | 7E-05 | 0.7626 | 0.053 |
| H9JKZ8 | Peptidyl-prolyl cis-trans isomerase | 4.93 | 1 | 0.7177 | 0.169843 | 0.6599 | 0.0027 | 0.9194 | 0.667 |
| Q1HQ47 | Isocitrate dehydrogenase | 49.43 | 17 | 0.71717 | 0.013686 | 0.6352 | 6E-05 | 0.8857 | 0.128 |
| H9JPL2 | 60S ribosomal protein L18a | 53.11 | 11 | 0.71681 | 0.010295 | 0.6401 | 0.0007 | 0.8929 | 0.146 |
| Q6F467 | Ribosomal protein S17 | 43.51 | 7 | 0.71534 | 0.001688 | 0.58 | 0.0012 | 0.8108 | 0.016 |
| Q5UAT1 | Ribosomal protein L9 | 63.68 | 12 | 0.71483 | 0.002415 | 0.625 | 0.0006 | 0.8743 | 0.039 |
| E9JEG5 | Acinus | 4.06 | 3 | 0.71438 | 0.086949 | 0.7277 | 0.0006 | 1.0186 | 0.888 |
| Q9BPS1 | Elongation factor 1 delta | 27.1 | 6 | 0.71399 | 0.056784 | 0.5174 | 2E-05 | 0.7247 | 0.054 |
| Q5UAR2 | 60S ribosomal protein L27 | 29.85 | 5 | 0.71382 | 0.014359 | 0.5862 | 7E-05 | 0.8212 | 0.042 |
| S4NXU3 | Zipper | 5.23 | 1 | 0.71297 | 0.045482 | 0.7308 | 0.0024 | 1.025 | 0.81 |
| C0H6M4 | Putative cuticle protein | 20.13 | 1 | 0.71187 | 0.007375 | 0.6752 | 0.0074 | 0.9484 | 0.417 |
| E9JEH7 | Gas2 | 1.4 | 1 | 0.71114 | 0.026139 | 0.6527 | 0.0101 | 0.9179 | 0.459 |
| Q2QEH2 | Cellular retinoic acid binding protein | 68.18 | 10 | 0.71105 | 0.003119 | 0.7529 | 0.0033 | 1.0589 | 0.277 |
| Q8T104 | Projectin-like protein | 1.4 | 2 | 0.70979 | 0.001967 | 0.7653 | 0.0091 | 1.0782 | 0.209 |
| A8CGN6 | Nicotinic acetylcholine receptor subunit alpha 9 | 3.11 | 1 | 0.70957 | 0.016148 | 0.7332 | 0.0343 | 1.0334 | 0.753 |
| G6CSN9 | Hexokinase | 9.07 | 1 | 0.70921 | 0.094629 | 0.8509 | 0.1393 | 1.1998 | 0.149 |
| G6CUC3 | Protein kinase C | 3.58 | 1 | 0.70879 | 0.01789 | 0.9803 | 0.9183 | 1.3831 | 0.134 |
| S4P7T1 | HEM-protein | 4.45 | 2 | 0.70693 | 0.001173 | 0.7034 | 0.0028 | 0.995 | 0.914 |
| I4DMT2 | Reptin | 7.91 | 2 | 0.70604 | 0.022339 | 0.6667 | 0.0101 | 0.9443 | 0.557 |
| G6DLT7 | Kettin protein | 4.54 | 1 | 0.70575 | 0.08457 | 0.718 | 0.0217 | 1.0173 | 0.909 |
| H9B420 | Yellow-c | 32.43 | 1 | 0.70458 | 0.000246 | 0.9387 | 0.2695 | 1.3323 | 0.003 |
| Q9Y0V4 | Small zinc finger-like protein | 11.43 | 1 | 0.70397 | 0.212829 | 0.711 | 0.2224 | 1.01 | 0.615 |
| S4PDK8 | Synoviolin | 4.58 | 1 | 0.70305 | 0.008839 | 0.6454 | 0.0025 | 0.918 | 0.292 |
| Q69FX2 | Promoting protein | 16.88 | 2 | 0.70295 | 0.000477 | 0.7344 | 8E-05 | 1.0448 | 0.066 |
| Q2F639 | WD repeat domain 61 | 3.42 | 1 | 0.70282 | 0.011416 | 0.8063 | 0.0221 | 1.1472 | 0.126 |
| Q9GZJ8 | Mre11 | 5.57 | 3 | 0.70223 | 0.01971 | 0.6376 | 0.0015 | 0.908 | 0.315 |
| Q2VEW6 | SUMO | 34.07 | 3 | 0.70221 | 0.082501 | 0.7281 | 0.0007 | 1.0369 | 0.789 |
| H6VTU8 | Elongation factor 1-alpha | 39.95 | 1 | 0.6995 | 0.154381 | 0.6326 | 0.0029 | 0.9044 | 0.61 |
| I7CP30 | Cathepsin L-like protease | 9.18 | 1 | 0.69943 | 0.007787 | 0.608 | 0.0014 | 0.8692 | 0.033 |
| S4P722 | Methionyl-tRNA synthetase | 1.19 | 1 | 0.6989 | 0.074151 | 0.5898 | 0.0022 | 0.8438 | 0.302 |
| C8C2R2 | Elongation factor-1 alpha | 62.66 | 1 | 0.69823 | 0.059862 | 0.6891 | 0.0792 | 0.9869 | 0.94 |
| A7BJ65 | BmRelish1 | 2.67 | 2 | 0.69787 | 0.000796 | 0.6923 | 0.0064 | 0.992 | 0.884 |
| F0UYY2 | Thymosin isoform 1 | 31.95 | 1 | 0.69652 | 0.000729 | 0.7057 | 0.0021 | 1.0131 | 0.701 |
| O96052 | Cuticle protein LCP18 | 71.43 | 4 | 0.69547 | 0.005921 | 0.7825 | 0.0012 | 1.1251 | 0.07 |
| Q5UAM3 | Ribosomal protein S19 | 33.33 | 7 | 0.69479 | 0.009599 | 0.5421 | 0.0003 | 0.7802 | 0.025 |
| H9JUK5 | Ubiquitin carboxyl-terminal hydrolase | 0.61 | 1 | 0.69375 | 0.014192 | 0.7257 | 0.0022 | 1.046 | 0.563 |
| Q2PQU4 | 30K protein 13 | 10.32 | 3 | 0.69179 | 0.00223 | 0.697 | 0.0071 | 1.0075 | 0.897 |
| S4PLV3 | Elongation factor Tu | 16.81 | 1 | 0.69123 | 0.028212 | 0.6093 | 0.0002 | 0.8815 | 0.251 |
| I6ZYL9 | Elongation factor 1a | 70.8 | 2 | 0.69121 | 0.026936 | 0.644 | 0.0077 | 0.9317 | 0.553 |
| P83632 | 27 kDa hemolymph protein | 6.78 | 1 | 0.69056 | 0.011647 | 0.6966 | 0.0029 | 1.0087 | 0.903 |
| H9J7V0 | Eukaryotic translation initiation factor 3 subunit D | 5.28 | 3 | 0.69037 | 0.012608 | 0.6089 | 9E-05 | 0.8819 | 0.15 |
| Q2F5W2 | Suppressor of Lec15 glycosylation mutation-like protein | 4.05 | 1 | 0.68977 | 0.021709 | 0.5255 | 0.0003 | 0.7619 | 0.047 |
| A2TK63 | Nucleoplasmin-like protein | 21.74 | 2 | 0.68888 | 0.041595 | 0.7307 | 0.0146 | 1.0607 | 0.634 |
| Q53EK0 | Prolyl 4-hydroxylase alpha subunit | 6.36 | 4 | 0.68871 | 0.001189 | 0.6783 | 0.0014 | 0.9849 | 0.688 |
| G6CS45 | Ribosomal protein P0 | 38.92 | 2 | 0.68776 | 0.017575 | 0.6816 | 0.0001 | 0.9911 | 0.911 |
| I4DMT8 | Proliferation-associated 2g4 | 16.62 | 1 | 0.6862 | 0.031573 | 0.6199 | 0.0001 | 0.9034 | 0.363 |
| Q66SV4 | Ribosomal protein S23 | 52.45 | 9 | 0.68597 | 0.031665 | 0.5441 | 5E-05 | 0.7932 | 0.092 |
| C0H6Y2 | Putative cuticle protein | 1.49 | 1 | 0.68044 | 0.005743 | 0.7599 | 0.0166 | 1.1168 | 0.087 |
| G9F9F7 | Seminal fluid protein CSSFP006 | 2.35 | 1 | 0.68033 | 0.003101 | 0.7343 | 4E-05 | 1.0793 | 0.133 |
| Q0ZAL3 | Splicing factor proline-and glutamine-rich | 28.55 | 15 | 0.68002 | 0.046757 | 0.6419 | 0.0002 | 0.9439 | 0.644 |
| Q5MGK9 | Ribosomal protein S19 | 41.5 | 5 | 0.67839 | 0.092523 | 0.521 | 3E-05 | 0.768 | 0.199 |
| I4DIX4 | Ribosomal protein L24 | 40.65 | 2 | 0.67771 | 0.060802 | 0.581 | 0.0003 | 0.8573 | 0.324 |
| S4NKM2 | Putative ATP-sensitive inward rectifier potassium channel 12-like protein | 15 | 1 | 0.67739 | 0.246918 | 1.1576 | 0.1994 | 1.7089 | 0.063 |
| D9N4J4 | Tudor staphylococcus/micrococcal nuclease | 29.73 | 25 | 0.67301 | 0.001914 | 0.5722 | 1E-04 | 0.8503 | 0.02 |
| Q6F482 | 60S ribosomal protein L39 | 23.53 | 2 | 0.67289 | 0.017111 | 0.5763 | 0.0082 | 0.8565 | 0.08 |
| A4PHN7 | Beta-hexosaminidase | 3.17 | 1 | 0.67265 | 0.174851 | 0.569 | 0.0036 | 0.8459 | 0.519 |
| Q4JI71 | FK506-binding protein | 27.11 | 11 | 0.6709 | 0.002905 | 0.6033 | 3E-05 | 0.8992 | 0.07 |
| L7WM53 | N-acetylgalactosaminyltransferase | 14.03 | 6 | 0.6699 | 0.000158 | 0.6249 | 0.0011 | 0.9328 | 0.136 |
| Q1HQ84 | Mitochondrial 28S ribosomal protein S25 | 8.98 | 1 | 0.66988 | 0.056644 | 0.5235 | 0.0107 | 0.7815 | 0.06 |
| D1LYQ1 | Ribosomal protein L29 | 23.29 | 3 | 0.66975 | 0.018262 | 0.4896 | 0.0002 | 0.731 | 0.028 |
| G6CTC1 | Phosphatidate cytidylyltransferase | 1.79 | 1 | 0.66907 | 0.016779 | 0.7317 | 0.0506 | 1.0936 | 0.237 |
| I4DPD8 | Translation initiation factor eif-2b | 10.7 | 1 | 0.66782 | 0.033197 | 0.7516 | 0.0036 | 1.1255 | 0.284 |
| G6D4F2 | Glutathione synthetase | 3.77 | 1 | 0.66757 | 0.027086 | 0.674 | 0.0138 | 1.0097 | 0.895 |
| Q5MGL3 | 60S ribosomal protein L38 | 50 | 4 | 0.6671 | 0.060482 | 0.5318 | 0.0003 | 0.7972 | 0.205 |
| Q2F5V9 | Signal sequence receptor | 26.84 | 3 | 0.66559 | 5.11E-05 | 0.5587 | 3E-05 | 0.8394 | 1E-04 |
| H9JQG8 | Purine nucleoside phosphorylase | 2.49 | 1 | 0.66417 | 0.078912 | 0.4454 | 8E-05 | 0.6706 | 0.092 |
| D1LYL9 | 40S ribosomal protein S12 | 62.59 | 8 | 0.66403 | 0.104967 | 0.4952 | 0.0001 | 0.7458 | 0.211 |
| H9IUP7 | Single-stranded DNA-binding protein | 7.44 | 1 | 0.66354 | 6.14E-05 | 0.6397 | 0.0009 | 0.9641 | 0.332 |
| H9IZ89 | Acyl-coenzyme A oxidase | 4.8 | 2 | 0.66281 | 0.012255 | 0.7138 | 0.0047 | 1.077 | 0.35 |
| Q308S4 | Cecropin E | 27.69 | 1 | 0.66159 | 0.000519 | 0.6552 | 0.0003 | 0.9904 | 0.705 |
| Q8WPH0 | Annexin | 27.37 | 1 | 0.66085 | 0.001513 | 0.5674 | 0.0016 | 0.8585 | 0.043 |
| F8UN44 | Heat shock protein 70-3 | 54.5 | 29 | 0.65747 | 0.01225 | 0.5942 | 2E-05 | 0.9038 | 0.267 |
| Q5UAQ6 | Ribosomal protein L32 | 49.25 | 8 | 0.65572 | 0.024432 | 0.5446 | 5E-05 | 0.8306 | 0.158 |
| A8HHY4 | Elongation factor 1 alpha | 55.45 | 2 | 0.6534 | 0.020029 | 0.6751 | 0.0301 | 1.0332 | 0.814 |
| A1E129 | Kinesin-like protein 1 | 14.63 | 1 | 0.65338 | 0.032729 | 0.653 | 0.0025 | 0.9995 | 0.996 |
| Q5UAQ5 | Ribosomal protein L34 | 35.29 | 6 | 0.65137 | 0.005166 | 0.5167 | 0.0001 | 0.7933 | 0.024 |
| Q6T9Z7 | Fibroinas | 23.46 | 8 | 0.64927 | 0.029906 | 0.5578 | 0.0001 | 0.8591 | 0.26 |
| Q5UAT7 | Ribosomal protein L4 | 52.57 | 26 | 0.64905 | 0.044701 | 0.5021 | 9E-06 | 0.7736 | 0.147 |
| Q0Q017 | Serpin-like protein | 5.41 | 1 | 0.64702 | 0.028793 | 0.6546 | 0.0013 | 1.0117 | 0.918 |
| S4Q042 | Serine/threonine-protein kinase | 4.49 | 2 | 0.64674 | 0.000101 | 0.6495 | 6E-05 | 1.0042 | 0.835 |
| D1LYL6 | Ribosomal protein S9 | 46.91 | 14 | 0.64611 | 0.014184 | 0.5123 | 1E-05 | 0.7929 | 0.066 |
| A8R081 | Bombyx homolog of P-element somatic inhibitor | 8.68 | 6 | 0.64473 | 0.001153 | 0.575 | 1E-05 | 0.8918 | 0.038 |
| Q5UAM4 | Ribosomal protein S18 | 57.24 | 12 | 0.6425 | 0.004194 | 0.4876 | 3E-05 | 0.759 | 0.01 |
| O17504 | Multiprotein bridging factor 1 | 13.7 | 3 | 0.64228 | 0.107737 | 0.5934 | 0.0019 | 0.9238 | 0.712 |
| A0ST24 | Dachshund | 6.57 | 1 | 0.64227 | 0.00252 | 0.5686 | 5E-05 | 0.8853 | 0.058 |
| Q5UAT3 | Ribosomal protein L7A | 48.88 | 9 | 0.64122 | 0.040942 | 0.48 | 4E-05 | 0.7485 | 0.118 |
| H9JBC0 | Beta-galactosidase | 3.37 | 2 | 0.64115 | 0.027354 | 0.5863 | 0.0013 | 0.9145 | 0.494 |
| A9XI30 | Glutathione S-transferase 7 | 24.89 | 2 | 0.64064 | 0.003123 | 0.5434 | 0.003 | 0.8483 | 0.042 |
| S4PR87 | Putative tRNA pseudouridine synthase Pus10 | 4.32 | 1 | 0.63999 | 0.026753 | 0.5623 | 0.002 | 0.8786 | 0.357 |
| S4PT05 | 40S ribosomal protein S14 | 61.96 | 6 | 0.63993 | 0.001153 | 0.531 | 0.0006 | 0.8297 | 0.018 |
| G6CL05 | Heme oxygenase | 5.56 | 1 | 0.6397 | 0.037567 | 0.7235 | 0.0239 | 1.131 | 0.31 |
| Q3LB95 | Chemosensory protein 7 | 26.23 | 2 | 0.63937 | 0.000204 | 0.6176 | 1E-05 | 0.9659 | 0.185 |
| Q5UAR8 | Ribosomal protein L21 | 41.51 | 4 | 0.63845 | 0.014452 | 0.4793 | 4E-05 | 0.7507 | 0.046 |
| S4NJL4 | Integrator complex subunit 3-like protein | 6.06 | 1 | 0.63828 | 0.008178 | 0.5125 | 0.0034 | 0.803 | 0.026 |
| H6VTR6 | DnaJ-26 | 4.29 | 1 | 0.63557 | 0.005907 | 0.6111 | 0.0012 | 0.9615 | 0.629 |
| H9IW70 | 40S ribosomal protein S6 | 37.94 | 1 | 0.63463 | 0.001012 | 0.4944 | 8E-05 | 0.7791 | 0.005 |
| C0H6H6 | Putative cuticle protein | 8.21 | 1 | 0.63456 | 0.048306 | 0.549 | 0.0004 | 0.8651 | 0.391 |
| G6DEL5 | Protein disulfide isomerase | 14.75 | 1 | 0.63209 | 0.010771 | 0.5536 | 0.0002 | 0.8758 | 0.198 |
| A1DYI6 | Cathepsin L-like cysteine proteinase | 8.14 | 1 | 0.63174 | 0.038342 | 0.6157 | 0.007 | 0.9747 | 0.846 |
| T1WLT1 | Cytochrome P450 CYP301A1 | 4.12 | 1 | 0.6291 | 0.010706 | 0.5865 | 0.0023 | 0.9323 | 0.461 |
| C0H6K8 | Putative cuticle protein | 32.14 | 5 | 0.62544 | 2.21E-05 | 0.6714 | 2E-05 | 1.0735 | 0.005 |
| Q2LAV7 | Elongation factor 1-alpha | 44.94 | 1 | 0.62354 | 0.065609 | 0.5294 | 0.0003 | 0.849 | 0.417 |
| S4PMB1 | Translocation associated membrane protein | 3.71 | 2 | 0.62318 | 0.050335 | 0.5535 | 0.0045 | 0.8881 | 0.525 |
| Q9XXV0 | Prophenoloxidase activating enzyme | 17.01 | 1 | 0.62251 | 0.004136 | 0.6821 | 0.0029 | 1.0958 | 0.172 |
| Q6IE02 | Mod(Mdg4)-heS00531 | 27.62 | 7 | 0.62099 | 0.004115 | 0.5828 | 0.0001 | 0.9385 | 0.375 |
| Q5UAS3 | Ribosomal protein L15 | 38.24 | 8 | 0.61756 | 0.007988 | 0.4604 | 1E-05 | 0.7455 | 0.027 |
| E3SRH1 | Will die slowly | 3.18 | 1 | 0.61721 | 0.017449 | 0.6701 | 0.0012 | 1.0857 | 0.432 |
| S4PHK7 | Cyclin L1 | 3.77 | 2 | 0.61682 | 0.008513 | 0.6471 | 0.0005 | 1.0491 | 0.556 |
| I4DPQ8 | Similar to CG8549 | 4.05 | 1 | 0.61439 | 0.001157 | 0.6337 | 0.0005 | 1.0315 | 0.538 |
| F1B185 | Elongation factor 1-alpha | 43.17 | 1 | 0.6143 | 0.052192 | 0.4934 | 0.0021 | 0.8033 | 0.277 |
| S4PE18 | Upf1 (Fragment) | 5.81 | 1 | 0.61416 | 0.094021 | 0.447 | 0.0031 | 0.7278 | 0.234 |
| H9JTG9 | Aldo-keto reductase AKR2E4 | 2.6 | 1 | 0.61215 | 0.007666 | 0.6176 | 0.0013 | 1.0089 | 0.913 |
| E5CWS7 | Histone H3 | 41.28 | 1 | 0.61192 | 0.089418 | 0.6276 | 0.0022 | 1.0256 | 0.906 |
| P42852 | Pupal cuticle protein | 40.71 | 7 | 0.60927 | 0.006216 | 0.5919 | 0.0011 | 0.9715 | 0.747 |
| I4DIR2 | Ribosomal protein L37A | 36.67 | 4 | 0.60873 | 0.072535 | 0.5114 | 9E-05 | 0.8401 | 0.434 |
| Q06FJ6 | C-type lectin | 28.9 | 3 | 0.60856 | 0.002087 | 0.6381 | 0.0054 | 1.0485 | 0.495 |
| B8YJN4 | Vasa-like protein | 6.41 | 2 | 0.60637 | 0.048263 | 0.5334 | 0.0004 | 0.8797 | 0.489 |
| I4DMI7 | Cuticular protein PpolCPG12 | 33.9 | 1 | 0.60474 | 0.01322 | 0.5042 | 0.0011 | 0.8338 | 0.118 |
| I4DPR6 | Insulin receptor tyrosine kinase substrate | 17.69 | 2 | 0.60424 | 2.44E-05 | 0.4711 | 0.0002 | 0.7797 | 0.003 |
| Q2F6C1 | Cytidine deaminase | 11.83 | 2 | 0.6041 | 0.093333 | 0.5356 | 0.0032 | 0.8867 | 0.633 |
| G6D456 | Putative U2 snrnp auxiliary factor | 17.18 | 2 | 0.59971 | 0.000893 | 0.6024 | 0.004 | 1.0044 | 0.946 |
| H9JJC0 | 30K protein 23 | 4.59 | 1 | 0.5981 | 0.000211 | 0.688 | 0.0079 | 1.1504 | 0.07 |
| C0H6P4 | Putative cuticle protein | 34.1 | 5 | 0.59686 | 0.007017 | 0.6505 | 0.0041 | 1.0898 | 0.405 |
| Q9U501 | Transcription elongation factor 1 homolog | 6.1 | 1 | 0.59631 | 0.031121 | 0.5884 | 0.0203 | 0.9867 | 0.928 |
| E9KH18 | Proteasome subunit | 17.96 | 1 | 0.5951 | 0.029423 | 0.5166 | 0.0001 | 0.868 | 0.386 |
| H9B457 | 30K protein 24 | 4.92 | 1 | 0.59203 | 0.000157 | 0.5804 | 0.0003 | 0.9804 | 0.621 |
| D7PGZ7 | Flavin-dependent monooxygenase FMO3B | 9.03 | 4 | 0.59041 | 0.011045 | 0.4614 | 0.0006 | 0.7815 | 0.106 |
| G6CNK9 | Ubiquitin-40S ribosomal protein S27a | 43.87 | 3 | 0.58434 | 0.017765 | 0.501 | 0.0001 | 0.8573 | 0.296 |
| H9JAW1 | Acyl-coenzyme A oxidase | 6.62 | 2 | 0.58364 | 0.011768 | 0.4363 | 3E-05 | 0.7475 | 0.066 |
| O97158 | Transferrin | 33.92 | 20 | 0.58199 | 0.000557 | 0.5925 | 0.0004 | 1.018 | 0.716 |
| H9ISA0 | 60S ribosome subunit biogenesis protein NIP7 homolog | 31.67 | 3 | 0.58124 | 0.009005 | 0.543 | 7E-05 | 0.9342 | 0.515 |
| B5LBF7 | Groucho protein | 4.19 | 1 | 0.58061 | 0.013462 | 0.5167 | 0.0004 | 0.8899 | 0.383 |
| I4DQU9 | Ftz transcription factor 1 | 5.43 | 1 | 0.58046 | 0.002844 | 0.6579 | 0.0049 | 1.1335 | 0.184 |
| E7DZ32 | Ribosomal protein L36A | 28.85 | 4 | 0.57878 | 0.031938 | 0.4573 | 4E-05 | 0.7901 | 0.228 |
| B0FGV8 | Carboxylesterase CarE-7 | 2.14 | 1 | 0.57791 | 0.003728 | 0.8919 | 0.3182 | 1.5433 | 0.009 |
| Q30CF6 | Elongation factor 1-alpha | 56.14 | 1 | 0.57618 | 0.007985 | 0.5717 | 0.0005 | 0.9922 | 0.939 |
| H9J859 | Fascin | 8.02 | 3 | 0.57131 | 0.001293 | 0.5733 | 9E-05 | 1.0035 | 0.945 |
| G6DA13 | Putative TPR repeat-containing protein C9orf52 | 1.11 | 1 | 0.56983 | 0.008011 | 0.6328 | 0.0068 | 1.1104 | 0.323 |
| G6CYV8 | Beta-tubulin | 34.57 | 2 | 0.56874 | 0.011356 | 0.5768 | 0.0008 | 1.0142 | 0.907 |
| I6TJM4 | Adenomatous polyposis coli | 10.42 | 1 | 0.56573 | 0.005547 | 0.5167 | 0.0052 | 0.9134 | 0.465 |
| G4XH85 | Ribosomal protein S30 | 8.59 | 1 | 0.56382 | 0.001664 | 0.5422 | 0.0008 | 0.9617 | 0.417 |
| A1YRL7 | Cationic peptide CP8 | 25.84 | 2 | 0.55647 | 0.000876 | 0.6397 | 0.0004 | 1.1495 | 0.018 |
| Q2F5Z2 | Keratinocyte-associated protein 2 | 9.92 | 2 | 0.55354 | 0.003283 | 0.6267 | 0.0004 | 1.1323 | 0.155 |
| I4DMZ4 | Cuticular protein PpolCPH2 | 11.84 | 1 | 0.5526 | 0.000731 | 0.5907 | 0.0007 | 1.0689 | 0.287 |
| B7U2V8 | Putative uncharacterized protein | 4.76 | 1 | 0.5514 | 0.058504 | 0.4026 | 1E-04 | 0.7302 | 0.273 |
| Q2F5V7 | Tetraspanin D107 | 2.51 | 1 | 0.55056 | 0.001908 | 0.5818 | 0.0006 | 1.0568 | 0.378 |
| Q2F5L5 | Transport protein Sec61 beta protein | 36.08 | 3 | 0.54823 | 0.003274 | 0.4249 | 0.0002 | 0.7751 | 0.026 |
| I4DJH1 | Tyrosine aminotransferase | 1.37 | 1 | 0.54494 | 0.003132 | 0.4952 | 0.0013 | 0.9088 | 0.32 |
| B5BSX8 | Beta-1,3-glucan recognition protein 3 | 4.09 | 2 | 0.54328 | 0.001748 | 0.6259 | 0.0019 | 1.152 | 0.124 |
| G6CRH7 | Putative brix domain-containing protein 2 | 2.28 | 1 | 0.54306 | 0.007313 | 0.5106 | 0.0004 | 0.9402 | 0.605 |
| S4P9Z1 | RRP12-like protein (Fragment) | 7.14 | 1 | 0.54132 | 0.112715 | 0.3528 | 0.0287 | 0.6518 | 0.402 |
| Q8T113 | 27 kDa glycoprotein | 24.23 | 4 | 0.53974 | 0.002658 | 0.6727 | 0.0005 | 1.2464 | 0.033 |
| J7QK11 | Glyceraldhyde-3-phosphate dehydrogenase | 40.99 | 1 | 0.53436 | 0.002058 | 0.6227 | 7E-05 | 1.1652 | 0.074 |
| B7XDF7 | Mutated ebony protein | 2.46 | 1 | 0.53021 | 0.00021 | 0.5539 | 0.0014 | 1.0447 | 0.499 |
| G6DTH6 | Putative ADP-ribosylation factor-like 2-like protein | 13.04 | 1 | 0.5295 | 0.000865 | 0.5195 | 0.0001 | 0.9811 | 0.761 |
| S4PC17 | Thioredoxin domain-containing protein 4 | 14.52 | 1 | 0.52668 | 0.011759 | 0.6781 | 0.0461 | 1.2874 | 0.097 |
| S4PMZ8 | Filamin (Fragment) | 28.17 | 1 | 0.52634 | 0.000809 | 0.5071 | 0.0004 | 0.9635 | 0.439 |
| G6CP74 | Putative bromodomain containing 3 | 0.6 | 1 | 0.52074 | 0.000984 | 0.5471 | 0.001 | 1.0506 | 0.416 |
| H9ISK7 | Adenylyl-sulfate kinase | 4.63 | 2 | 0.51409 | 0.004808 | 0.5245 | 0.0034 | 1.0203 | 0.824 |
| B0LKP4 | Glutathione S-transferase 11 | 17.03 | 3 | 0.51183 | 0.001015 | 0.5044 | 0.0001 | 0.9854 | 0.82 |
| C0H6P0 | Putative cuticle protein | 6.91 | 2 | 0.51145 | 0.019528 | 0.5762 | 0.0011 | 1.1266 | 0.486 |
| G9FL14 | DNA supercoiling factor | 19.57 | 5 | 0.50892 | 0.005495 | 0.4241 | 0.0001 | 0.8333 | 0.2 |
| C0H6X5 | Putative cuticle protein | 12.82 | 2 | 0.50637 | 0.000338 | 0.5167 | 6E-05 | 1.0203 | 0.668 |
| Q2F5Q6 | Kazal-type proteinase inhibitor | 23.96 | 2 | 0.50437 | 0.000583 | 0.5805 | 0.0006 | 1.1509 | 0.054 |
| B0ZT34 | Heat shock protein 25.4 | 12.11 | 3 | 0.5043 | 4.86E-05 | 0.5507 | 7E-05 | 1.0921 | 0.021 |
| C0H6F7 | Putative cuticle protein | 19.1 | 1 | 0.50232 | 0.000736 | 0.5078 | 0.0024 | 1.0109 | 0.914 |
| I4DIL3 | Dusky-like | 2.92 | 1 | 0.49549 | 0.011093 | 0.5075 | 0.0131 | 1.0243 | 0.892 |
| H9J8I1 | Small heat shock protein 27.4 | 3.66 | 1 | 0.48396 | 0.000222 | 0.5259 | 0.0004 | 1.0866 | 0.155 |
| B2BNZ1 | Carboxylesterase-like protein | 1.2 | 1 | 0.48389 | 0.000789 | 0.4401 | 7E-05 | 0.9096 | 0.234 |
| C8C2L5 | Elongation factor 1-alpha | 45.52 | 1 | 0.48341 | 0.011341 | 0.3114 | 0.0002 | 0.6443 | 0.084 |
| C0H6K6 | Putative cuticle protein | 26.79 | 6 | 0.48301 | 0.000134 | 0.4647 | 1E-05 | 0.9621 | 0.321 |
| Q9GV25 | TIA-1 like protein | 8.42 | 1 | 0.47343 | 0.003935 | 0.4904 | 0.0045 | 1.0359 | 0.667 |
| Q60F93 | Molting fluid carboxypeptidase A | 1.88 | 1 | 0.47031 | 0.003341 | 0.5632 | 0.0008 | 1.1976 | 0.117 |
| I4DJM9 | Tetraspan membrane protein in hair cell stereocilia ortholog | 3.14 | 1 | 0.46904 | 0.000292 | 0.555 | 0.0014 | 1.1833 | 0.044 |
| L7QMY1 | Carbamoylphosphate synthetase/aspartate transcarbamylase/dihydroorotase | 1.13 | 1 | 0.46463 | 0.000443 | 0.4452 | 0.0002 | 0.9582 | 0.56 |
| F5BZV1 | Serine protease HP21 | 2.2 | 1 | 0.45778 | 0.000681 | 0.5634 | 0.0017 | 1.2306 | 0.067 |
| S4NX37 | Putative n-sulfoglucosamine sulfohydrolase-like protein | 9.38 | 1 | 0.45704 | 0.00566 | 0.3308 | 1E-05 | 0.7237 | 0.099 |
| S4PX83 | Small nuclear ribonucleoprotein polypeptide | 36.9 | 1 | 0.45097 | 0.000368 | 0.4583 | 0.0013 | 1.0163 | 0.857 |
| C0H6M2 | Putative cuticle protein | 26.55 | 2 | 0.45059 | 0.001828 | 0.4505 | 5E-05 | 0.9998 | 0.998 |
| C0H6G3 | Putative cuticle protein | 8.82 | 1 | 0.44982 | 0.010158 | 0.5443 | 0.0054 | 1.21 | 0.231 |
| E7D007 | Serine protease 55 | 2.69 | 1 | 0.44465 | 0.000395 | 0.433 | 0.0006 | 0.9739 | 0.772 |
| C0H6X8 | Putative cuticle protein | 11.68 | 1 | 0.44353 | 0.024682 | 0.3811 | 0.0034 | 0.8592 | 0.568 |
| C3PPG7 | Similar to Ras-related protein Rab-39B | 11.71 | 1 | 0.43592 | 0.001025 | 0.5639 | 0.0059 | 1.2936 | 0.023 |
| Q966T8 | GGY cuticle protein 1 | 6.67 | 1 | 0.42795 | 0.001467 | 0.4961 | 0.0009 | 1.1593 | 0.139 |
| A9X4T2 | HMGA | 7.02 | 1 | 0.42781 | 0.022566 | 0.4095 | 0.0004 | 0.9572 | 0.869 |
| C0H6J4 | Putative cuticle protein | 26.28 | 3 | 0.4265 | 0.003344 | 0.4403 | 0.0003 | 1.0323 | 0.785 |
| I4DRR8 | Glutamine:fructose-6-phosphate aminotransferase 1 | 13.98 | 1 | 0.42134 | 0.000455 | 0.4581 | 7E-05 | 1.0871 | 0.248 |
| B2CMR7 | Elongation factor-1 alpha | 23.81 | 1 | 0.40647 | 0.000733 | 0.558 | 0.0053 | 1.3727 | 0.054 |
| Q1HPM2 | Mitochondrial phosphoenolpyruvate carboxykinase isoform 2 | 3.62 | 2 | 0.40567 | 0.003277 | 0.5006 | 0.0004 | 1.2339 | 0.174 |
| S4T709 | Vitellogenin OS | 2.11 | 1 | 0.38663 | 0.004636 | 0.6106 | 0.0057 | 1.5793 | 0.04 |
| U5KFU0 | Fatty-acyl CoA reductase 6 | 4.91 | 1 | 0.38559 | 0.001898 | 0.4601 | 0.0002 | 1.1933 | 0.207 |
| Q06343 | Basic juvenile hormone-suppressible protein 2 | 2.14 | 2 | 0.37935 | 0.000103 | 0.3526 | 0.0001 | 0.9295 | 0.354 |
| D7PGZ4 | Flavin-dependent monooxygenase FMO1B | 1.72 | 1 | 0.37737 | 0.000842 | 0.3906 | 5E-05 | 1.035 | 0.741 |
| D0VEN3 | Putative cuticle protein CPG38 | 17.07 | 3 | 0.37559 | 0.000108 | 0.3823 | 0.0005 | 1.0179 | 0.798 |
| H9IZN1 | Acyl-coenzyme A oxidase | 6.88 | 3 | 0.36873 | 0.002685 | 0.3327 | 0.0006 | 0.9023 | 0.583 |
| L7QZN6 | Tetrahydrofolate synthase | 9.6 | 1 | 0.36586 | 0.00012 | 0.4261 | 0.0006 | 1.1646 | 0.094 |
| G6DBV4 | Elongation of very long chain fatty acids protein | 2.42 | 1 | 0.36345 | 0.004167 | 0.264 | 2E-05 | 0.7264 | 0.191 |
| C0H6Y6 | Putative cuticle protein | 8.64 | 1 | 0.36117 | 0.000685 | 0.3884 | 0.0002 | 1.0755 | 0.428 |
| Q1HPP4 | Arylphorin | 21.76 | 14 | 0.3606 | 7.37E-06 | 0.3565 | 4E-06 | 0.9886 | 0.682 |
| C0H6H3 | Putative cuticle protein | 51 | 2 | 0.36052 | 0.000237 | 0.3771 | 0.0008 | 1.0459 | 0.604 |
| C0H6I7 | Putative cuticle protein | 61.9 | 6 | 0.35697 | 0.000413 | 0.4051 | 8E-05 | 1.1347 | 0.192 |
| K7QTQ2 | Sericin 1 | 8.18 | 1 | 0.35608 | 6.98E-05 | 0.3678 | 0.0002 | 1.0329 | 0.67 |
| I4DN78 | Glucose dehydrogenase | 1.32 | 1 | 0.35357 | 3.27E-05 | 0.3092 | 6E-05 | 0.8744 | 0.038 |
| Q3LB94 | Antennal-specific protein 3c | 15.97 | 2 | 0.3504 | 0.000119 | 0.3473 | 0.0003 | 0.991 | 0.923 |
| G3DT16 | Immune-related protein 2 | 60.24 | 7 | 0.34631 | 0.000222 | 0.3517 | 7E-06 | 1.0156 | 0.834 |
| C0H6W9 | Putative cuticle protein | 7 | 2 | 0.34125 | 0.000883 | 0.3288 | 6E-05 | 0.9637 | 0.776 |
| Q7Z1E3 | Glutathione S-transferase 2 | 8.25 | 1 | 0.33259 | 0.001162 | 0.3357 | 0.0005 | 1.0092 | 0.948 |
| A4ZXT1 | Elongation factor 1 alpha | 42.12 | 1 | 0.32942 | 0.00022 | 0.3316 | 0.0005 | 1.0065 | 0.948 |
| P09338 | Low molecular 30 kDa lipoprotein PBMHPC-23 | 63.64 | 4 | 0.32483 | 0.003397 | 0.3599 | 1E-04 | 1.1078 | 0.586 |
| C0H6R3 | Putative cuticle protein | 17.02 | 3 | 0.32453 | 0.007779 | 0.3797 | 1E-05 | 1.1699 | 0.536 |
| G6DK46 | Cuticular protein RR-2 motif 130 | 7.65 | 1 | 0.31774 | 3.5E-05 | 0.3725 | 4E-06 | 1.1724 | 0.015 |
| P48816 | Lysozyme | 7.3 | 1 | 0.31345 | 6.89E-07 | 0.4064 | 0.0002 | 1.2966 | 0.014 |
| C0H6Y5 | Putative cuticle protein | 3.13 | 1 | 0.31331 | 0.000154 | 0.3762 | 8E-05 | 1.2006 | 0.066 |
| D2X2F7 | C-type lectin 11 | 69.23 | 9 | 0.30641 | 0.000291 | 0.3097 | 3E-05 | 1.0106 | 0.918 |
| D4QGC0 | Putative uncharacterized protein 19G1P | 47.68 | 5 | 0.29768 | 1.45E-05 | 0.3238 | 8E-06 | 1.0877 | 0.02 |
| D0VEM3 | Putative cuticle protein CPR150 | 32.16 | 4 | 0.29131 | 5.58E-06 | 0.2812 | 2E-05 | 0.9652 | 0.435 |
| H9B436 | 30K protein 3 | 26.67 | 3 | 0.28711 | 8.75E-07 | 0.2945 | 9E-07 | 1.0256 | 0.053 |
| D0VEM6 | Putative cuticle protein CPG31 | 14.48 | 4 | 0.28664 | 0.000278 | 0.3101 | 2E-06 | 1.0818 | 0.452 |
| C0H6D9 | Putative cuticle protein | 61.3 | 9 | 0.28463 | 2.37E-05 | 0.3147 | 5E-05 | 1.1056 | 0.192 |
| C0H6D6 | Putative cuticle protein | 21.05 | 6 | 0.28362 | 0.000233 | 0.2773 | 1E-07 | 0.9778 | 0.823 |
| D4QGB9 | Putative uncharacterized protein 19G1Q | 52.74 | 5 | 0.28326 | 4.25E-05 | 0.295 | 0.0002 | 1.0416 | 0.651 |
| D0VEQ2 | Putative cuticle protein CPH41 | 3.29 | 4 | 0.28222 | 0.00027 | 0.3083 | 2E-07 | 1.0926 | 0.4 |
| C0H6U6 | Putative cuticle protein | 10.36 | 2 | 0.27991 | 0.000436 | 0.3464 | 0.0002 | 1.2375 | 0.16 |
| C0H6C4 | Putative cuticle protein | 16.67 | 2 | 0.26502 | 0.000114 | 0.2674 | 2E-05 | 1.0091 | 0.907 |
| H9J4G4 | 30K protein 5 | 2.79 | 1 | 0.26091 | 0.000815 | 0.3109 | 1E-04 | 1.1914 | 0.288 |
| Q2F5Q5 | Lectin 4 C-type lectin | 32.74 | 6 | 0.24979 | 4.4E-05 | 0.2377 | 1E-05 | 0.9515 | 0.494 |
| C0H6C6 | Putative cuticle protein | 27 | 3 | 0.24361 | 1.55E-05 | 0.2762 | 5E-05 | 1.1337 | 0.048 |
| C0H6D0 | Putative cuticle protein | 8.96 | 1 | 0.24303 | 1.18E-05 | 0.2531 | 3E-05 | 1.0413 | 0.589 |
| G6CJP6 | Chitin deacetylase 4 | 2.77 | 1 | 0.2416 | 0.000103 | 0.2506 | 4E-05 | 1.0374 | 0.636 |
| D0VEP7 | Putative cuticle protein CPH36 | 29.03 | 4 | 0.23733 | 6.42E-06 | 0.2465 | 5E-06 | 1.0386 | 0.411 |
| C0H6E2 | Putative cuticle protein | 30 | 2 | 0.23713 | 0.000114 | 0.244 | 0.0001 | 1.0291 | 0.813 |
| D0VEQ3 | Putative cuticle protein CPH42 | 29.32 | 3 | 0.23165 | 3.84E-05 | 0.2527 | 1E-05 | 1.0909 | 0.256 |
| C0H6F1 | Putative cuticle protein | 23.85 | 8 | 0.22896 | 1.95E-05 | 0.2422 | 2E-05 | 1.058 | 0.452 |
| C0H6Q4 | Putative cuticle protein | 1.57 | 1 | 0.22663 | 0.000176 | 0.3208 | 0.0002 | 1.4156 | 0.041 |
| Q9U4Z0 | Putative uncharacterized protein | 17.93 | 1 | 0.22521 | 0.000107 | 0.2239 | 7E-06 | 0.9944 | 0.958 |
| I4DIB8 | Cuticular protein PxutCPR34 | 26.4 | 1 | 0.22067 | 0.000532 | 0.323 | 0.0001 | 1.4636 | 0.052 |
| C0H6P9 | Putative cuticle protein | 16.86 | 4 | 0.19371 | 6.84E-06 | 0.1968 | 2E-05 | 1.016 | 0.847 |
| D0VEN6 | Putative cuticle protein CPG41 | 12.8 | 2 | 0.19184 | 0.000506 | 0.1892 | 4E-05 | 0.986 | 0.942 |
| I4DIE5 | Cuticular protein PxutCPG13 | 26.05 | 1 | 0.19114 | 0.000253 | 0.2551 | 9E-05 | 1.3346 | 0.139 |
| B6DXB0 | Osiris 9 | 5 | 1 | 0.18943 | 0.002096 | 0.3039 | 0.0008 | 1.6043 | 0.17 |
| C0H6X7 | Putative cuticle protein | 48.86 | 7 | 0.18454 | 1.52E-05 | 0.1987 | 1E-05 | 1.0768 | 0.189 |
| C0H6K1 | Putative cuticle protein | 3.2 | 1 | 0.18296 | 0.000704 | 0.1795 | 9E-07 | 0.9809 | 0.94 |
| P09334 | Low molecular 30 kDa lipoprotein PBMHP-6 | 43.75 | 11 | 0.16097 | 8.6E-06 | 0.1688 | 2E-06 | 1.0484 | 0.52 |
| C0H6M6 | Putative cuticle protein | 30.43 | 2 | 0.16073 | 3.55E-07 | 0.1794 | 3E-06 | 1.1165 | 0.072 |
| G6D1J8 | Putative sugar transporter protein 5 | 10.89 | 1 | 0.15929 | 5.12E-05 | 0.2079 | 0.0004 | 1.3055 | 0.287 |
| Q00801 | Low molecular mass 30 kDa lipoprotein 21G1 | 47.91 | 6 | 0.15457 | 4.93E-06 | 0.1571 | 2E-06 | 1.0163 | 0.734 |
| I4DIB7 | Cuticular protein PxutCPG11 | 15.99 | 1 | 0.14618 | 0.000109 | 0.1721 | 1E-05 | 1.1776 | 0.3 |
| C0H6S5 | Putative cuticle protein | 21.83 | 2 | 0.13431 | 1.01E-05 | 0.1458 | 2E-05 | 1.0859 | 0.389 |
| C0H6E7 | Putative cuticle protein | 27.67 | 3 | 0.13181 | 8.86E-06 | 0.1708 | 8E-06 | 1.2961 | 0.048 |
| C0H6R6 | Putative cuticle protein | 8.59 | 1 | 0.12777 | 0.00036 | 0.128 | 3E-05 | 1.0015 | 0.996 |
| G9I6Y1 | Arylphorin | 7.04 | 5 | 0.12748 | 6.6E-07 | 0.159 | 4E-05 | 1.2474 | 0.15 |
| I4DIG3 | Osiris 9B | 4.31 | 1 | 0.12675 | 0.000284 | 0.1103 | 4E-05 | 0.8703 | 0.635 |
| B6DXA9 | Osiris 18 | 7.04 | 2 | 0.1212 | 0.000774 | 0.1104 | 0.0006 | 0.911 | 0.806 |
| C0H6E0 | Putative cuticle protein | 49.46 | 8 | 0.11932 | 1.9E-06 | 0.1148 | 2E-06 | 0.9622 | 0.629 |
| Q1HQ95 | Cellular retinaldehyde-binding protein | 3.21 | 1 | 0.11789 | 0.000214 | 0.1075 | 1E-05 | 0.9115 | 0.772 |
| C0H6Z3 | Putative cuticle protein | 26.1 | 3 | 0.11412 | 1.34E-05 | 0.1257 | 6E-06 | 1.1016 | 0.332 |
| U3KV76 | Arylphorin | 5.73 | 2 | 0.11397 | 0.000281 | 0.1181 | 0.0003 | 1.0359 | 0.839 |

**Table S2 List of primers for qPCR and RNAi analysis**

| **Gene** | **Forward primer (5′–3′)** | **Reverse primer (5′–3′)** |
| --- | --- | --- |
| Desat | gggaactttggtattacag | tcgtagcattatgagggtctg |
| FAR | agaaacttgcgtattcgtgtc | ttagtttgaccgaagcggctgag |
| FATP | atagtggacattgaaggcaca | tcttcaactcctaagggcacgt |
| ACBP | tgaccaagccgcagccaacgt | tgaggccgatggaagctatg |
| Orai | Atgtcggtttggtcagccagtac | agccttcagtttcgccctacta |
| pbanR | tcttagggaatacaagcacat | atagcaatgtatcgctccatagt |
| caN | ctttcccagtctgataactat | tcacctacaaatggaagaga |
| GPAT | ATACATCAACCTCGGCTCGC | CCCAGACCACTTCAACGAC |
| per | agaatcaagcgaggcctgta | cggccaagaatatggctaaa |
| GPAT-f1 | GATCACTAATACGACTCACTATAGGGAGAACACATCGCAGTTATGCCGA | GATCACTAATACGACTCACTATAGGGAGACACGTGCTCCGCCACGTCCT |
| GPAT-f2 | GATCACTAATACGACTCACTATAGGGAGAAGGAAAGCTGGGCGCTTCA | GATCACTAATACGACTCACTATAGGGAGATGGGACTCGACCCGCTGCTGT |
| EGFP | GATCACTAATACGACTCACTATAGGGAGACCTGAAGTTCATCTGCACCAC | GATCACTAATACGACTCACTATAGGGAGACTCCAGCAGGACCATGTGATC |
| Rp49 | CAGGCGGTTCAAGGGTCAATAC | TGCTGGGCTCTTTCCACGA |

Rp49: ribosomal protein 49
